# Au LSPR-boosted S-scheme carbon nitride heterostructures for H_2_O_2_ photocatalytic production

**DOI:** 10.1039/d6ta04477h

**Published:** 2026-08-06

**Authors:** Auttaphon Chachvalvutikul, Haifeng Qi, Renjun Liu, Bo Hou, Man Yang, Simon Astley, D. Andrew Evans, Anatoly V. Zayats, David R. Richards, Ouardia Akdim, Graham J. Hutchings

**Affiliations:** a Max Planck-Cardiff Centre on the Fundamentals of Heterogeneous Catalysis FUNCAT, Cardiff Catalysis Institute, School of Chemistry, Cardiff University, Translational Research Hub Cardiff CF24 4HQ UK hutch@cardiff.ac.uk Akdimo@cardiff.ac.uk; b OEM Group, Department of Physics, Durham University South Road Durham DH1 3LE UK; c School of Physics and Astronomy, Cardiff University Cardiff CF24 3AA UK; d School of Materials Science and Engineering, Xi'an University of Technology Xi'an 710048 Shaanxi China; e Department of Physics, Aberystwyth University Aberystwyth Ceredigion SY23 3FL UK; f Department of Physics and London Centre for Nanotechnology, King's College, London Strand London WC2R 2LS UK

## Abstract

Photocatalytic oxygen reduction offers a sustainable, solar-driven route for the green synthesis of hydrogen peroxide (H_2_O_2_), however it suffers from limited efficiency due to rapid charge recombination and poor selectivity toward the two-electron reduction pathway. Herein, crystalline poly(heptazine) imide (PHI) and poly(triazine) imide (PTI) were synthesized *via* an ionothermal method and assembled into a PHI/PTI heterostructure, leading to a high H_2_O_2_ productivity of 1.27 ± 0.03 mmol L^−1^. The performance of the composite was benchmarked against the individual pristine components, graphitic carbon nitride (GCN) and carbon-doped GCN, with the heterostructure exhibiting superior activity attributed to an S-scheme charge-transfer mechanism promoting efficient charge separation. Au nanoparticles were subsequently deposited onto the heterostructure surface to yield a 1Au/PHI/PTI catalyst, which further enhanced the performance by *ca.* 74% through the S-scheme heterojunction, the Schottky barrier formation and the localised surface plasmon excitation. Under optimised continuous irradiation conditions, this catalyst achieved an apparent quantum yield of 18.8% and produced 8.32 ± 0.13 mmol L^−1^ H_2_O_2_ over 5 h, outperforming the recovering-and-reusing route. This work highlights the synergistic effect of Au LSPR with the PHI/PTI S-scheme heterostructure as a promising strategy for enhancing photocatalytic O_2_ reduction to H_2_O_2_, while also underscoring the importance of strong metal-support interactions for long-term catalyst stability.

## Abstract 2

Combining Au LSPR with S-scheme PHI/PTI heterojunction yields 8.32 ± 0.13 mmol L^−1^ H_2_O_2_ with an apparent quantum yield of 18.8%.

## Graphical abstract

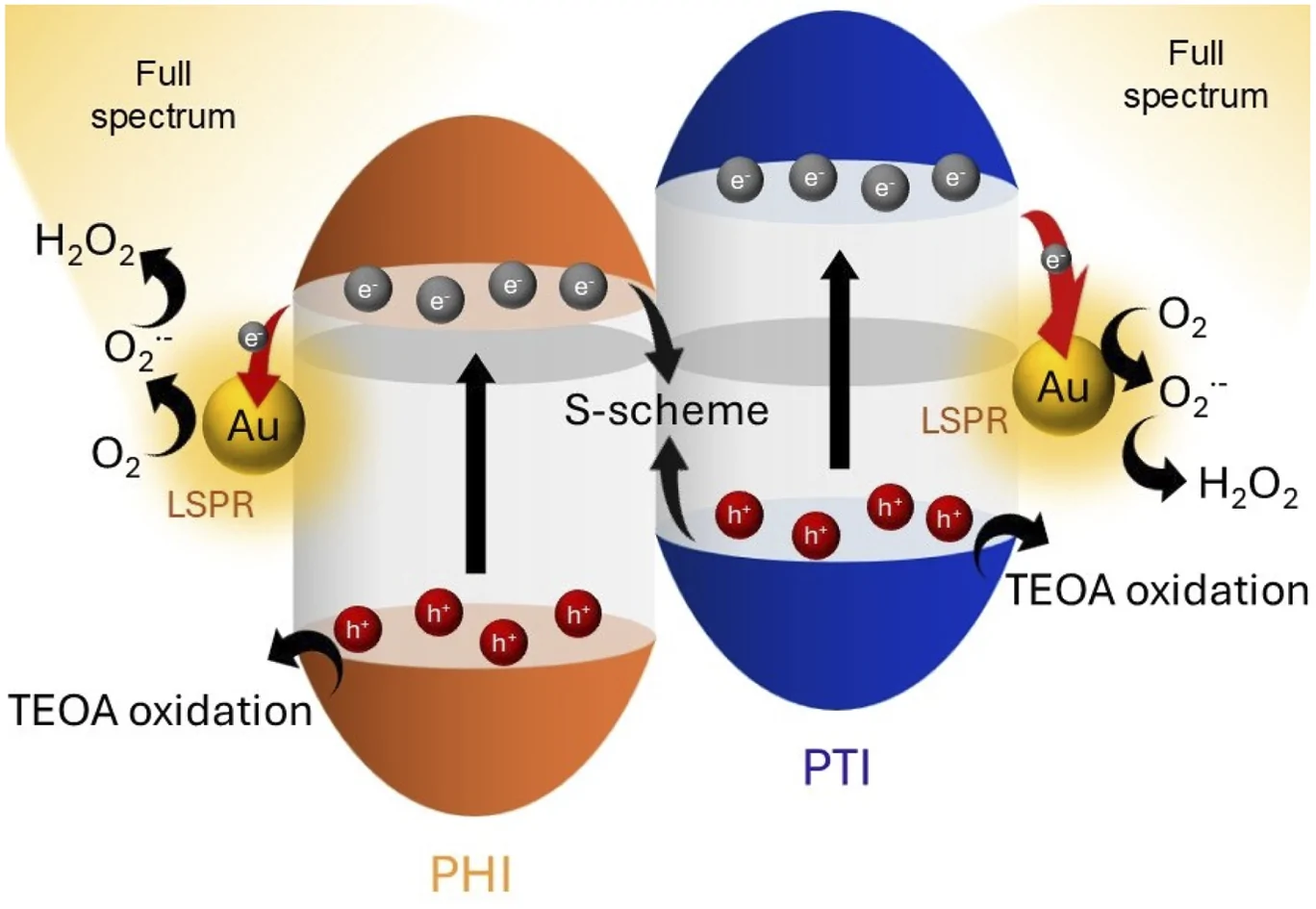

## 1. Introduction

Hydrogen peroxide (H_2_O_2_) is an essential chemical widely used in disinfection, chemical synthesis, environmental remediation, and as a potential energy carrier.^1,2^ Traditionally, H_2_O_2_ is synthesised *via* the anthraquinone process, where anthraquinone derivatives undergo hydrogenation to form hydroquinones, which are then oxidised using molecular oxygen to produce H_2_O_2_.^3^ Despite its efficiency, this process is energy-intensive, relies on high pressures and organic solvents, generates substantial waste, and requires high-concentration H_2_O_2_ production for cost-effective transport, raising safety concerns.^4^

The photocatalytic H_2_O_2_ production has emerged as a promising alternative, offering a greener and more cost-effective method. It operates under ambient conditions, eliminating toxic solvents and complex reactants while enabling scalability.^2,5^ Previous studies have highlighted that the photocatalytic H_2_O_2_ formation proceeds *via* a two-electron oxygen reduction reaction (ORR),^6,7^ and/or two-electron water oxidation reaction (WOR), where efficient charge separation and oxygen activation are key determinants of activity.^8^

Graphitic carbon nitride (g-C_3_N_4_) has gained attention due to its layered structure, thermal stability up to 600 °C, and visible-light absorption with a bandgap of ∼2.7 eV.^9,10^ It enables the selective two-electron oxygen reduction reaction to H_2_O_2_, minimising by-products and improving efficiency.^11^ However, due to limited light absorption and rapid charge recombination, g-C_3_N_4_ suffers from low quantum efficiency.^10^ Advanced polymeric carbon nitride materials, such as poly(heptazine) imide (PHI) and poly(triazine) imide (PTI), address these limitations. They are synthesised *via* ionothermal methods using ionic liquids or molten salts, which provide a high-temperature, solvent-free environment that facilitates the polymerisation of the molecular precursors.^12^ These materials exhibit high crystallinity, stability, and improved electronic conductivity, leading to enhanced photocatalytic performance.^13^ These modifications improve O_2_ reactivity and significantly boost H_2_O_2_ production rates compared to pristine g-C_3_N_4_.^14–18^ The PHI synthesis typically involves eutectic molten salts (*e.g.*, KCl or KCl/LiCl) under an inert atmosphere.^19,20^ PTI is synthesised *via* melamine calcination under static air, forming triazine-based imide frameworks.^21^ A PHI/PTI heterojunction synthesised by calcining a urea/KCl/LiCl mixture further enhances the photocatalytic H_2_ evolution compared to PHI or PTI alone.^21^ A more complex synthesis using ball-milling 5-aminotetrazole, melamine, LiCl, and KCl followed by calcination under N_2_ also yields a PHI/PTI heterostructure with an improved H_2_O_2_ production.^16^ Carbon doping further enhances the photocatalytic efficiency of PHI. Zhang *et al.* demonstrated that introducing malonic acid extends the PHI light absorption to 700 nm, significantly beyond the undoped PHI edge (∼450 nm).^20^ Additionally, decorating photocatalysts with noble metals like Au has proven highly effective in boosting the activity. Au nanoparticles extend light absorption into the visible and near-infrared regions through their localised surface plasmon resonance (LSPR) effect, which enhances charge-carrier separation, reduce recombination, and provide active sites for O_2_ reduction, thereby facilitating H_2_O_2_ production.^22–26^

Despite these advances, the development of a photocatalytic system that simultaneously achieves high H_2_O_2_ productivity, efficient utilisation of the precursors, and scalable synthesis remains challenging. Notably, the precursor conversion efficiency, and the environmental impacts associated with the material preparation are often overlooked in the evaluation of photocatalytic materials, despite their critical importance for practical and sustainable implementation. A key unresolved question is whether a PHI/PTI framework can provide sufficient electronic and structural advantages to serve as an effective support for plasmon-enhanced H_2_O_2_ production while maintaining favourable synthesis efficiency, reduced environmental impact, and economic viability.

In this study, carbon-doped PHI/PTI heterostructure was synthesised *via* a facile and scalable ionothermal method and characterised thoroughly. Urea was selected as the nitrogen-rich organic precursor owing to its low cost, commercial availability, and high nitrogen content. For comparison, carbon-doped g-C_3_N_4_, carbon-doped PHI and carbon-doped PTI were synthesised. The synthesis yields of the resulting materials were systematically evaluated alongside with their H_2_O_2_ photocatalytic production activity. The data reveal a high synthesis yield for the PHI/PTI system as well as a superior photocatalytic activity (1.27 ± 0.06 mmol L^−1^) compared to the other synthesised materials. This exceptional activity is associated with the formation of a heterojunction between PHI and PTI. Au was subsequently loaded on PHI/PTI (*i.e.*, 1Au/PHI/PTI) *via* photodeposition. The as-synthesised photocatalyst demonstrates a significant enhancement in H_2_O_2_ production (2.21 ± 0.06 mmol L^−1^ h^−1^) with an apparent quantum yield of 18.8% under full-spectrum irradiation (124 mW cm^−2^). The reaction parameters were optimised, including hole scavenger concentration, reaction atmosphere, and irradiation wavelengths (ranging from UV to mid-visible regions). Extended reaction time experiments simulated large-scale continuous H_2_O_2_ production, contrasting with conventional recovery, reusability, and stability tests.

## 2. Experimental procedure

### 2.1. Synthesis of g-C_3_N_4_ derivatives

Carbon-doped g-C_3_N_4_ (CGCN) and g-C_3_N_4_ derivatives were synthesised by pyrolysis of urea in the presence of additives according to the previous literature.^20^ In every condition, the amount of urea was 20 g, and the calcination condition was fixed at 600 °C for 3 hours with a ramp of 3 °C min^−1^. To synthesise the carbon-doped g-C_3_N_4_, malonic acid (0.60 g) was homogenously mixed with urea. Then, the powder mixture was placed into the crucible (100 mL) with a lid and covered with two layers of aluminium foil. Carbon-doped PHI, PTI, PHI/PTI heterostructure were synthesised by mixing urea (20 g) and malonic acid (0.06 g) with KCl (10 g), LiCl (10 g), or KCl (5.5 g)/LiCl (4.5 g), respectively. The eutectic KCl/LiCl salt mixture (55 : 45 wt%, mp ≈ 353 °C) forms a molten reaction medium below the calcination temperature, facilitating ionothermal condensation of carbon nitride frameworks, whilst such a process is limited by using pure KCl (MP ∼ 770 °C) and LiCl (610 °C), which remain in solid form.^12^ In comparison, pristine g-C_3_N_4_ was also synthesised using urea without any additives. After cooling to room temperature, the products were washed thoroughly with hot deionised water (2 L) and dried at 110 °C for 16 h.

### 2.2. Photodeposition of Au

Au nanoparticles were then deposited on PHI/PTI *via* photodeposition. The Au loading was fixed at 1 wt%, corresponding to a total amount 0.005 g. Firstly, 0.495 g of PHI/PTI was dispersed in 150 mL of methanol (150 mL, aqueous solution (25% v/v)) and sonicated for 30 min. Then, the HAuCl_4_·3H_2_O aqueous solution (403 µL, Au concentration of 12.4 mg mL^−1^) was added, followed by stirring (800 rpm) for an additional 30 min under a N_2_ flow (150 mL min^−1^). The suspension was then irradiated with an Xe lamp (300 W, 124 mW cm^−2^; Newport, LHS302) under magnetic stirring (800 rpm) for 1 h. The distance between the lamp and the solution was 10 cm. Finally, the resulting powder was collected by filtration, washed with deionised water (500 mL), and dried at 110 °C overnight. The synthesised catalyst with Au nanoparticles (average diameter *ca.* 5.4 nm) was denoted as 1Au/PHI/PTI. The actual Au loading was determined by ICP-MS to be 0.65 wt%.

### 2.3. Characterisations

The crystal phases of the photocatalysts were analysed using an X-ray diffractometer (XRD, PANalytical X'PertPRO) with Cu Kα radiation (*λ* = 1.540598 Å). The microstructure and morphology were observed using transmission electron microscopy (TEM, JEOL JEM-2100) and aberration-corrected high-angle annular dark-field scanning transmission electron microscopy (AC HAADF-STEM, Spectra).

The chemical composition and oxidation states of the surface species were determined using an X-ray photoelectron (XPS, K-alpha, Thermo Fisher Scientific) using Al K_α_ X-ray source at 160 eV. XPS spectra were corrected by referencing the binding energy of an adventitious carbon (C 1s) peak at 284.8 eV. The electron-transfer direction was examined using light-irradiated *in situ* XPS. A full-spectrum irradiation was used with a Bentham TLS120xe tunable light source, with focusing optics to illuminate an 8 mm diameter circle, ensuring the entire detection region is illuminated. The temperature recorded before and after light exposure showed no increase, and measurements were taken throughout.

Fermi levels and work functions were determined by Ultraviolet Photoelectron Spectroscopy (UPS) using a Thermo Scientific Nexsa XPS/UPS System with monochromatic He I radiation (21.21 eV) and Au as a reference. Powder samples were deposited onto fluorine-doped tin oxide (FTO) glass by spin coating. Briefly, samples (0.020 g) were dispersed in methanol (1 mL) by sonication for 30 min, and 100 µL of suspension was spin-coated at 3000 rpm for 45 s, followed by drying at 90 °C for 30 min.

The work function (*Φ*) was calculated from the difference between the photon energy and the secondary electron cutoff (*E*_cutoff_), measured with a 9 eV bias:*Φ* = 21.21 − *E*_cutoff_

The energy difference between the Fermi level and the valence band maximum (Δ*E*) was obtained by extrapolating the valence band spectrum. The valence band potential (*E*_VB_) and conduction band potential (*E*_CB_) were calculated using:*E*_VB_ = −(*Φ* + Δ*E*)*E*_CB_ = *E*_VB_ + *E*_g_

Energies referenced to the vacuum level were converted to the Normal Hydrogen Electrode (NHE) scale using:*E* (eV *vs.* vacuum) = −4.5 − *E* (V *vs.* NHE).

Chemical groups and bonding characteristics were analysed through Attenuated Total Reflectance Fourier-transform infrared spectroscopy (ATR-FTIR, Frontier, PerkinElmer). Optical absorption properties were evaluated using a UV-Visible diffuse reflectance spectrophotometer (SS UV-Vis DRS, Agilent Cary 4000), employing BaSO_4_ powder as the reflectance standard.

Steady-state photoluminescence spectrum (PL), and photoluminescence quantum yield (PLQY), a ratio of the number of photons emitted as luminescence to the number of incident photons, were determined by UV-NIR Absolute PL Quantum Yield Spectrometer (Quantaurus-QY Plus C13534, Hanamatsu) using excitation wavelength (*λ*_ex_) of 360 nm. The measurements were performed by loading a powder sample into a quartz sample holder and using an empty holder as a blank.

The specific surface area was determined by the Brunauer–Emmett–Teller method (BET) using N_2_ adsorption–desorption isotherms measured at 77 K on a Quantachrome Quadrasorb Evo Analyser. Samples (∼0.150 g) were degassed under vacuum at 120 °C for 4 h prior to analysis. Elemental concentrations of Au, K, Li and Cl were measured by Inductively Coupled Plasma Mass Spectrometry using an Agilent 7900 ICP-MS with an I-AS autosampler. Samples (0.05 g) were digested in aqua regia (4 mL) overnight, diluted to 50 mL with deionised water and filtered. Measurements were performed in quadruplicate using calibration curves from standard solutions, with a blank digestion as a control.

Solid-state ^13^C MAS NMR spectra were recorded on a Bruker Avance III 400 MHz NMR Spectrometer. Powder samples were packed in 4 mm zirconia rotors and spun at 12 kHz. Cross-polarisation experiments were performed with a 2 ms contact time, a 5 s relaxation delay, and a 0.035 s acquisition time (500 scans, RF field at 100 kHz). Chemical shifts were referenced to tetramethylsilane using α-glycine as a secondary standard.

For radical detection, 7.5 mM 5,5-dimethyl-1-pyrroline *N*-oxide (DMPO) was added to the photocatalytic mixture (80 mL containing 0.5% v/v TEOA/H_2_O and 0.008 g catalyst). The suspension was sonicated for 5 min, purged with O_2_ for 30 min, and irradiated with a 300 W Xe lamp for 30 min. X-band continuous-wave EPR spectra were recorded at room temperature on a Bruker EMX Micro EPR Spectrometer equipped with a Bruker ER4123-D dielectric resonator.

Electrocatalytic oxygen reduction reaction performance was measured using a three-electrode potentiostat electrochemical workstation (SP-150, BioLogic), with a rotating ring disk electrode (RRDE). The working electrode was fabricated by the drop-casting method as follows: 0.010 g of the sample was mixed with 500 µL of deionised water, 500 µL of isopropanol, and 250 µL of xanthan gum aqueous solution (10 w/v%) and subsequently sonicated for 10 min. The homogeneous mixture (5 µL) was then dropped onto the surface of the RRDE (glassy carbon/Pt) with a sample diameter of 3 mm. Then, the electrodes were dried at room temperature for 30 min. Subsequently, linear sweep voltammetry (LSV) was employed at different rotating speeds *i.e.*, 100, 500, 1000, and 2000 rpm. The scan range was 0.1 to −0.7 V *vs.* Hg/HgO with a scan speed of 10 mV s^−1^. The number of electrons (*n*) in the oxygen reduction reaction was derived from the Koutecky–Levich (K–L) plot as follows:^27^
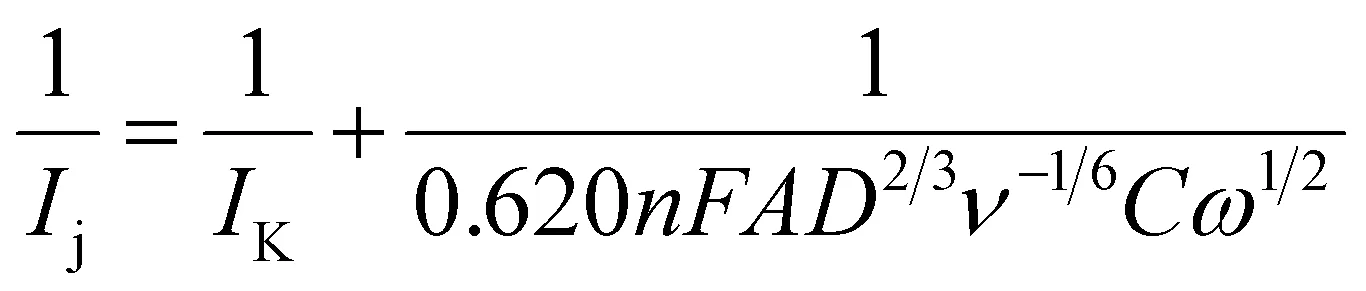


The measured current (*I*_j_) includes the kinetic current density (*I*_K_), which can be obtained from the intercept of the plot of the reciprocal current (1/*I*_j_) against the reciprocal root of the angular rotation rate (*ω*^−1/2^).

### 2.4. Photocatalytic H_2_O_2_ production and decomposition tests

Photocatalytic O_2_ reduction to H_2_O_2_ in aqueous solution was evaluated using a sealed round-bottom flask reactor under UV-Vis irradiation. In a typical experiment, 0.008 g of catalyst was dispersed in 80 mL of 5 v/v% triethanolamine (TEOA) aqueous solution by sonication for 15 minutes. The suspension was then purged with O_2_ (150 mL min^−1^) for 30 minutes under stirring (800 rpm). After that, the suspension was irradiated with UV-Vis light generated from a Xe lamp (300 W, 124 mW cm^−2^; Newport, LHS302) located at the top of the reactor with a distance of 10 cm otherwise specifically stated. The reaction is performed under room temperature and ambient pressure. At specific time intervals, 1.5 mL of the liquid sample is taken and filtered with 0.2 µm Millipore filter to remove the photocatalyst and then mixed with 1.5 mL of 0.1 M Potassium Titanium Oxalate Solution (PTOS) and aged for 20 min. The coloured solution is then analysed by UV-Vis spectrophotometer (Cary 4000, Agilent). The absorbance at 400 nm was monitored, and the amount of H_2_O_2_ was calculated against the calibration curve. For the photocatalytic H_2_O_2_ decomposition, a similar set-up was used. Typically, a specific amount of H_2_O_2_ solution (50 wt%) was added to make the initial H_2_O_2_ concentration of 500 mg L^−1^ (14.7 mmol L^−1^) prior to being stirred with the photocatalyst, in the dark under O_2_ flow. Then the light is switched-on and the degradation of H_2_O_2_ is monitored over time. Reactive oxygen species trapping experiments were performed under identical conditions by adding various scavengers to identify the active species involved in the photocatalytic process. *Tert*-butanol (*t*-BuOH), *p*-benzoquinone (BQ), and sodium azide (NaN_3_) were used as scavengers for ˙OH, ˙O_2_^−^, and ^1^O_2_, respectively.^28–30^ The H_2_O_2_ production performance was then compared with the control experiment without scavengers.

### 2.5. Apparent quantum yield (AQY) calculation

The apparent quantum yield (AQY) or apparent quantum efficiency (AQE) is calculated as follows:^31^
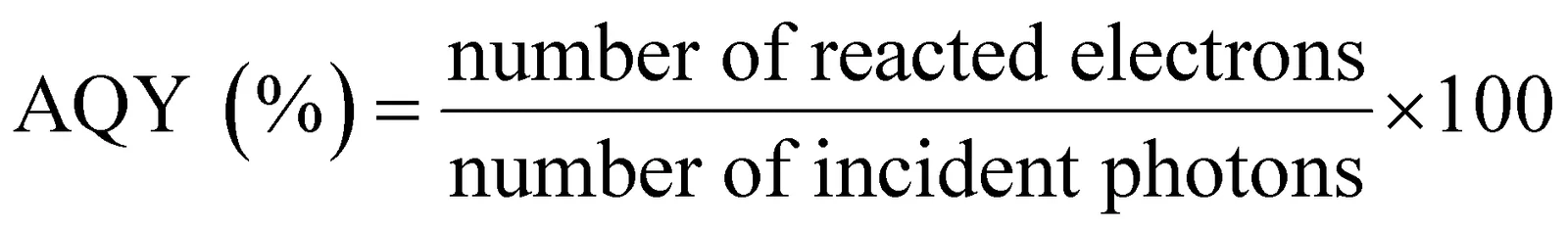


The moles of the incident photons reaching the reactor was calculated by ferrioxalate actinometry.^32^ In a typical procedure, 37.5 mL of 0.02 M Fe_2_(SO_4_)_3_ solution is mixed with 37.5 mL of 0.12 M K_2_C_2_O_4_ solution and diluted with 75.0 mL of deionised water to mimic the actual reaction. The solution containing the ferrioxalate complex is then irradiated for 60 seconds using similar illumination conditions as in the activity tests. Afterwards, 400 µL of the resultant mixture is mixed with 200 µL of 1,10-phenanthrolin solution (0.2 wt%) and 200 µL of acetate buffer solution (pH = 4.5, prepared by dissolving 2.05 g CH_3_COONa to 25 mL H_2_SO_4_ solution (0.184 M)). The red solution is then diluted to 4 mL with deionised water and subsequently kept in the dark for 30 min. After the reaction, the ferrous ion concentration was determined by UV-Vis spectrophotometry (Cary 60, Agilent) and monitored with absorbance measurements recorded at a characteristic wavelength of 510 nm. Accordingly, the moles of Fe^2+^ was calculated by the following equation:
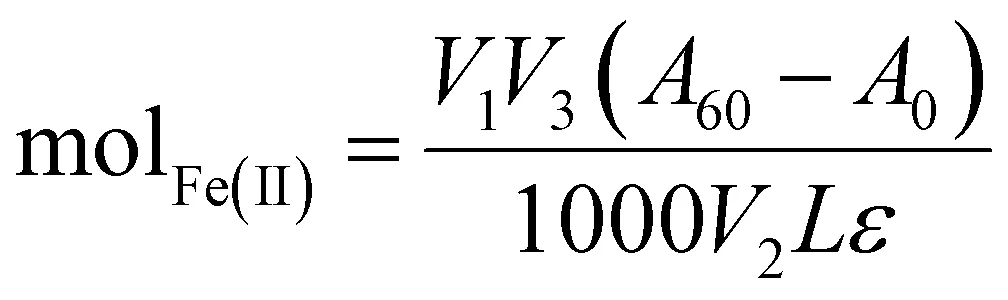
where *V*_1_, *V*_2_, and *V*_3_ are irradiation volume (80 mL), sample taken (0.2 mL), and diluted final volume (4 mL), respectively. *A*_60_ and *A*_0_ are an absorbances after 60 seconds of irradiation and prior to irradiation, respectively. *L* is path length (1 cm), and *ε* is an absorption coefficient at 510 nm (1.11 × 10^4^ L mol^−1^ cm^−1^).

The number of photons per minute are calculated using the following equation:
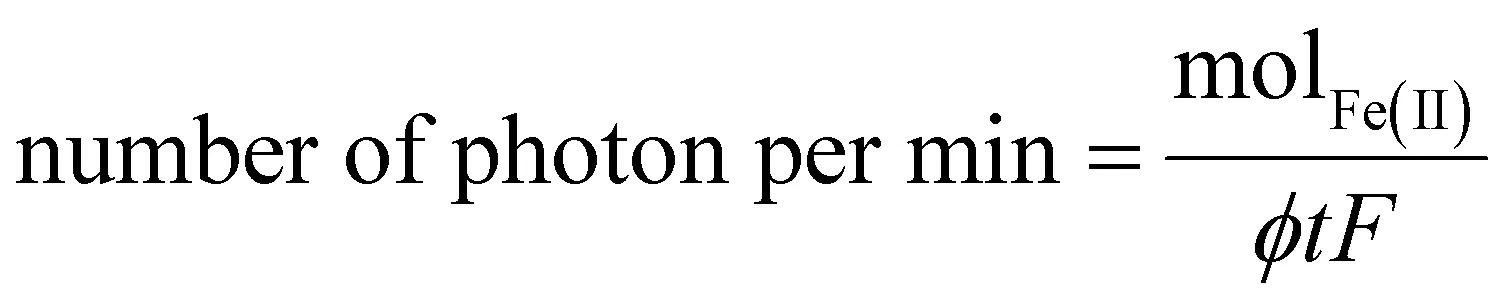
where *Φ* is the quantum yield for Fe(ii) (1.21). *t* is the reaction time (1 min). *F* is the mean fraction of light absorbed (1). Under the reaction condition, the number of photon per min is 3.10 × 10^−5^.

Subsequently, the AQY of H_2_O_2_ production can be calculated using the following formula:^33^
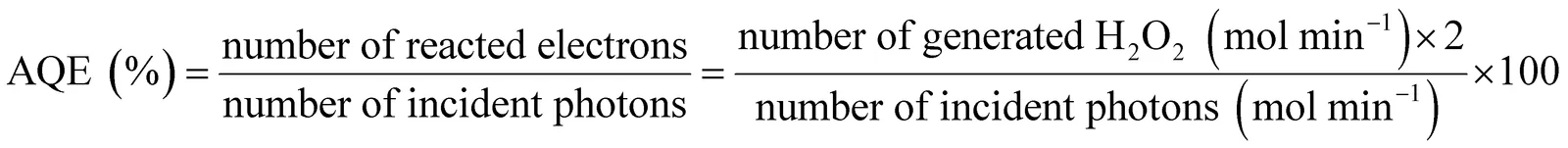


## 3. Results and discussion

### 3.1. Structural characterisations

**Fig. 1:**
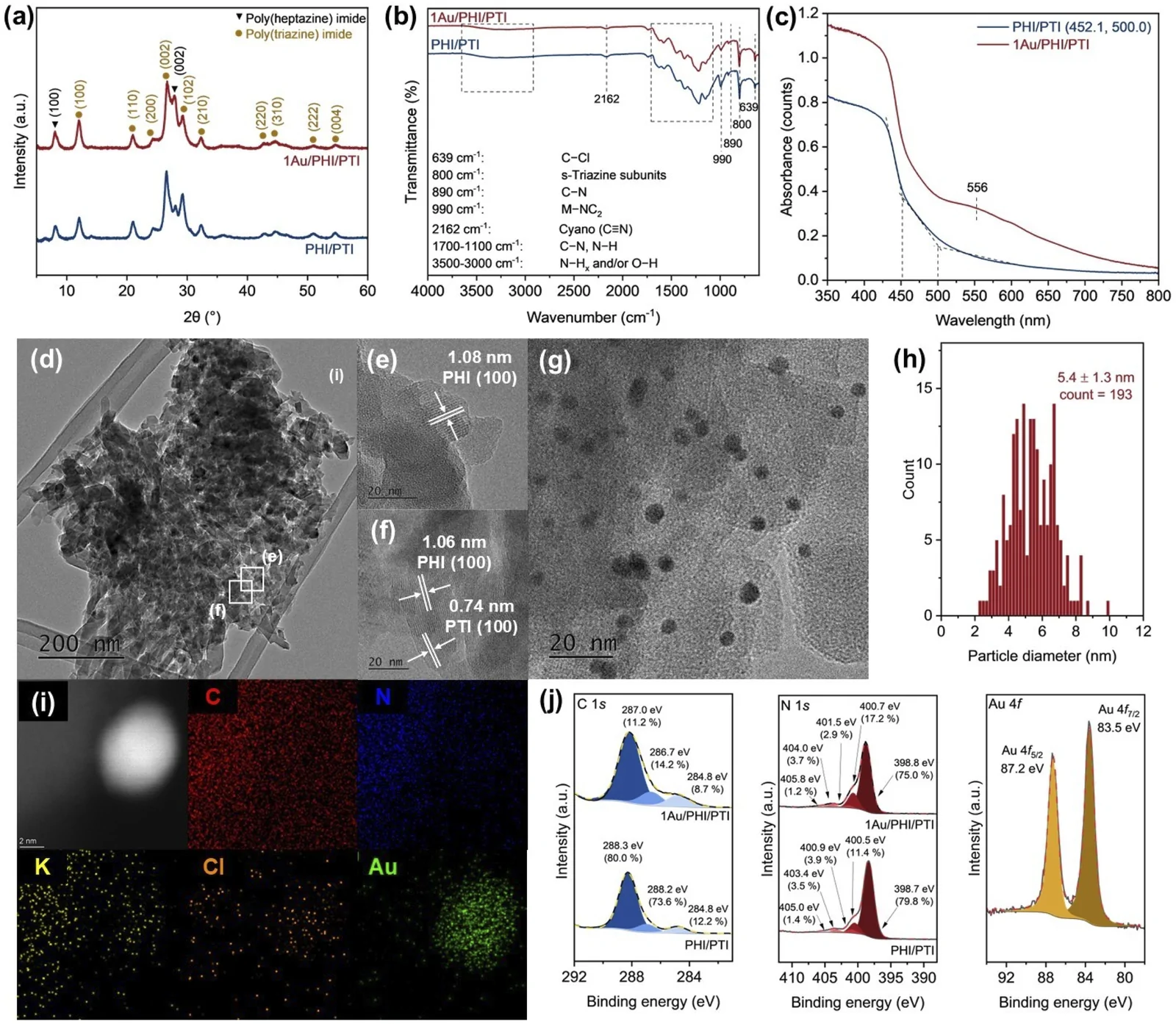
(a) XRD, (b) ATR-FTIR, (c) SS UV-Vis DRS results of PHI/PTI and 1Au/PHI/PTI. TEM images of (d)–(f) PHI/PTI and (g) 1Au/PHI/PTI. (h) Au particle size distribution. (i) HAADF AC-STEM and EDX mapping of Au/PHI/PTI. (j) High-resolution C 1s, and N 1s spectra of PHI/PTI and 1Au/PHI/PTI, and a Au 4f spectrum of 1Au/PHI/PTI.

The samples were first characterised by XRD (Fig. 1a and S1). GCN exhibits two main diffraction peaks at 12.8° and 27.6°, corresponding to the (100) and (002) crystal planes of g-C_3_N_4_ (JCPDS no. 87-1526). The 12.8° peak represents the in-plane structural packing of the tri-*s*-triazine (heptazine) motifs (0.66 nm spacing), while the 27.6° peak corresponds to the interlayer stacking of the aromatic heptazine units (0.34 nm spacing), confirming the heptazine-based structure of g-C_3_N_4_.^34^ The CGCN pattern closely matches GCN, indicating that malonic acid addition does not alter the graphitic structure. For PHI, the diffraction peaks shift to 8.1° and 28.3° ((100) and (002) planes),^20,35,36^ indicating an expanded in-plane heptazine arrangement (1.1 nm) and a reduced π–π stacking distance (0.32 nm), reflecting a denser packing and a more complete polymerisation than GCN. PTI displays distinctly different peaks at 12.1°, 21.1°, 24.4°, 26.8°, 29.2°, 32.3°, 42.8°, 44.6°, and 54.7°, corresponding to the (100), (110), (200), (002), (102), (210), (220), (310), and (004) planes.^14,21,37^ The PHI/PTI composite shows diffraction peaks from both PHI and PTI, although with the (100) plane of PHI and the (110) and (102) planes of PTI exhibiting increased intensity. A new peak at 50.9° ((222) plane of PTI) appears, indicating an enhanced crystallinity due to the eutectic KCl/LiCl mixture. At 600 °C, this molten salt mixture (KCl/LiCl molar ratio of 0.59) dissolves urea, promoting condensation and yielding higher crystallinity. Compared with pristine PHI and PTI, the (002) peak of PHI shifts slightly from 28.2° to 28.0° in the PHI/PTI system. Similarly, the (002) peak of PTI shifts slightly from 26.8° to 26.6° in the PHI/PTI system. This suggests the formation of a heterojunction between the two components.^38^

The 1Au/PHI/PTI sample displays a pattern similar to PHI/PTI, without the notable presence of Au peaks. This may be attributed to the low gold loading,^39^ small Au particle size,^23,40^ and/or high dispersion of Au.^23^ However, the intensity of the (002) plane of PHI (28.0°) increases and the intensity of the (102) plane of PTI (29.2°) decreases simultaneously without any obvious shift in the diffraction angles compared to the PHI/PTI heterojunction. These changes suggest that Au is deposited on the surface of both components in the PHI/PTI system.

The functional groups and structures of all the samples were then analysed *via* Attenuated Total Reflectance Fourier-Transform Infrared (ATR-FTIR) spectroscopy (Fig. 1b and S2). Pristine GCN exhibits characteristic peaks at 800 cm^−1^ and 890 cm^−1^ (C–H and C–N bonds in heptazine rings), a broad band at 1100–1700 cm^−1^ (N-containing heterocycles: N–C=N, C–NH_2_, and C–N_3_), and a 3000–3500 cm^−1^ region (O–H and N–H stretching, indicating adsorbed water).^21,41–43^ The FTIR spectrum of CGCN is very similar to GCN, although with reduced intensity of the peaks at 890 and 3000–3500 cm^−1^, indicating a lower N content in the heptazine units due possibly to N substitution by C. PHI displays additional peaks at 990 cm^−1^ and 2162 cm^−1^, assigned to M–NC_2_ (possibly K–NC_2_) and terminal cyano (C≡N) groups,^21,44^ respectively. The M–NC_2_ signal suggests the presence of residual metal species, despite washing, while the cyano group indicates a replacement of partially uncondensed –NH_2_ groups from GCN. This is supported by the weaker N–H band at 3000–3500 cm^−1^ in PHI compared to GCN. In PTI, the 1100–1700 cm^−1^ region shows sharper bands at 1267, 1372, and 1448 cm^−1^, characteristic of triazine units,^45^ confirming a triazine-based structure. Additionally, a strong band at 639 cm^−1^, attributed to C–Cl bonds, indicates residual chlorine in the structure.^46–48^ PTI also exhibits a weaker 2162 cm^−1^ band than PHI, implying fewer cyano groups. The PHI/PTI and 1Au/PHI/PTI spectra combine signals from both PHI and PTI, reflecting a mixed structure. Overall, the distinct FTIR features of PHI, PTI, and PHI/PTI, particularly the terminal cyano group, indicate structural differences from GCN, supplementing the XRD data.

The light absorption properties of the samples were investigated using UV-Vis DRS (Fig. 1c and S3a–c). Pristine GCN exhibited the lowest light absorption, with an absorption edge at *ca.* 450 nm (2.76 eV). The carbon-doping strategy (CGCN) extended the absorption edge to 485.1 nm (2.56 eV), indicating enhanced visible-light harvesting compared to the pristine GCN. PHI displayed an absorption edge at 464.8 nm (2.67 eV) and showed stronger visible-light absorption than GCN and CGCN. In contrast, PTI exhibited a UV absorption band with an absorption edge at 444.0 nm (2.79 eV) and a tail absorption extending from 400 to 550 nm. The improved absorption in PHI and PTI is attributed to increased π-electron delocalisation resulting from the higher degree of polymerisation achieved during ionothermal synthesis.^49,50^ The PHI/PTI composite showed absorption features characteristic of both materials, including PTI absorption around 452 nm, with a tail extending to 550 nm, and PHI absorption near 500 nm. The shift in the PHI absorption edge is attributed to the higher degree of condensation achieved using a LiCl/KCl eutectic mixture rather than KCl alone during the ionothermal synthesis. After Au deposition to form 1Au/PHI/PTI, light absorption increased further, particularly around 556 nm due to the LSPR of Au nanoparticles. The characteristic PTI absorption was not clearly observed because of the overlapping with the LSPR absorption of Au. The indirect band gaps (*E*_g_) were estimated using the Kubelka–Munk function and Tauc plots (Fig. S3b and c). The *E*_g_ values of GCN, CGCN, PHI, and PTI were determined to be 2.83, 2.53, 2.67, and 2.73 eV, respectively, consistent with their absorption edges. The PHI/PTI composite exhibits two absorption transitions associated with the PHI and PTI phases (Fig. S3c), whereas only the absorption transition associated with PTI was observed for 1Au/PHI/PTI due to the aforementioned overlap. Overall, the light absorption of GCN can be enhanced through carbon doping (CGCN) and structural modification to more crystalline frameworks such as PHI and PTI.

The morphology and microstructure of the samples were examined by transmission electron microscopy (TEM), as shown in Fig. 1d–g and S4. GCN exhibited a typical curly sheet morphology, while CGCN showed a similar structure with no obvious morphological change despite its higher carbon content. In contrast, PHI consisted of nanosheets without a curvy morphology and displayed clear lattice fringes with a spacing of ∼1.05 nm, corresponding to the (100) crystal plane. PTI showed a distinct large-sheet morphology composed of stacked nanosheets, with lattice fringes of ∼0.71 nm attributed to the (100) plane. The PHI/PTI composite (Fig. 1d–f) retained a nanosheet-based morphology, with lattice spacings of 1.06–1.08 nm corresponding to PHI (100) (Fig. 1e) and ∼0.74 nm corresponding to PTI (100) (Fig. 1f). These increased lattice spacings of PHI and PTI in the heterojunction further confirmed the formation of lattice tensile strains in PHI and PTI associated to the formation of an heterojunction between the two components as confirmed by XRD (Fig. S1). In comparison, no lattice fringes were observed for GCN and CGCN, indicating lower crystallinity. These results confirm that the ionothermal method promotes crystallisation, producing PHI and PTI with higher crystallinity than GCN and CGCN. Although diffraction peaks corresponding to Au were not observed in the XRD pattern, the presence of Au on PHI/PTI was confirmed by TEM, and HAADF AC-STEM EDX mapping. TEM analysis (Fig. 1g) shows highly dispersed nanoparticles on the PHI/PTI surface with an average size of 5.4 ± 1.3 nm (Fig. 1h). HAADF AC-STEM imaging and EDX mapping further (Fig. 1i) confirm the presence of Au.

N_2_ adsorption–desorption measurements (Fig. S5) show that GCN has a surface area of ∼71 m^2^ g^−1^ with a type IV isotherm and H3 hysteresis loop, while CGCN exhibits a lower surface area (42.0 m^2^ g^−1^) with an H4 loop, indicating slit-like pores. PHI displays a surface area of 51.8 m^2^ g^−1^, whereas PTI and PHI/PTI exhibit larger surface areas of ∼95 m^2^ g^−1^. Au deposition (1Au/PHI/PTI) does not significantly alter the catalyst's surface area (93.4 m^2^ g^−1^) compared to the bare PHI/PTI.

XPS survey spectra (Fig. S6) show C, N, and O in all samples, with PHI showing K and PTI showing Cl, consistent with ATR-FTIR results. Both K and Cl appeared in PHI/PTI and 1Au/PHI/PTI, with the latter also showing Au signal, confirming the successful deposition of Au. No impurity signals were observed, indicating high purity. The deconvoluted N 1s spectra (Fig. 1j and S7) show three main signals at ∼399, 400, and 401 eV, corresponding to sp^2^ pyridine N (C=N–C), tertiary bridging N (N–(C)_3_), and sp^3^ terminal N (C–N–H), respectively. Satellite signals at 404.1 and 405.4 eV result from π-excitation of the g-C_3_N_4_ structure.^51^ GCN, CGCN, and PHI display similar N 1s peaks and binding energies. However, PTI lacks the peak at 400 eV, indicating the absence of the tertiary bridging N, characteristic of the heptazine units.^52,53^ Moreover, Table S2 shows the C=N–C/C–N–H ratio is ∼7 for GCN, CGCN, and PHI, matching the heptazine structure (ideal ratio: 6), while PTI shows a ratio of 2, indicating the presence of triazine units.^45,53^ PHI/PTI and 1Au/PHI/PTI display all PHI and PTI signals, confirming their coexistence. The high-resolution XPS results align with the XRD and FTIR data, confirming the presence of heptazine units in PHI and triazine units in PTI. The C 1s spectra (Fig. 1j and S8) show four peaks at 284.7, 285.8, 288.2, and 293.7 eV, corresponding to sp^3^ adventitious carbon (C–C), C–O from adsorbed water/hydroxide groups, aromatic sp^2^ C (C–N_3_) in the heterocyclic rings, and π-excitation, respectively. The dominant signal in all samples is from the aromatic sp^2^ C (C–N_3_). CGCN shows a higher C–C contribution (8.9%) and a lower C–N contribution (79.5%) than GCN (2.5% and 96.5%, respectively), indicating N substitution in CGCN, consistent with the FTIR results. Unlike the N 1s spectra, the C 1s spectra show no notable differences between samples. Moreover, high-resolution K 2p spectra (Fig. S9a) show K 2p_3/2_ and K 2p_1/2_ peaks at 293 eV and 296 eV, respectively, consistent with K coordination to heptazine N atoms in PHI, PHI/PTI, and 1Au/PHI/PTI.^21,48,54^ Although the Li signals are absent in the survey, high-resolution Li 1s spectra (Fig. S9b) reveal a peak at ∼55 eV in PTI, PHI/PTI, and 1Au/PHI/PTI.^21,46^ Additionally, high-resolution Cl 2p spectra (Fig. S9c) show a doublet at 198 eV and 200 eV, indicating Cl spin–orbit coupling.^21,55,56^ PHI exhibits weaker Cl signals than PTI and PHI/PTI, suggesting lower Cl content. Overall, PHI contains K, PTI has Li and Cl, and PHI/PTI incorporates K, Li, and Cl. The oxidation state of Au in the 1Au/PHI/PTI sample was determined by a high-resolution Au 4f spectrum (Fig. 1j). Two deconvoluted peaks at 83.5 and 87.2 eV, corresponding to Au 4f_7/2_ and Au 4f_5/2_, indicate the presence of metallic Au, consistent with the previously reported Au/g-C_3_N_4_ systems.^57–59^ A more negative binding energy of Au compared to the bulk Au (84 eV)^60^ is observed, suggesting a charge transfer between Au and the support to equilibrate their different Fermi levels as expected in metal–semiconductor systems.

The actual Au loading of 0.65 wt% is significantly lower than the theoretical value of 1 wt%, which is attributed to the photodeposition method used in this study. This discrepancy may arise from the limited light absorption by the Au precursor species, which restricts their photoreduction and subsequent deposition onto the semiconductor surface.^61^ The precursor species that remain unreduced during the photodeposition process are removed during the subsequent filtration and washing steps. Finally, the C/N ratios determined by XPS and CHN analyses (Table S3) confirm higher C/N ratios in the CGCN and PHI/PTI samples than in the others, indicating that the higher carbon content in these samples resulted from the C-doping strategy.

Since XRD, ATR-FTIR, and XPS revealed structural differences among GCN, CGCN, PHI, and PTI, solid-state ^13^C–^1^H cross-polarisation magic-angle spinning (CP/MAS) NMR was used to further confirm their structures (Fig. S10). GCN exhibits three signals corresponding to different carbon in the heptazine unit: 156.8 ppm (C_1_, C–N_3_), 163.3 ppm (C_2_, tertiary carbon linking two heptazine units), and 165.3 ppm (terminal carbon C_3_ bonded to two N atoms, HN_2_–CN_2_).^62–64^ These signals are less intense in CGCN due to reduced crystallinity caused by carbon doping. PHI shows two main signals at 156.7 and 163.4 ppm, attributed to the heptazine tertiary carbon (C_1_, C–N_3_) and the bridging carbon between heptazine units. The absence of the terminal carbon signal indicates a higher degree of condensation compared with GCN. However, since XPS confirmed the presence of K in PHI, which has been reported to bond with the negatively charged bridging N atoms between heptazine units,^19,65^ high-power decoupling (HPDEC) ^13^C NMR was performed (Fig. S11), revealing an additional signal at 168.1 ppm corresponding to carbon bonded to unprotonated nitrogen atoms (C_3_).^66,67^ In contrast, PTI displays three distinct signals from PHI at 157.9, 163.7, and 168.8 ppm, corresponding to the triazine carbon (C_1_, C–N_3_), carbon adjacent to NH groups (C_2_, N_2_–C–NH), and carbon adjacent to unprotonated nitrogen atoms (C_3_, N_2_–C–N–Li^+^), respectively.^66,68^ The PHI/PTI composite shows signals from both PHI and PTI, confirming their coexistence. According to the above characterisations and previous reports, the chemical structures of GCN, PHI, and PTI can be shown in Fig. S12.

### 3.2. Photocatalytic performance

**Fig. 2:**
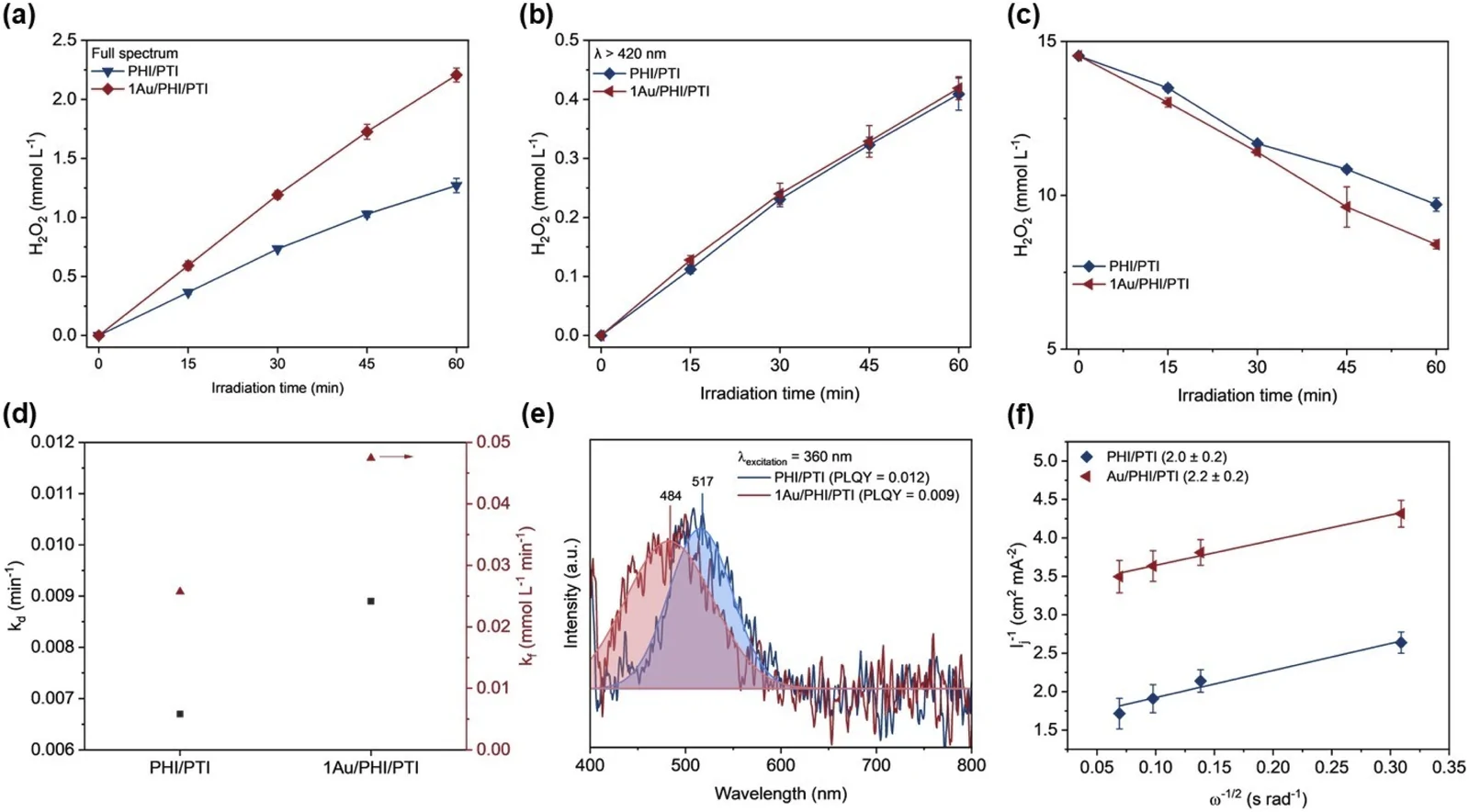
H_2_O_2_ production by PHI/PTI and 1Au/PHI/PTI under (a) UV-visible-NIR and (b) visible-NIR (*λ* > 420 nm) irradiation. (c) Photocatalytic H_2_O_2_ decomposition, (d) decomposition (*k*_d_) and formation (*k*_f_) rate constants, (e) steady-state PL spectra, and (f) the Koutecky–Levich plots of PHI/PTI and 1Au/PHI/PTI.

The photocatalytic activity of the as-prepared samples for H_2_O_2_ production was evaluated under UV-Vis irradiation using triethanolamine (TEOA, 5 vol%) as a sacrificial agent, in an O_2_-saturated solution (Fig. 2a and S13). For all catalysts, no H_2_O_2_ was detected in the dark. After 1 h irradiation, CGCN produced more H_2_O_2_ (0.77 ± 0.04 mmol L^−1^) than GCN (0.56 ± 0.03 mmol L^−1^), indicating the positive effect of carbon doping. In contrast, PTI showed the lowest activity (0.34 ± 0.01 mmol L^−1^). PHI exhibited the highest activity among the metal-free samples, producing 1.69 ± 0.03 mmol L^−1^. The PHI/PTI system produced 1.27 ± 0.06 mmol L^−1^. This value seems apparently lower than the pristine PHI. To enable a fair comparison between the two systems, we first quantified the PHI content in the PHI/PTI system and ensured that control experiments were performed using an equivalent amount of PHI (2.5 mg). Under these conditions, PHI alone produces only 0.47 ± 0.03 mmol L^−1^ of H_2_O_2_, which is lower than that achieved by the PHI/PTI system, confirming the beneficial role of the PHI/PTI heterojunction. In addition, we also examined a physical mixture of PHI and PTI containing the same proportions of each component as in the heterojunction. Interestingly, this physical mixture shows a substantially lower H_2_O_2_ yield than both PHI alone and the PHI/PTI heterojunction. This indicates that the simple coexistence of the two components is insufficient and that the interfacial architecture is crucial for performance enhancement (Fig. S14).

Besides, PHI/PTI remains more economically viable due to its significantly higher synthesis yield (23.0%) compared with PHI (2.8%). Indeed, the overall yield of catalyst obtained from the synthesis is one of the key parameters that directly influences the cost-effectiveness and sustainability of the material production when assessing the potential for large-scale production and practical implementation.

After Au deposition, 1Au/PHI/PTI showed the highest H_2_O_2_ production (2.21 ± 0.06 mmol L^−1^). To further clarify the contribution of Au nanoparticles and their interaction with the PHI/PTI heterostructure, 1Au/PHI was evaluated as a control sample (Fig. S15). The 1Au/PHI catalyst produced 0.54 ± 0.01 mmol L^−1^, corresponding to only a 15% enhancement compared with the pristine PHI (0.47 ± 0.03 mmol L^−1^), while Au deposited on the heterojunction increased the activity by *ca.* 74%. These results demonstrate that the promotional effect of Au nanoparticles is significantly amplified in the PHI/PTI heterostructure. Under visible-light irradiation (*λ* > 420 nm) (Fig. 2b and S16), all samples produced lower H_2_O_2_ levels than under full-spectrum irradiation, although the activity trend remained similar, except that PHI/PTI and 1Au/PHI/PTI showed comparable activity. This suggests that the highest efficiency requires a simultaneous excitation of the support and the Au LSPR, which is achievable under full-spectrum irradiation.

Photocatalytic activity depends on various factors, including surface area, light absorption, charge separation, and so on. However, surface area (Fig. S5) does not correlate with the photocatalytic activity. In terms of light absorption (Fig. 1c and S3), CGCN shows greater light absorption than GCN, which correlates with its higher photocatalytic activity, indicating that carbon doping is an effective strategy to enhance visible-light harvesting and performance. In the case of 1Au/PHI/PTI, the photocatalytic H_2_O_2_ productivity under full-spectrum irradiation is significantly higher than that of PHI/PTI despite their similar surface areas. UV-Vis DRS analysis shows a significantly enhanced light absorption at 556 nm, attributed to Au LSPR, which is likely responsible for the enhanced photocatalytic activity. The plasmon enhancement effect of Au is supported by the fact that the H_2_O_2_ productivity under full-spectrum where both PHI/PTI and Au are simultaneously excited (2.21 ± 0.06 mmol L^−1^) is higher than the addition of the activity under 250–400 nm (1.55 ± 0.08 mmol L^−1^) and 455 nm (0.09 ± 0.01 mmol L^−1^), where only PHI/PTI support or Au LSPR is activated.

In photocatalytic H_2_O_2_ production, it is well-known that H_2_O_2_ is unstable and is prone to decomposition during the photocatalysis process, which, consequently, limits the H_2_O_2_ yield (SI Notes 1 and 2).^4^ To evaluate the photocatalytic decomposition behaviour, experiments were performed under identical conditions using N_2_-saturated solution containing 14.7 mmol per L H_2_O_2_ (Fig. 2c and S17). CGCN showed significantly higher H_2_O_2_ decomposition than pristine GCN, indicating that carbon doping promotes H_2_O_2_ degradation. In contrast, PHI exhibited the lowest decomposition activity, while PTI showed a behaviour similar to GCN. The PHI/PTI composite displayed intermediate decomposition activity, whereas Au deposition (1Au/PHI/PTI) further increased H_2_O_2_ decomposition compared to PHI/PTI, agreeing well with the previous reports.^22,24^ The kinetic plots of the photocatalytic H_2_O_2_ decomposition are shown in Fig. S18a. The decomposition rate *k*_d_ (Fig. S18b and Table S5) followed the subsequent order: CGCN (0.010 min^−1^) > PTI (0.0090 min^−1^) > Au/PHI/PTI (0.0089 min^−1^) > GCN (0.0081 min^−1^) > PHI/PTI (0.0067 min^−1^) > PHI (0.0048 min^−1^). These results contribute to explaining why the PHI + PTI physical mixture exhibits lower photocatalytic activity than pristine PHI (Fig. S14). Indeed, once H_2_O_2_ is generated, it is rapidly decomposed in the presence of PTI, thereby reducing its overall accumulation and the apparent photocatalytic performance. This phenomenon is suppressed in the PHI/PTI system and is associated with the formation of a heterojunction within this system.

To obtain more information on the electron–hole recombination, PL and PLQY spectroscopy were performed.^69^Fig. 2e and S19 show the PL emission spectra of GCN, CGCN, PHI, PTI, PHI/PTI, and 1Au/PHI/PTI photocatalysts under 360 nm excitation. As expected, GCN shows the highest PLQY (0.041), confirming its highest charge recombination. The PLQY values of the other photocatalysts follow the subsequent order: GCN(0.041) > CGCN (0.018) > PTI (0.014) > PHI/PTI (0.012) > PHI (0.010) > 1Au/PHI/PTI (0.009). Moreover, Au deposition blueshifted the PL emission peak from 517 nm in PHI/PTI to 484 nm, suggesting that Au alters the electron-transfer pathway and thereby influences the radiative electron–hole recombination.^70^

This suggests that additional factors influence H_2_O_2_ productivity in these photocatalysts. Since photocatalytic oxygen reduction can occur *via* different pathways, including direct two-electron transfer to H_2_O_2_ and direct four-electron transfer to H_2_O, a series of LSV was conducted under dark conditions to probe the number of transferred electrons (Fig. S20).

Subsequently, the electron transfer kinetic was calculated *via* the Koutecky–Levich (K–L) plots^27^ (Fig. 2f and S21). GCN and CGCN exhibit a similar number of electron transfers, suggesting that the carbon-doping strategy does not influence the selectivity. Both two- and four-electron transfer pathways occur, but the two-electron transfer pathway is more favoured. PHI, PHI/PTI, Au/PHI/PTI favour the two-electron transfer process to H_2_O_2_. However, PTI is more selective for the four-electron pathway to H_2_O than for the two-electron pathway to H_2_O_2_. Thus, the trace amount of H_2_O_2_ produced by PTI is likely attributable to a minor two-electron transfer process, despite its strong light absorption and large surface area.

### 3.3. Charge transfer mechanism and photocatalytic H_2_O_2_ production mechanism

**Fig. 3:**
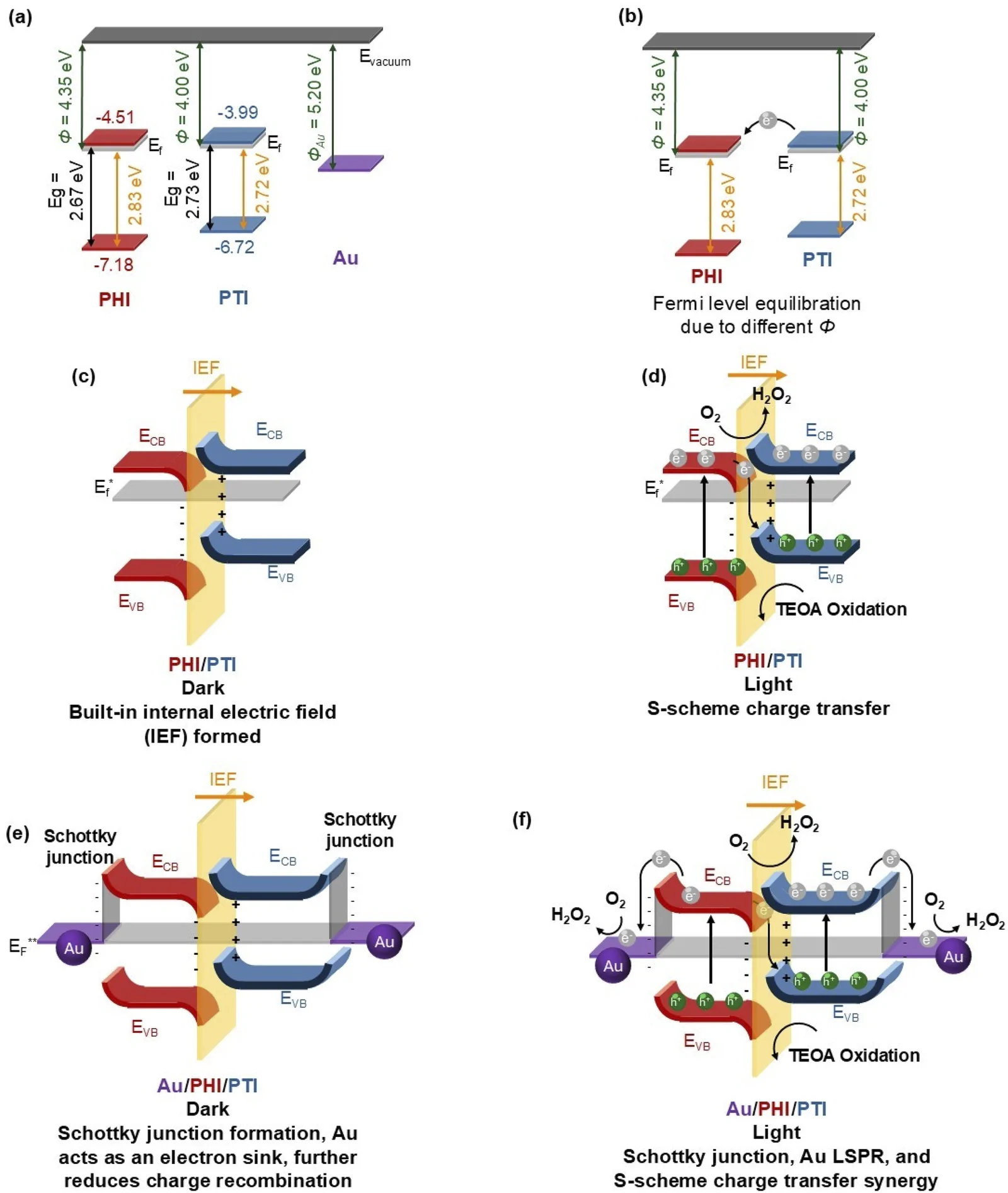
(a) Calculated band potentials of PHI, and PTI, compared to Au. (b) Proposed electron transfer between PHI and PTI in PHI/PTI heterojunction upon being in contact. (c) Proposed band structure of PHI/PTI in the dark and (d) charge transfer under light irradiation following the S-scheme mechanism. (e) Proposed electron transfer between PHI/PTI heterojunction and gold upon being in contact in the dark and (f) charge transfer under light irradiation.

The charge transfer mechanism is then proposed based on the band potentials obtained from UPS and solid-state UV-Vis spectroscopy (Fig. 3, S22, S23 and Table S6). It can be seen that PTI has a smaller work function (4.00 eV *vs.* vacuum) and a more positive Fermi level than PHI (4.35 eV *vs.* vacuum), Fig. 3a. Therefore, upon contact, electrons will transfer from PTI to PHI to equilibrate the different Fermi levels (Fig. 3b). As a result, the electrons will accumulate in PHI, and holes will remain in PTI, leading to an upward and downward band bending at the PHI and PTI interface side, respectively (Fig. 3c). The accumulation of electrons and holes on opposite sides of the junction generates an *in situ* electric field (IEF). Under light irradiation (Fig. 3d), photogenerated electrons and holes are generated in both PHI and PTI. This IEF drives the photogenerated electrons in the conduction band of PHI to recombine with the photogenerated holes in PTI, *via* an S-scheme charge-transfer mechanism.^71,72^ As a result, electron–hole recombination can be reduced, improving the photocatalytic activity compared to either PHI or PTI component alone.

The observed enhancement in H_2_O_2_ formation indicates that charge carriers are likely redistributed at the PHI/PTI interface, forming delocalised electrons at the contact interface that facilitate the reduction of oxygen into H_2_O_2_.

In the Au/PHI/PTI system, there is an obvious shift in the VB onset of 1Au/PHI/PTI (2.71 eV) relative to PHI/PTI (2.56 eV), indicating band bending at the Au/PHI/PTI interface and further supporting Schottky barrier formation. It is well established that Au can act as an electron sink due to its significantly higher work function (5.2 eV *vs.* vacuum) compared to most n-type semiconductors,^73,74^ including PHI (4.35), PTI (4), PHI/PTI (3.74). Contact between Au and PHI/PTI will equilibrate the previously equilibrated Fermi level of PHI/PTI by transferring electrons from PHI/PTI to Au, driven by differences in the work functions (and Fermi levels) of PHI/PTI and Au (Fig. 3e). As a result, band bending will occur at the PHI/PTI/Au contact interface and simultaneously form a Schottky barrier (Fig. 3e). Under light irradiation (Fig. 3f), photogenerated electrons in PHI and PTI can transfer to Au due to the electron sink effect of Au.^75^ Moreover, the back-transfer of photogenerated electrons from Au to PHI and PTI is prevented by the Schottky barrier, therefore, reducing the electron–hole recombination, as evidenced by the lowest PLQY of 1Au/PHI/PTI compared to other samples.^76^ In addition, *in situ* XPS measurements under illumination (Fig. S24) show a further negative shift (*ca.* 0.4 eV) of the Au 4f peaks, demonstrating the accumulation of photogenerated electrons on the Au nanoparticles under irradiation. These observations support the role of Au as an electron sink, facilitating charge separation and promoting the oxygen reduction reaction.

In the aim of identifying the reactive oxygen species (ROS) formed during the O_2_ reduction to H_2_O_2_ over the 1Au/PHI/PTI photocatalyst under UV-Vis irradiation, EPR experiment, with DMPO as the spin-trap, was carried out. Fig. S25 shows the hyperfine signal assigned to the DMPO-superoxide radical (O_2_˙^−^) adduct, which can be verified by the simulated spectrum (*A*_N_ = 13.9 G, *A*_H_ = 9.8 G, and *A*_Hγ_ = 1.6 G, *g* = 2.03515)^77,78^ obtained by MATHLAB Software and the EasySpin package. It should be noted that the intermediate is more likely to be O_2_˙^−^ than hydroperoxyl radical (HOO˙) due to the neutral pH of the reaction solution and the p*K*_a_ of HOO˙/O_2_˙^−^ couple is 4.8.^79^ The formation of O_2_˙^−^ is through one-electron transfer to O_2_ under light irradiation. Subsequently, the formed O_2_˙^−^ undergoes another one-electron reduction reaction in the presence of H^+^ to form H_2_O_2_. The involvement of O_2_˙^−^ was further verified by radical scavenging experiments (Fig. S26). The addition of benzoquinone (BQ), an O_2_˙^−^ scavenger, significantly suppressed H_2_O_2_ production, confirming O_2_˙^−^ as the key intermediate in the reaction. In contrast, the negligible change observed after adding *tert*-butanol (*t*-BuOH) excluded ˙OH as a major contributor. The addition of NaN_3_ as a ^1^O_2_ quencher resulted in an approximately 15% decrease in H_2_O_2_ yield, indicating that ^1^O_2_ also participate in the reaction. However, the much stronger inhibition caused by O_2_˙^−^ scavenging demonstrates that the O_2_˙^−^-mediated pathway dominates the photocatalytic H_2_O_2_ production process, while ^1^O_2_ plays a secondary role. Based on the discussion, the charge transfer mechanism for the photocatalytic oxygen reduction to H_2_O_2_ is proposed in Fig. 3f.

### 3.4. Reaction conditions optimisation and reusability assessment

**Fig. 4:**
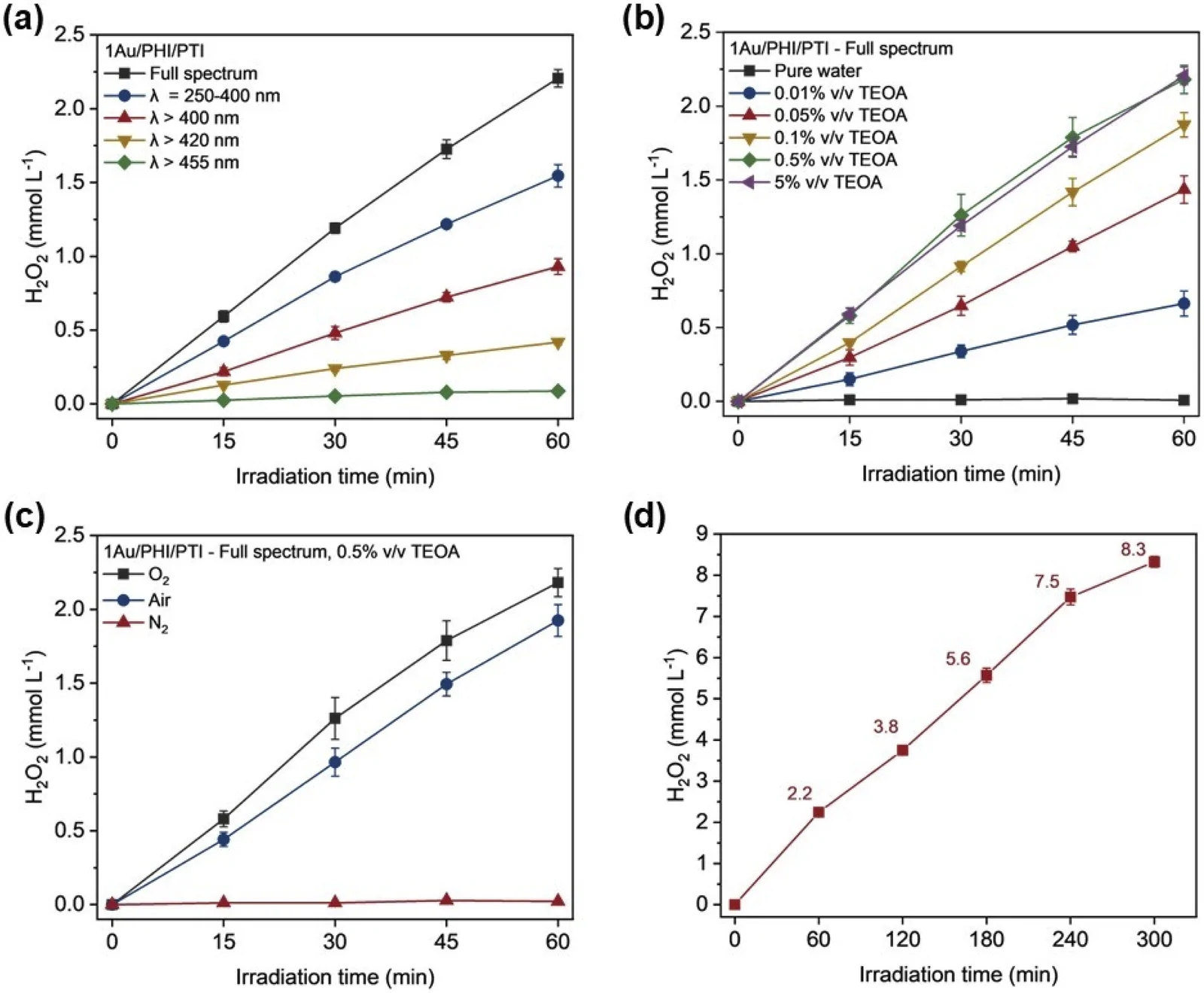
Effects of (a) light irradiation regions, (b) TEOA concentration, and (c) reaction atmospheres on photocatalytic H_2_O_2_ production by 1Au/PHI/PTI. (d) Continuous H_2_O_2_ production under optimised conditions (catalyst 8 mg, 0.05 v/v% TEOA/H_2_O 80 mL, full-spectrum, O_2_-saturated).

1Au/PHI/PTI was selected for further investigation and optimisation due to its highest activity under full-spectrum irradiation. The effect of the irradiation wavelength (Fig. 4a) was investigated using different long-pass filters (Fig. S27). The highest H_2_O_2_ yield (2.21 ± 0.06 mmol L^−1^) was obtained under full-spectrum irradiation. Limiting irradiation to the UV region (250–400 nm) reduced the yield to 1.54 ± 0.08 mmol L^−1^, while visible-NIR irradiation (*λ* > 400 nm) produced 0.93 ± 0.05 mmol L^−1^. Further increasing the cut-off wavelength to *λ* > 420 nm decreased the yield to 0.42 ± 0.02 mmol L^−1^, reflecting the lower photon energy available for electron excitation that is responsible for the photocatalytic activity. Only trace H_2_O_2_ (0.09 ± 0.01 mmol L^−1^) was produced at *λ* > 450 nm, further supporting that excitation of PHI/PTI is necessary to initiate the photocatalysis despite the LSPR absorption of Au. The influence of the sacrificial agent was also investigated (Fig. 4b). Without TEOA, only trace H_2_O_2_ was detected, confirming its essential role as a hole scavenger. Reducing the TEOA concentration from 5 to 0.5 vol% maintained a similar H_2_O_2_ yield (*ca.* 2.2 mmol L^−1^), while further decreases progressively reduced the yield. Therefore, 0.5 vol% TEOA was selected for subsequent experiments. It should be noted that a low concentration of H_2_O_2_ was detected when pure water was used (0.01 mmol L^−1^). The effect of oxygen concentration was examined under O_2_-, air-, and N_2_-saturated conditions (Fig. 4c). The H_2_O_2_ yield decreased slightly under air saturation (1.92 ± 0.11 mmol L^−1^) compared with O_2_ saturation (2.21 ± 0.06 mmol L^−1^), while no H_2_O_2_ was detected under N_2_, suggesting that H_2_O_2_ is produced *via* oxygen reduction reaction. The significant activity under air suggests good potential for practical applications. With the optimum condition *i.e.*, 0.5 v/v% TEOA, full-spectrum irradiation, O_2_-saturated solution, an excellent AQY of 18.8% was obtained. The H_2_O_2_ productivity and AQY exceed those reported for most photocatalytic systems (Fig. S28 and Table S7).

Moreover, the fate of TEOA during photocatalysis, beyond its role as a sacrificial agent, was investigated further using time-resolved ^1^H and ^13^C NMR analyses of the reaction solution (Fig. S29 and S30). The results reveal that TEOA undergoes oxidative dealkylation, producing diethanolamine (DEA), glycolaldehyde, glycolate, and formate (Fig. S31). The formation of DEA was confirmed by the appearance of characteristic NMR signals, while the oxidation products gradually increased with irradiation time. This transformation is consistent with the reported oxidative dealkylation pathway of tertiary alkanolamines.^80^ These results indicate that TEOA is not simply consumed as a sacrificial reagent but can be converted into value-added products, including DEA, which is an industrially relevant chemical used in gas treatment^81^ and personal-care formulations.^82^

The reusability of the 1Au/PHI/PTI photocatalyst was evaluated under optimised conditions. As shown in Fig. S32, the H_2_O_2_ yield decreased by ∼30% in the second cycle (from 2.21 ± 0.06 to 1.49 ± 0.10 mmol L^−1^) but remained relatively stable in the third cycle (1.44 ± 0.05 mmol L^−1^). Notably, the activity in both cycles remained higher than that of bare PHI/PTI. ICP-MS analysis revealed that the Au content decreased from 0.65 wt% to 0.46 wt% and to 0.44 wt% after the first and second cycles, and stabilised at ∼0.41 wt% after the third cycle, indicating that Au leaching is the main cause of activity loss. To avoid catalyst loss during recovery steps, the reaction was run continuously without intermediary recovery/recycling stages. After 3 h irradiation (Fig. 4d), 5.57 ± 0.17 mmol L^−1^ of H_2_O_2_ was produced, exceeding the cumulative yield from three 1 h cycles (5.11 mmol L^−1^). After 5 h, the H_2_O_2_ concentration reached 8.32 ± 0.13 mmol L^−1^, lower than the theoretical value, likely due to Au leaching, decreased dissolved O_2_ concentration, and partial H_2_O_2_ decomposition. ICP-MS analysis (Fig. S33) showed that most of the Au leaching occurred within the first hour (1.64 ± 0.01 mg, 50% of the initial loading), with minimal additional loss afterwards, confirming that early-stage metal loss contributes to the decline in photocatalytic performance. The leaching is likely due to the weak interaction of Au with the support, as discussed above. Overall, although partial Au leaching occurs during the reaction, the catalyst maintains a relatively high activity and remains more active than the bare PHI/PTI.

Post-reaction characterisation was carried out to investigate the decline in the photocatalytic activity of 1Au/PHI/PTI and assess the extent of Au leaching. TEM analysis (Fig. S34) of recovered catalysts after cycling and prolonged irradiation revealed a reduction in the average Au nanoparticle size, with larger particles largely absent, confirming Au loss during the reaction due to weak interaction of large nanoparticles. The slightly larger average particle size observed after the 5 h continuous run, compared with the cycled samples, suggests that repeated washing and recovery steps may further promote the detachment of larger Au particles.

XPS analysis of fresh and used 1Au/PHI/PTI samples (after 3 of 1 h cycles and a single 5 h run) revealed distinct structural changes depending on reaction duration (Fig. S35 and Table S8). For the C 1s spectra (Fig. S35a), no obvious difference in binding energies of different C-species was observed. However, the sample collected after three cycles showed oxidation of the conjugated C–N_3_ framework (C–OH increasing from 14.2% to 19.5%, and aromatic sp^2^ C (C–N_3_) decreasing from 73.6% to 67.7%). This oxidation is likely due to repeated recovery and washing steps rather than intrinsic photocatalytic degradation. No obvious change in the composition was observed from the 5 h sample. Interestingly, the N 1s spectra (Fig. S35b) show the opposite trend to the C 1s spectra. The 5 h sample exhibited significant N hydrogenation (C–N–H increasing (2.9% to 8.2%), C=N–C decreasing (75.0% to 73.6%), and N–C_3_ decreasing (17.2% to 13.2%)), whilst no change was observed from the sample collected after three cycles. For both samples, no apparent changes were observed for Li, K, and Cl states (Fig. S35c–e). These opposite changes in C 1s and N 1s spectra of the recovered samples after three cycles and a single 5 hour run suggest structural changes, but in different ways: oxidation and hydrogenation of the frameworks, respectively. For the oxidation states of Au, the high-resolution Au 4f spectra of the fresh and the 3^rd^ cycle used sample (Fig. S35f) show a slight positive shift in the characteristic binding energies associated with metallic Au species, suggesting the oxidation of Au in the recovered sample after the 3^rd^ cycle. However, the Au 4f spectra of the sample used in the five-hour reaction show a dramatic change. The binding energy of Au shifts towards a less positive value (Au 4f_7/2_ from 83.7 to 83.4 eV), suggesting the formation of a more anionic Au. This shift in binding energy in the used sample suggests an electron enrichment of the gold nanoparticles, resulting from electron transfer from the PHI/PTI support to the Au nanoparticles. Overall, the loss in H_2_O_2_ productivity in these samples is mainly due to the loss of Au particles, resulting from weak interaction with the support and structural changes during the reaction and recovery steps. Nevertheless, these activity results demonstrate that the 1Au/PHI/PTI system is a promising photocatalyst for H_2_O_2_ production, and that further improvements in cocatalyst stability could enhance its long-term performance and practical applicability.

## 4. Conclusion

This work demonstrates that the photocatalytic performance of crystalline carbon nitride materials for H_2_O_2_ production is determined not only by light absorption and surface area, but also by electron-transfer selectivity, charge-carrier recombination, and H_2_O_2_ degradation behaviour. The contrasting electron-transfer numbers of PTI (4) and PHI (2) serve as a clear example of how structural differences at the molecular level can fundamentally redirect oxygen reduction reaction pathways, rendering conventional photocatalytic activity descriptors insufficient. Furthermore, this study highlights that synthesis yield is a practical parameter that must be considered alongside intrinsic activity when selecting materials for scale-up. In this regard, the PHI/PTI heterostructure appears as a good option for scale-up. The synergistic combination of S-scheme charge transfer of PHI/PTI heterojunction, Schottky barrier formation and plasmonic enhancement by Au in the Au/PHI/PTI system offers a viable design strategy for promoting H_2_O_2_ photoproduction. Under optimised reaction conditions, the apparent quantum yield of 18.8% was achieved. Besides, continuous H_2_O_2_ production over extended irradiation was found to be more effective than repeated recovery-reuse cycling. The generated H_2_O_2_ holds promise for downstream applications in water treatment and green oxidation chemistry.

## Conflicts of interest

The authors declare that they have no competing interests.

## Supplementary Material

TA-014-D6TA04477H-s001

## Data Availability

The data supporting the findings of this study are available within the article and its supplementary information (SI). Supplementary information: notes, figures (Fig. S1–S35), tables (Tables S1–S8), additional materials characterization data (XRD, XPS, TEM, BET, NMR, and EPR), together with kinetic, electrochemical, and mechanistic results supporting the findings of this study. See DOI: https://doi.org/10.1039/d6ta04477h.
