# Supplementary material for: Au LSPR-boosted S-scheme carbon nitride heterostructures for H2O2 photocatalytic production
Source: J Mater Chem A Mater. 2026 Aug 6;14(53):36695–710. doi: 10.1039/d6ta04477h (PMC13551572; doi:10.1039/d6ta04477h)
Supplement: Supplementary file 1 [file TA-014-D6TA04477H-s001.pdf]

Supplementary Information (SI) for Journal of Materials Chemistry A.
This journal is © The Royal Society of Chemistry 2026

Supporting information

Au LSPR-Boosted S-Scheme Carbon Nitride Heterostructures for H_2O_2 Photocatalytic Production.

Supplementary Note 1

Kinetic analysis using a zeroth-order model for H_2O_2 formation and a first-order model for H_2O_2 decomposition was applied to determine the rate constants by using the following equations:[1]

$$[H_2O_2] = \frac{k_f}{k_d}(1 - e^{(-k_d t)}) \quad \text{(Eq.1)}$$

$$\ln\left(\frac{C_0}{C_t}\right) = k_d t \quad \text{(Eq.2)}$$

Where k_f and k_d are the formation and decomposition rate constants, C_t and C_0 denote the H_2O_2 concentrations at time *t* and at the start of the decomposition experiment (14.7 mmol L^{-1}), respectively.

Supplementary Note 2

The relative ratio of PHI to PTI, can be semi-quantitatively calculated by XRD, according to previous report, as follows:[2],

$$R_H = \frac{kA_H}{kA_H + A_T}$$

$$R_T = \frac{kA_T}{kA_H + A_T}$$

where R_H and R_T are the weight compositions of PHI and PTI, respectively. A_H and A_T are the integrated intensities of the (002) and (100) XRD peaks of PHI and PTI, respectively. The constant k, with a value of 0.884, is a correction factor that accounts for differences in scattering efficiency or crystallographic structure between PHI and PTI, ensuring the calculated weight fractions are accurate. As a result, the amounts of PHI and PTI are 31.5 and 68.5 wt%, respectively.

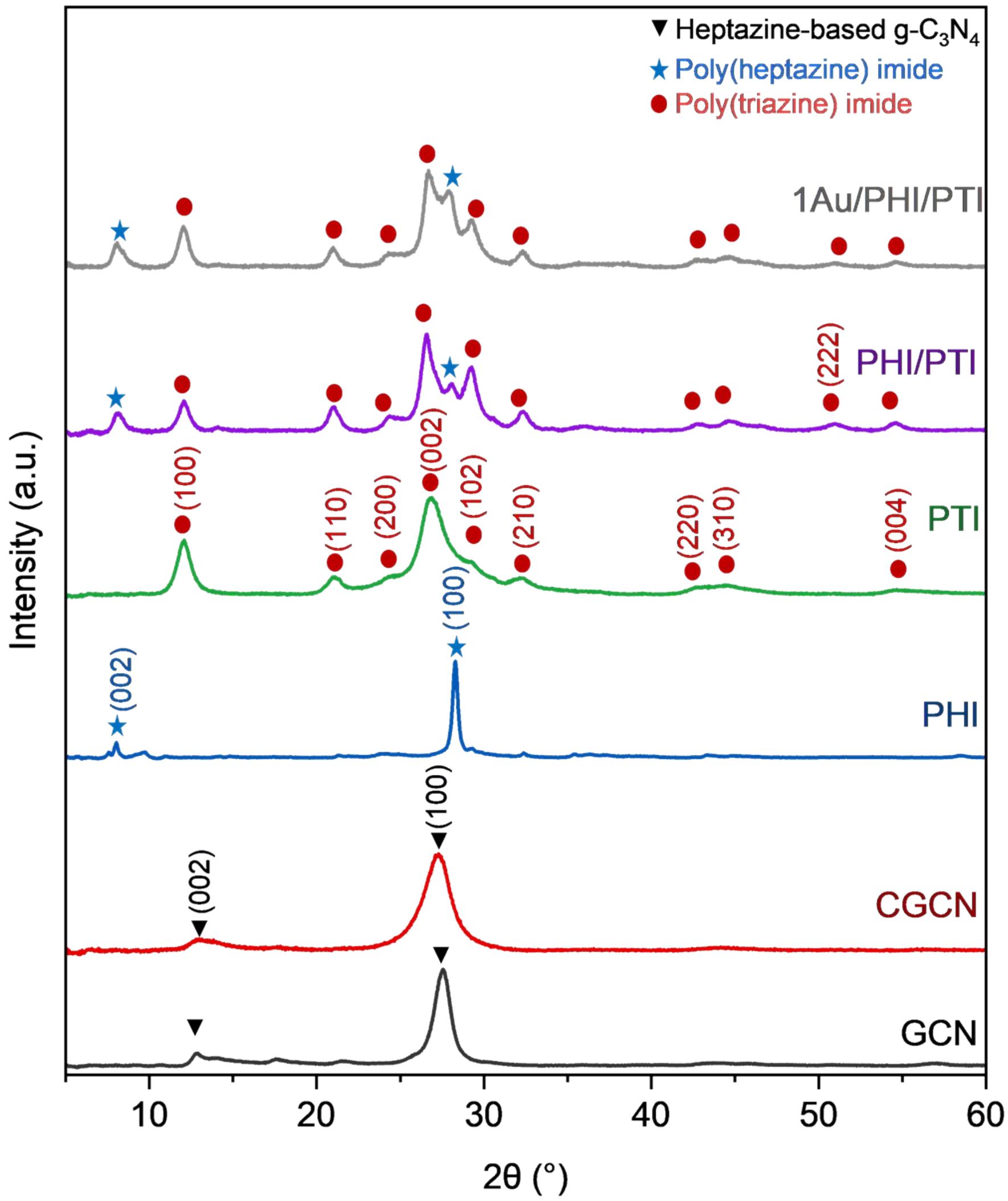

Figure S1. XRD patterns of GCN, CGCN, PHI, PTI, PHI/PTI, and 1Au/PHI/PTI samples.

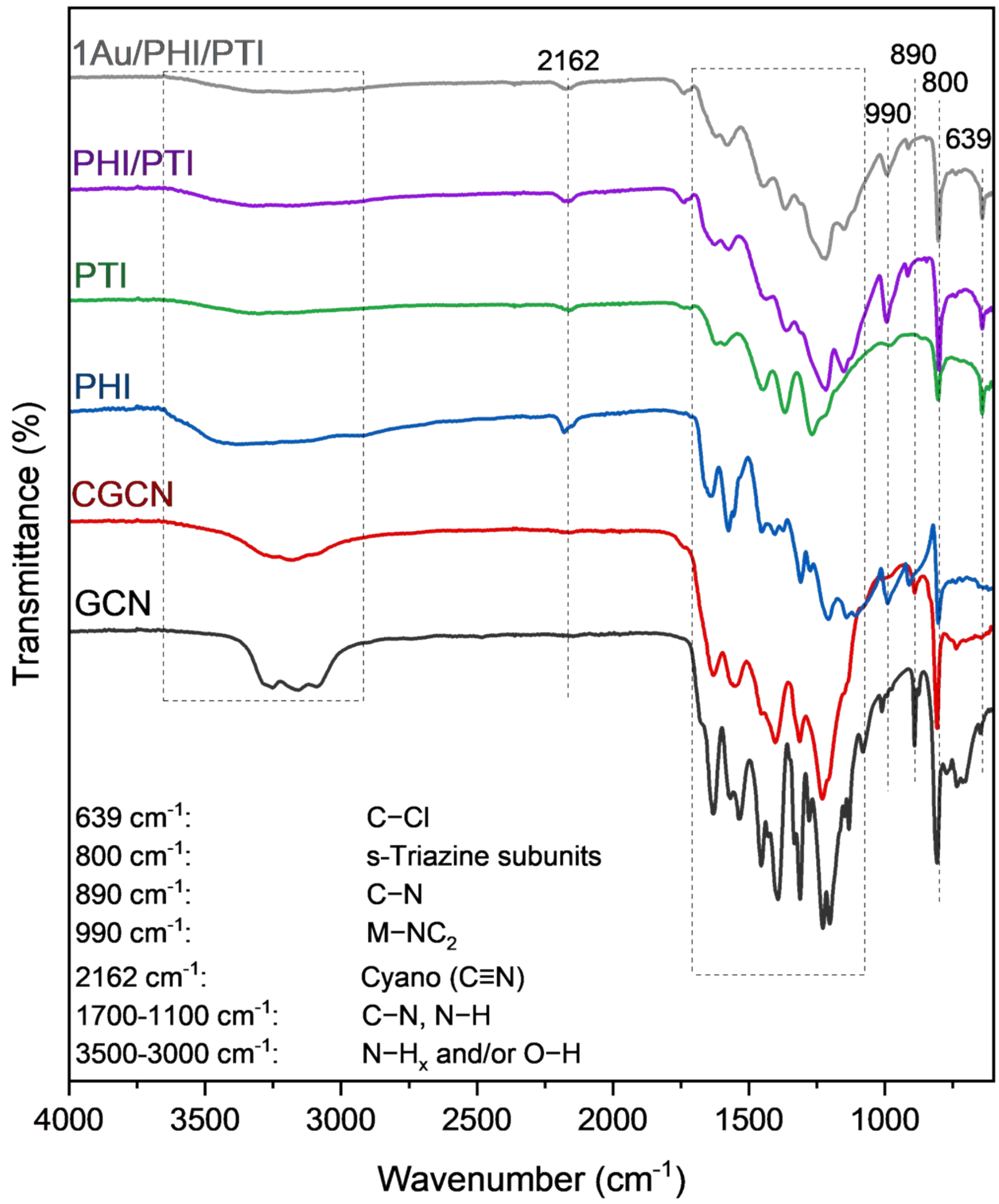

Figure S2. ATR-FTIR spectra of GCN, CGCN, PHI, PTI, PHI/PTI, and 1Au/PHI/PTI samples.

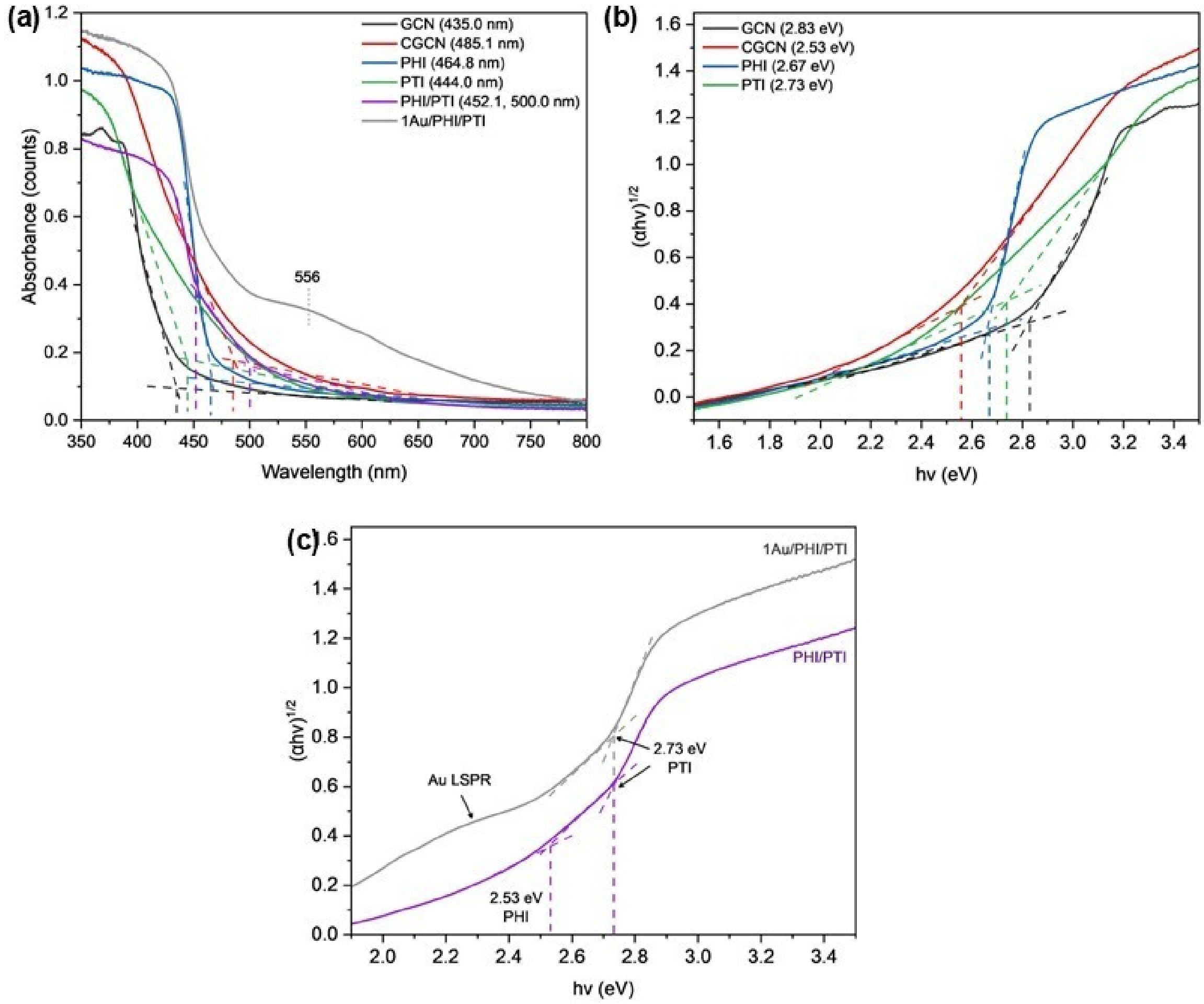

Figure S3. (a) UV-Vis DRS spectra of GCN, CGCN, GCN, PHI, PTI, PHI/PTI, and 1Au/PHI/PTI. Tauc's plots of (b) GCN, CGCN, GCN, PHI, and PTI, and (c) PHI/PTI and 1Au/PHI/PTI.

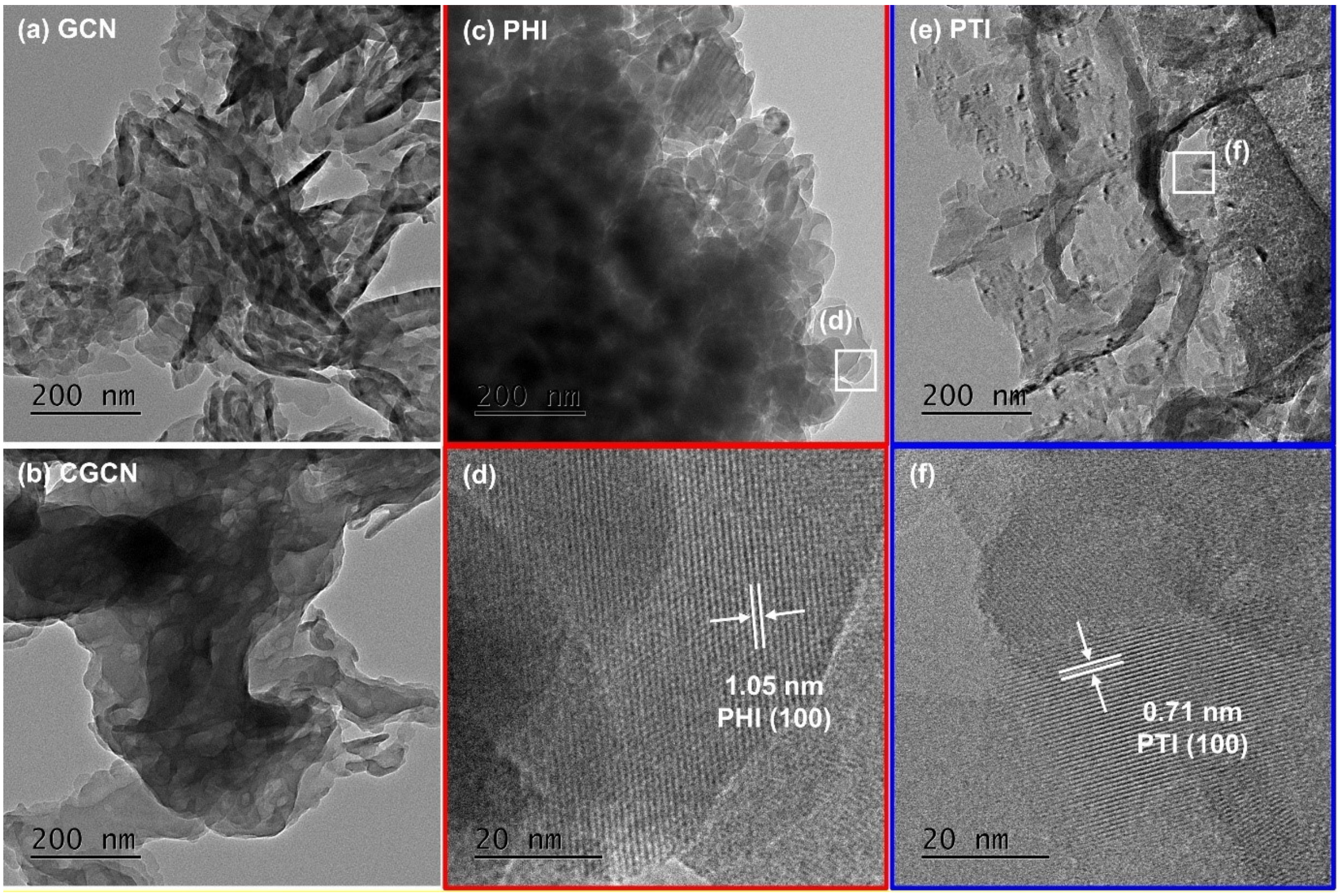

Figure S4. TEM images of (a) GCN, (b) CGCN, (c) PHI, (e) PTI, and high-magnification images of (d) PHI, and (f) PTI.

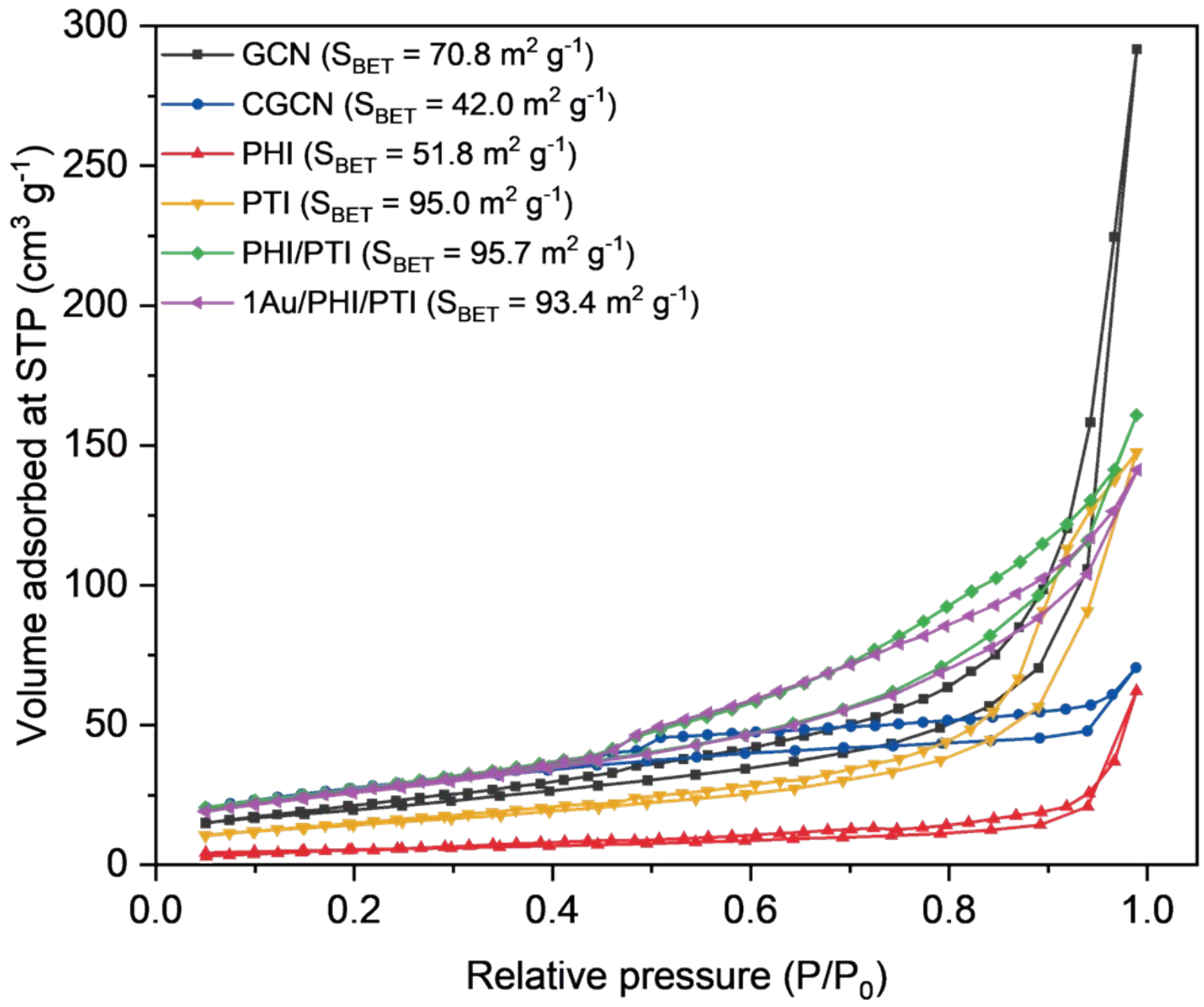

Figure S5. N_2 adsorption/desorption isotherms of GCN, CGCN, GCN, PHI, PTI, PHI/PTI, and 1Au/PHI/PTI.

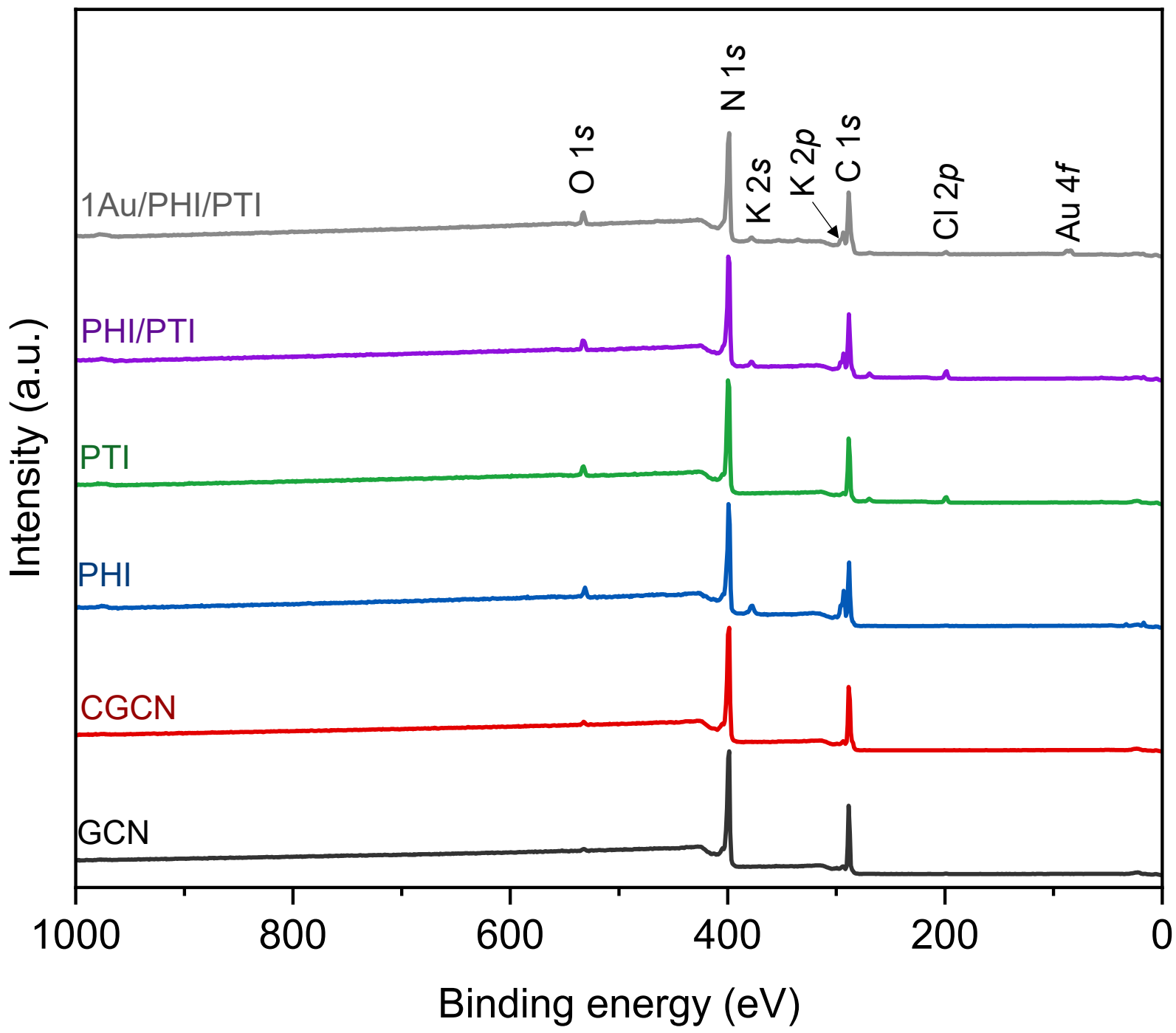

Figure S6. Survey XPS spectra of GCN, CGCN, GCN, PHI, PTI, PHI/PTI, and 1Au/PHI/PTI.

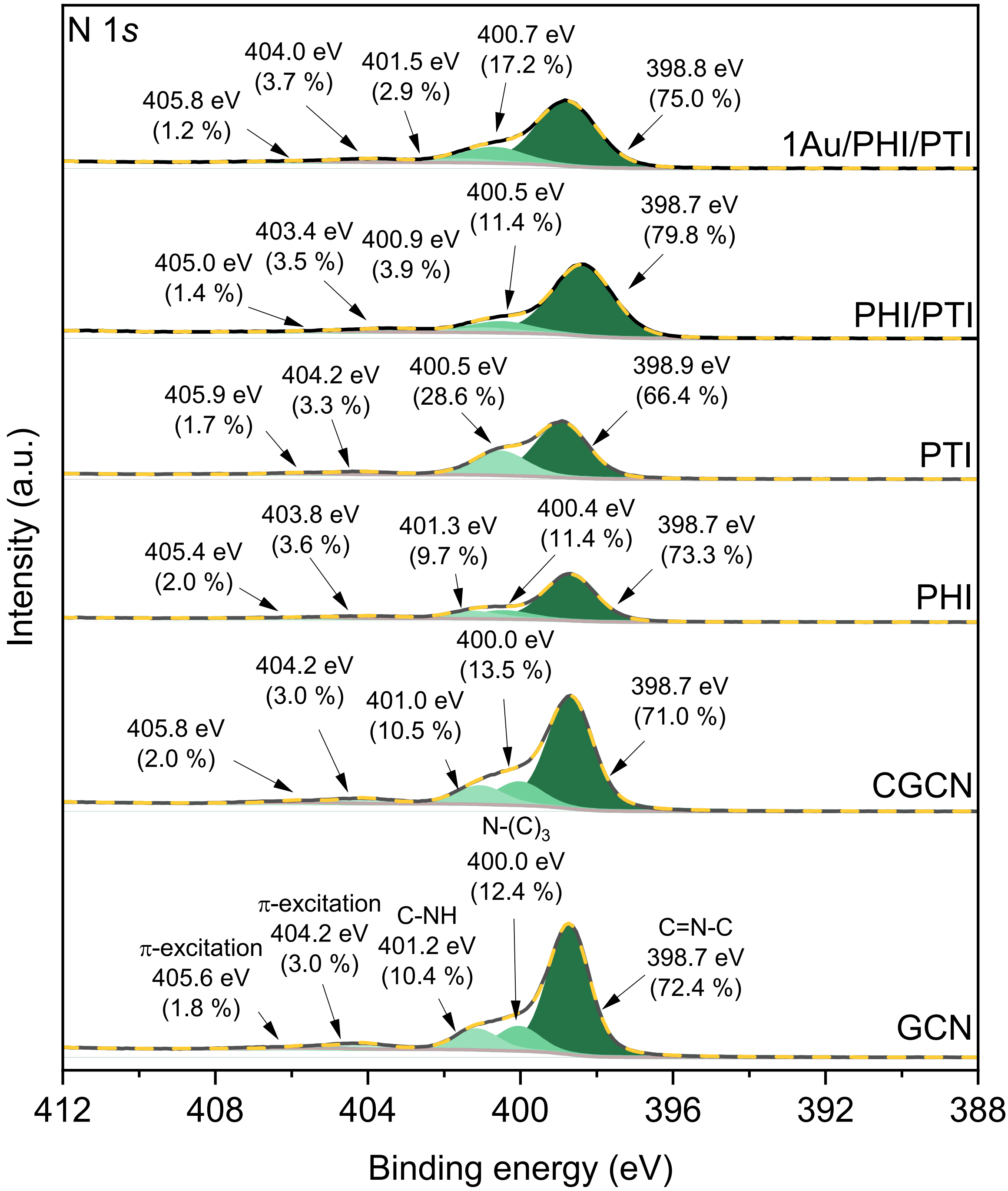

Figure S7. High-resolution N 1*s* XPS spectra of GCN, CGCN, PHI, PTI, PHI/PTI, and 1Au/PHI/PTI samples.

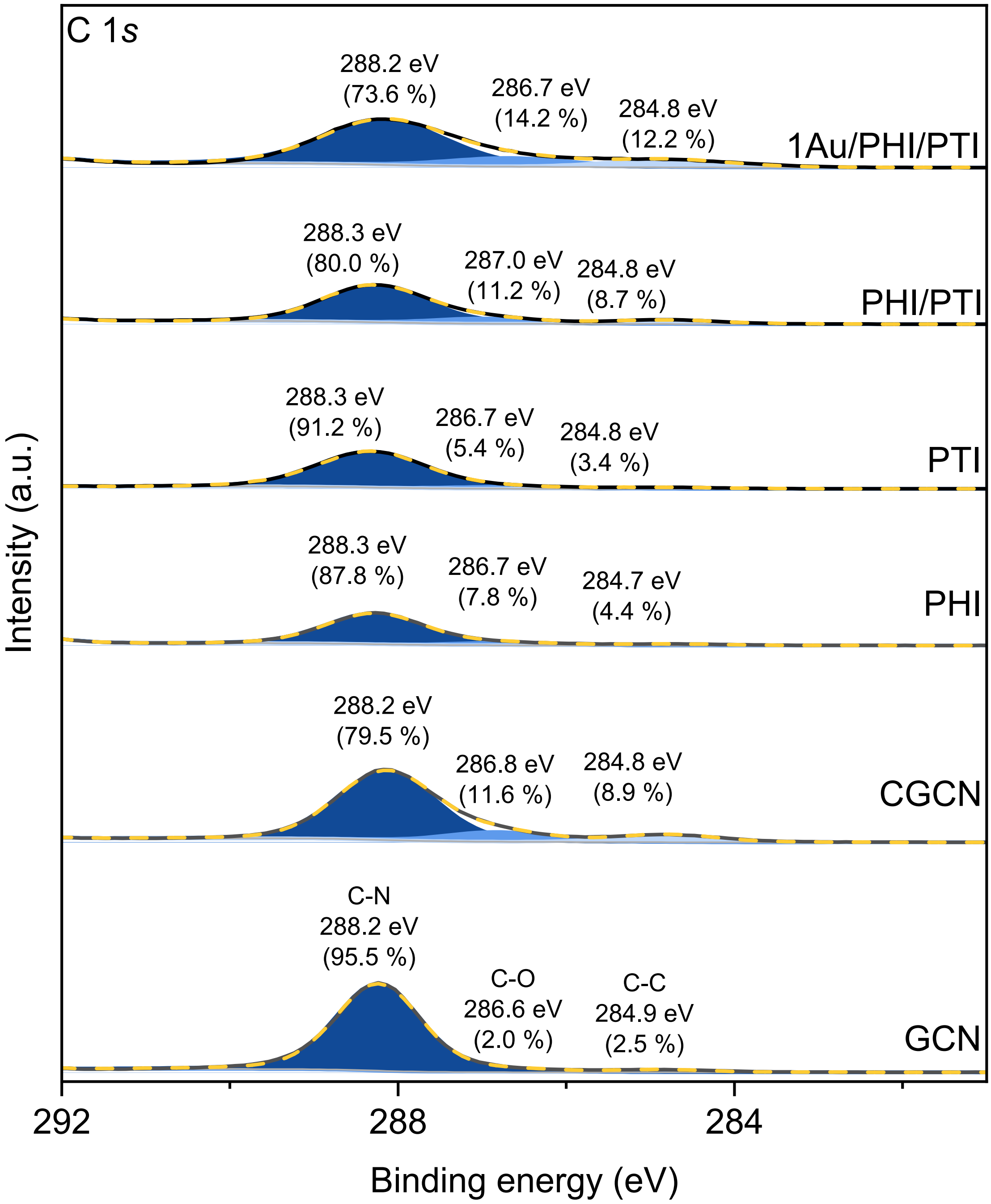

Figure S8. High-resolution C 1*s* XPS spectra of GCN, CGCN, PHI, PTI, PHI/PTI, and 1Au/PHI/PTI samples.

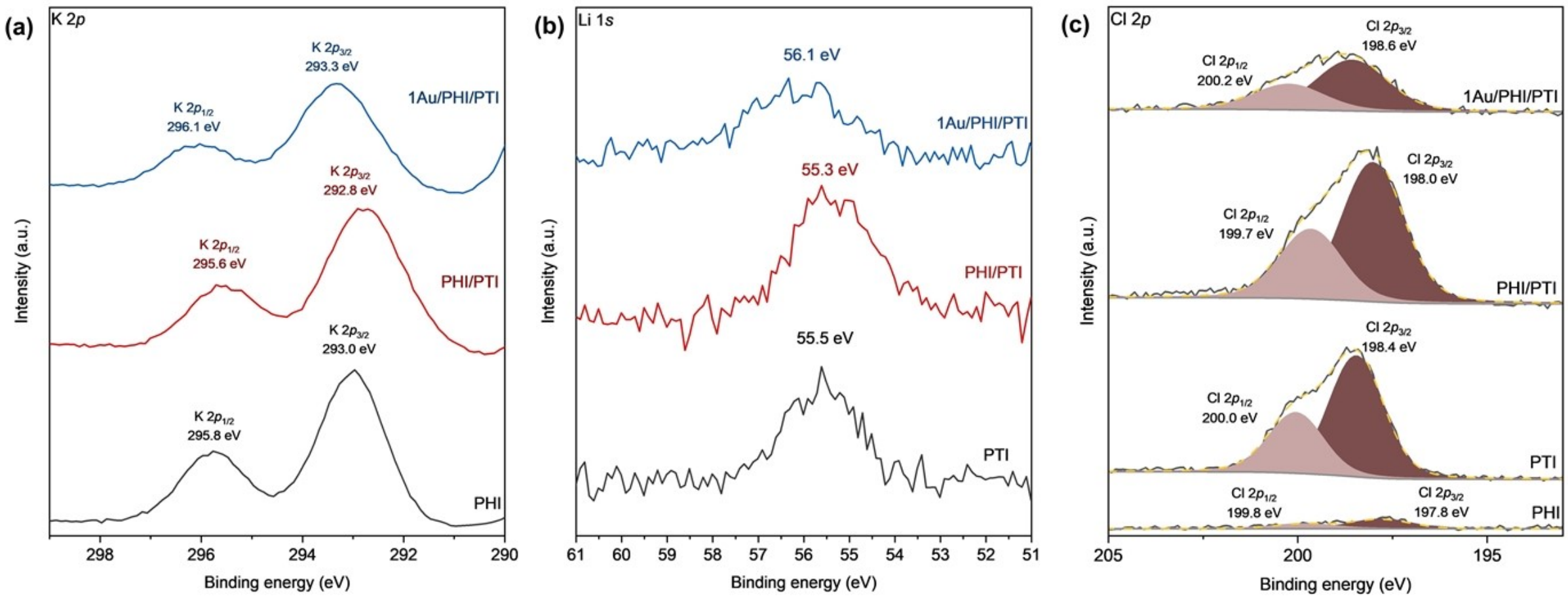

Figure S9. High-resolution (a) K 2*p*, (b) Li 1*s*, and (c) Cl 2*p* XPS spectra of synthesised photocatalysts.

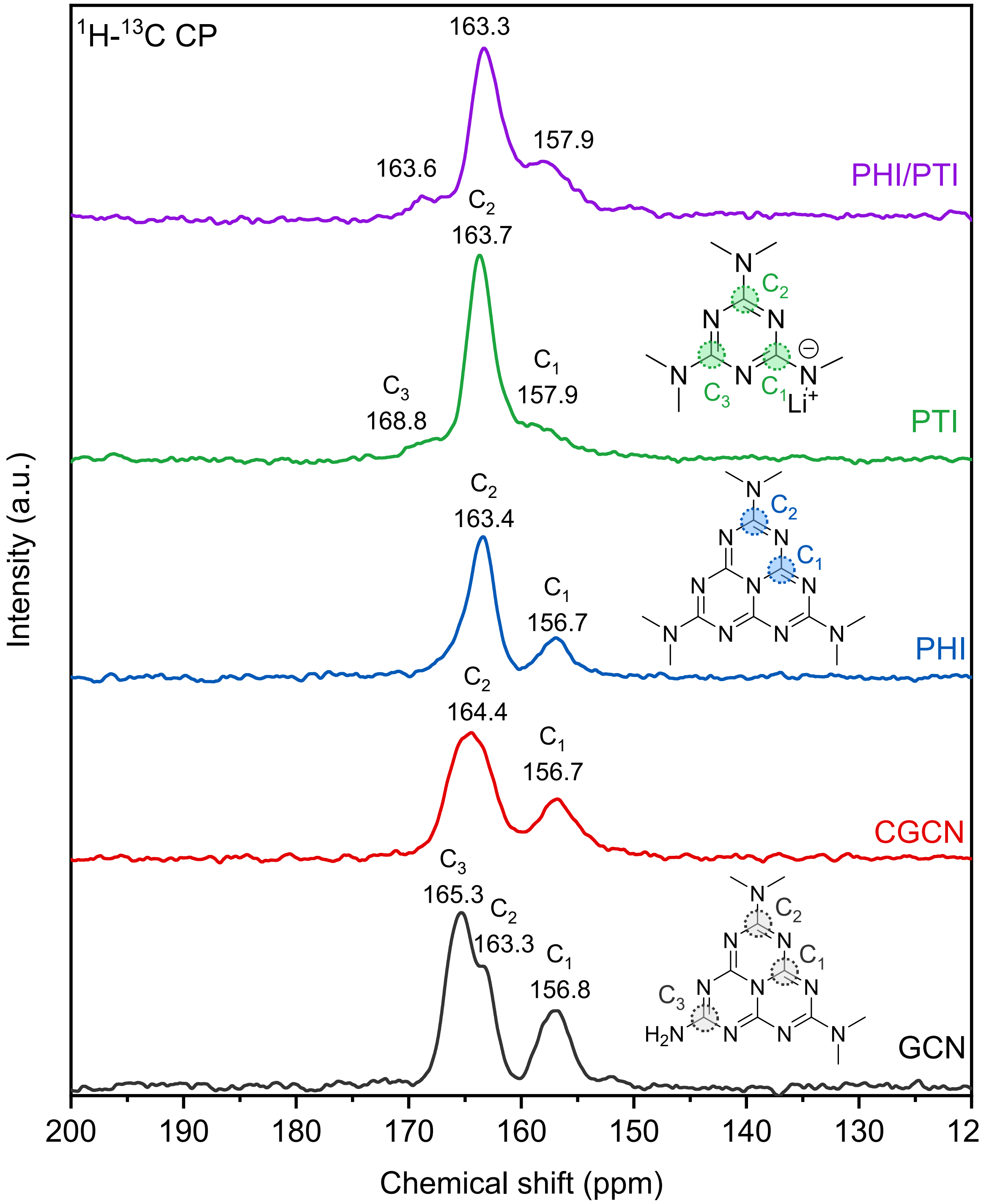

Figure S10. ^{13}C MAS NMR in a CP mode of the GCN, CGCN, PHI, PTI, and 1Au/PHI/PTI samples.

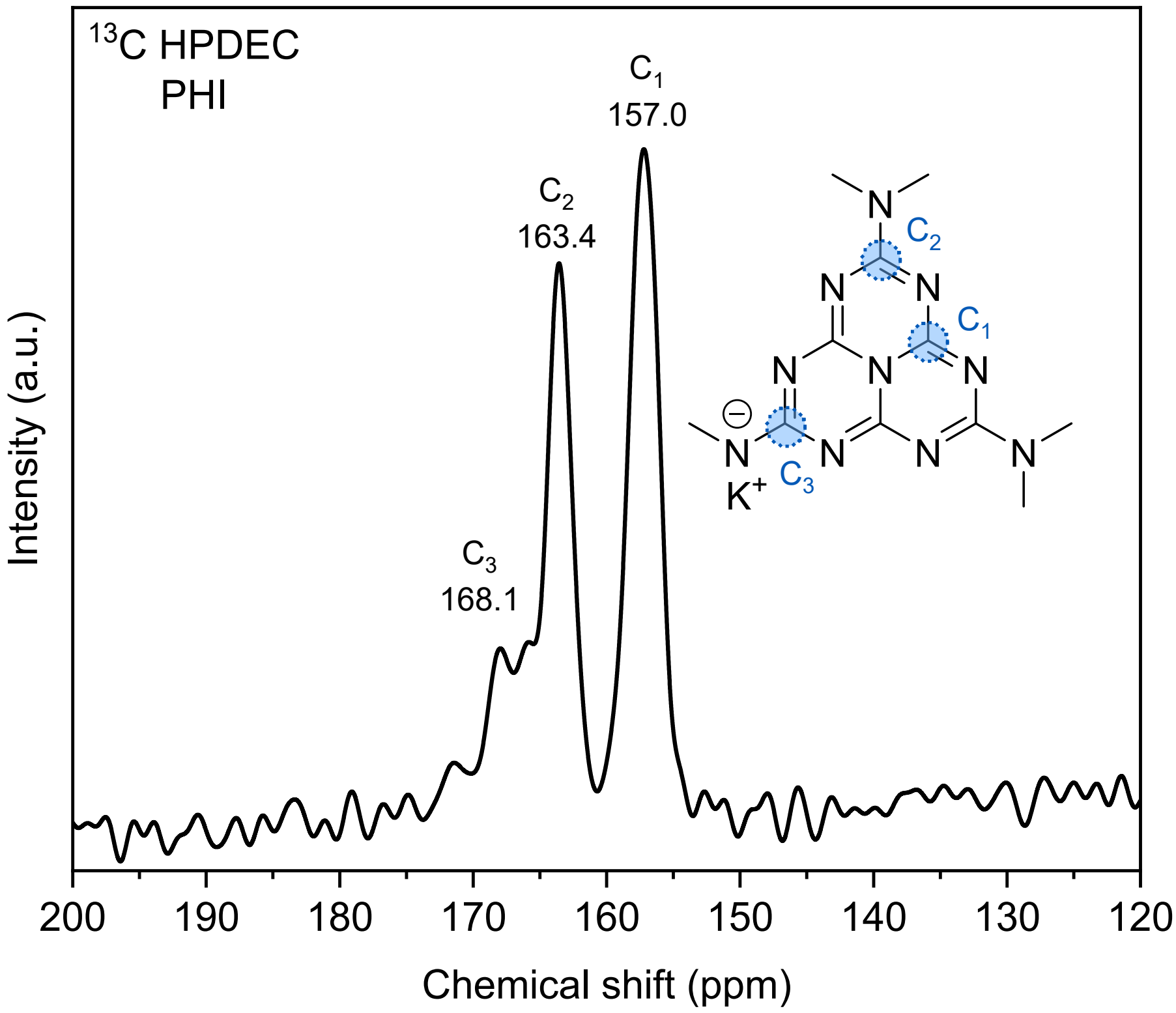

Figure S 11. ^{13}C MAS NMR in the HPDEC mode of the PHI sample.

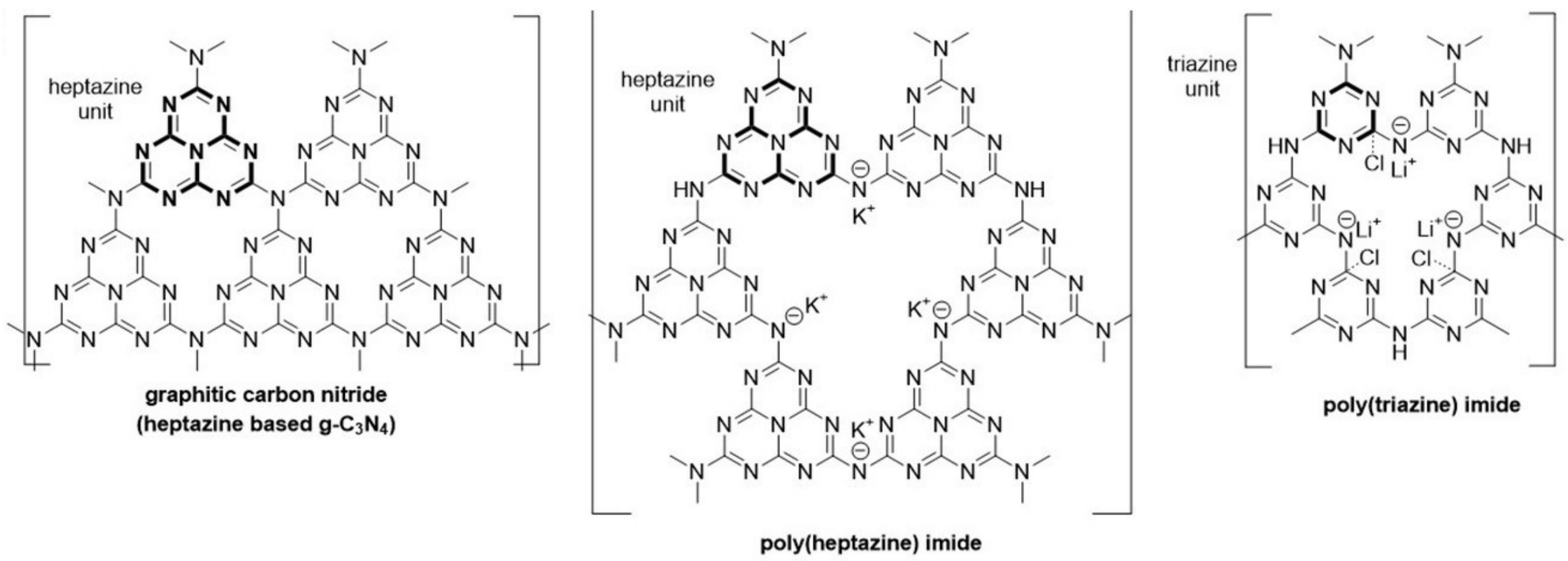

Figure S12. Illustrations showing the chemical structures of g-C_3N_4, PHI, and PTI.

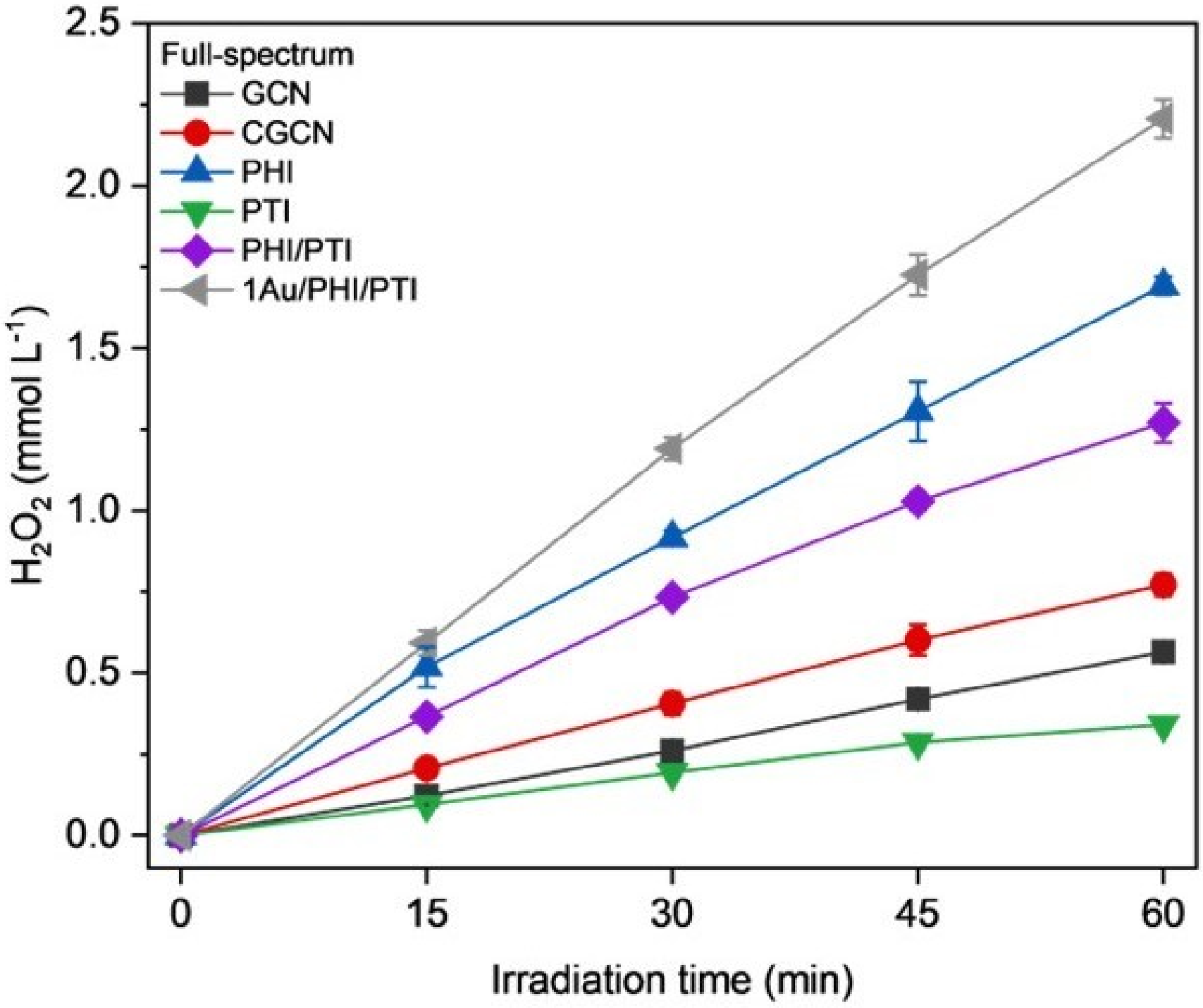

Figure S13. H_2O_2 production over GCN, CGCN, PHI, PTI, PHI/PTI, and 1Au/PHI/PTI under UV-visible-NIR. *Conditions: 8 mg catalyst, 80 mL 5 v/v% TEOA/H_2O, 300 W Xe lamp, O_2-saturated.*

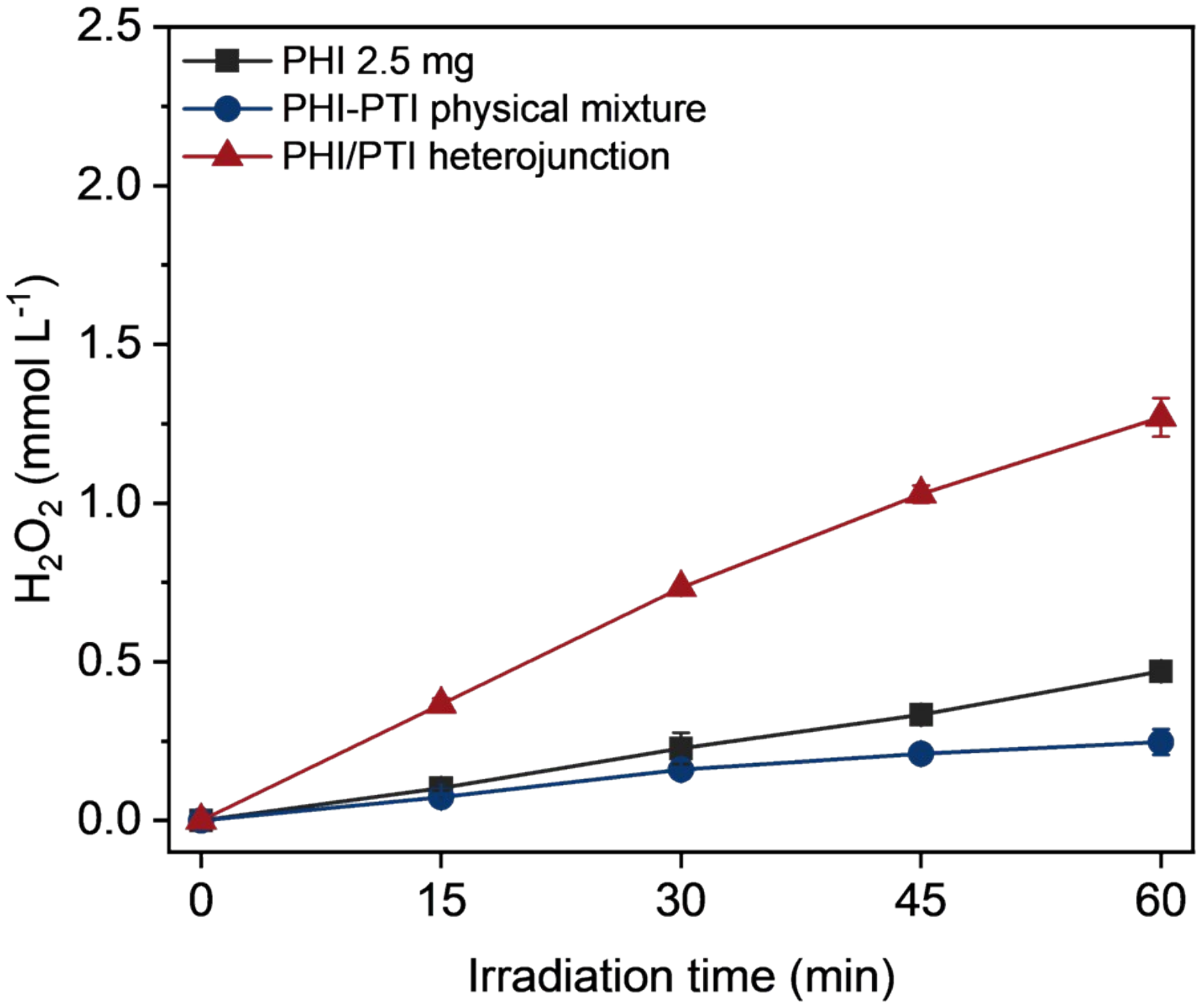

Figure S14. H_2O_2 production over PHI/PTI heterojunction, a physical PHI-PTI mixture, and PHI with the same loading (2.5 mg) as present in the PHI/PTI system.

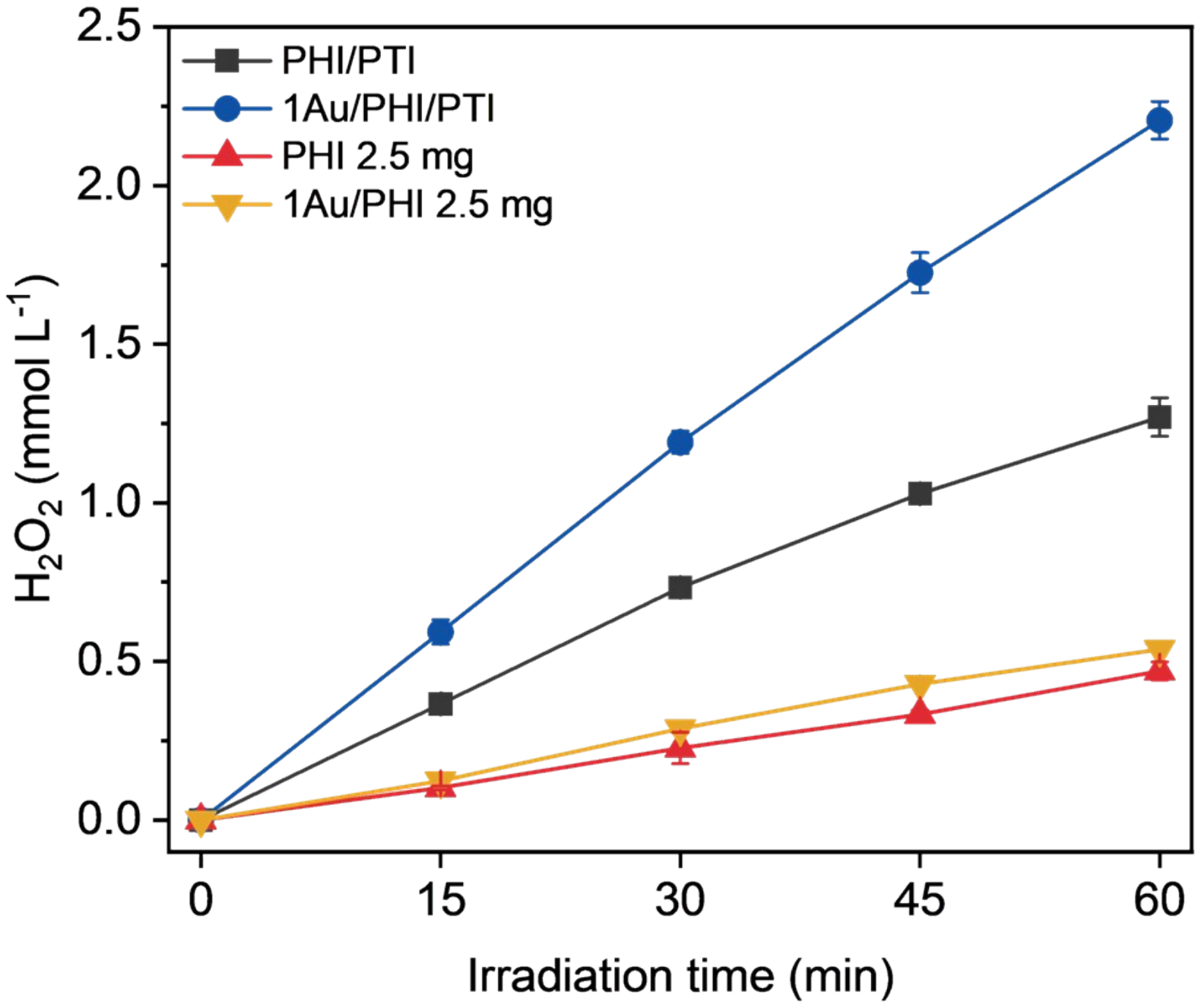

Figure S15. Effect of Au deposition on PHI and PHI/PTI for photocatalytic H_2O_2 production.

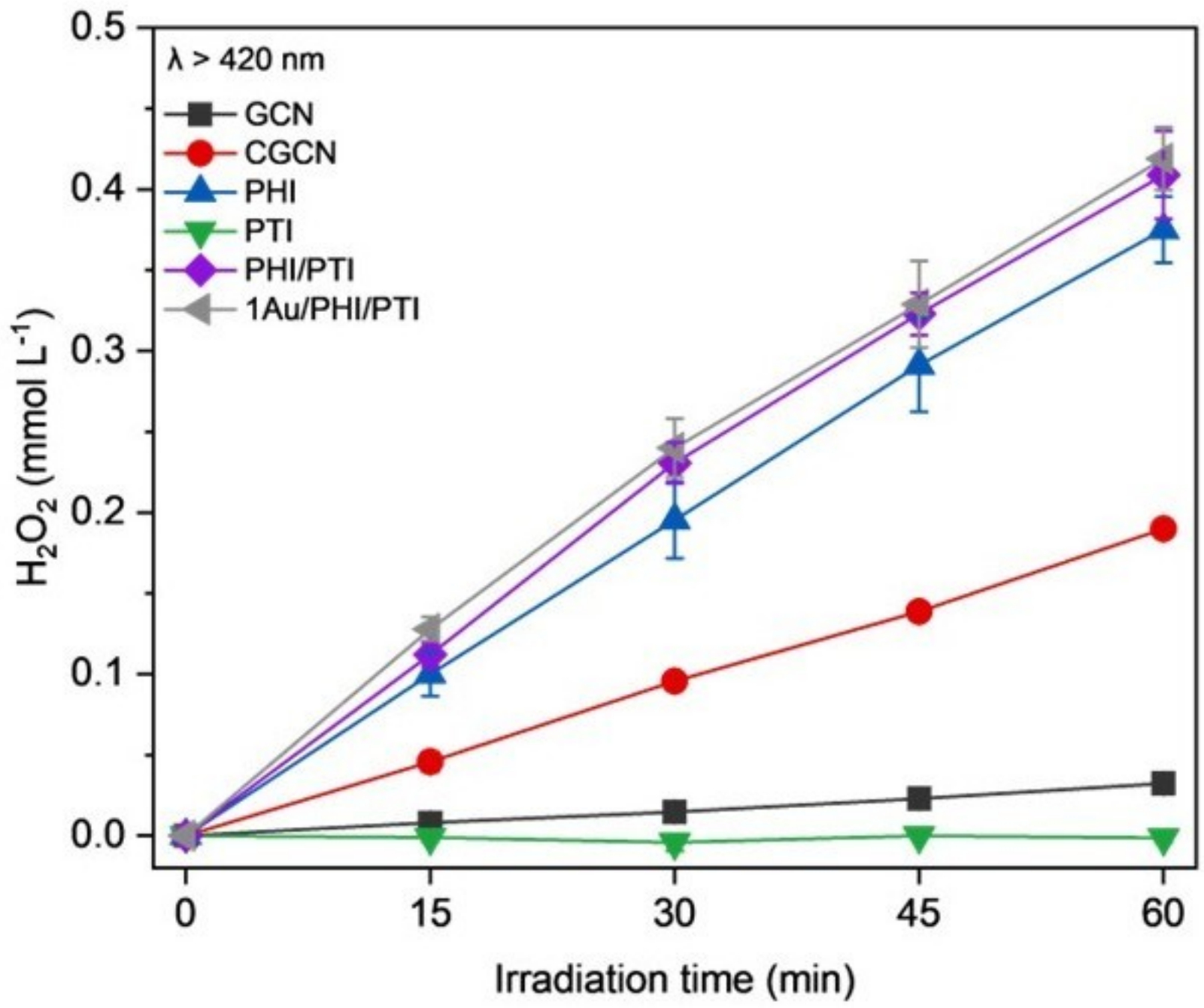

Figure S16. H_2O_2 production over GCN, CGCN, PHI, PTI, PHI/PTI, and 1Au/PHI/PTI under visible-NIR (λ > 420 nm) irradiation. *Conditions: 8 mg catalyst, 80 mL 5 v/v% TEOA/H_2O, 300 W Xe lamp, O_2-saturated.*

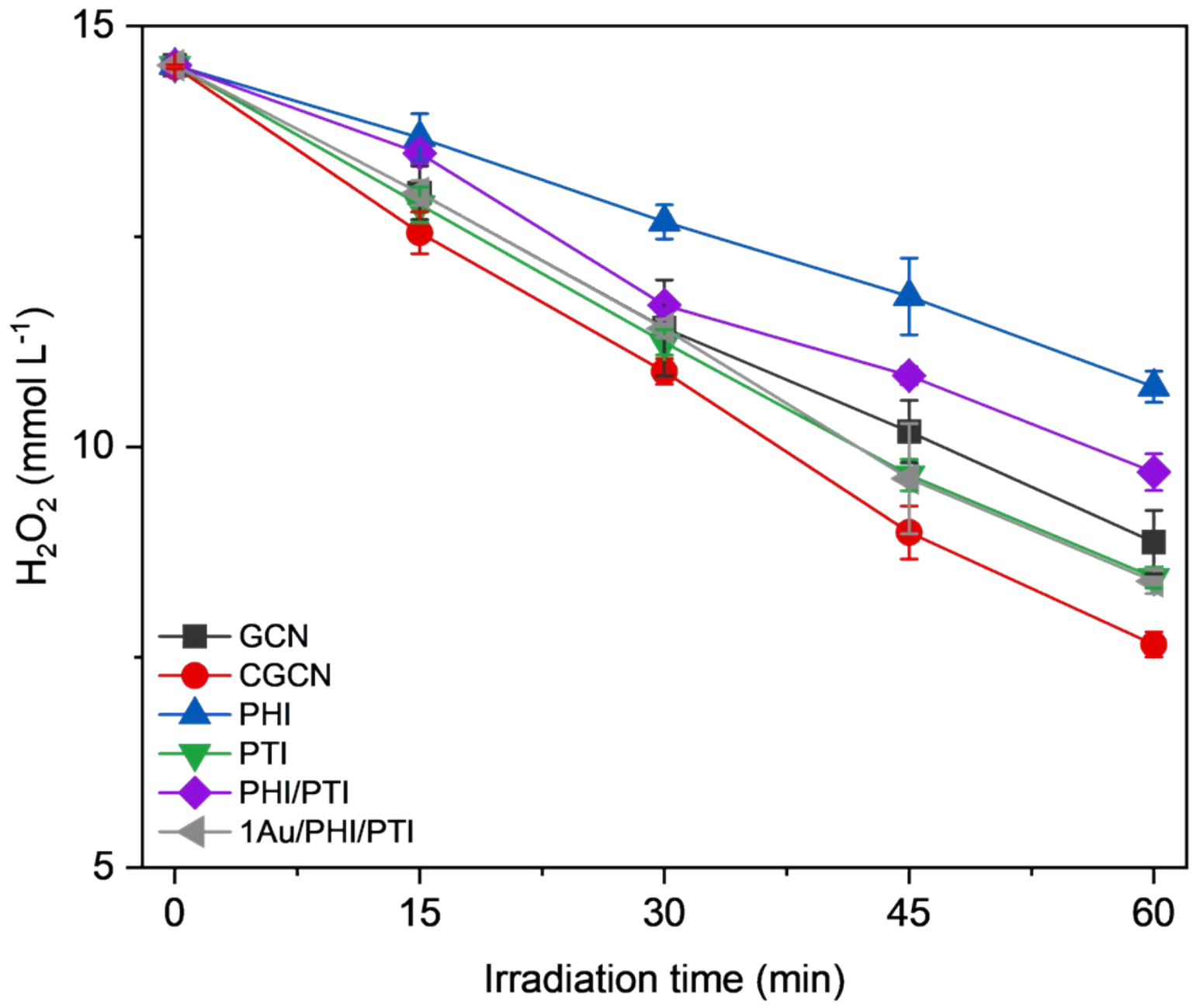

Figure S17. Photocatalytic H_2O_2 decomposition by GCN, CGCN, PHI, PTI, PHI/PTI, and 1Au/PHI/PTI under UV-visible-NIR.

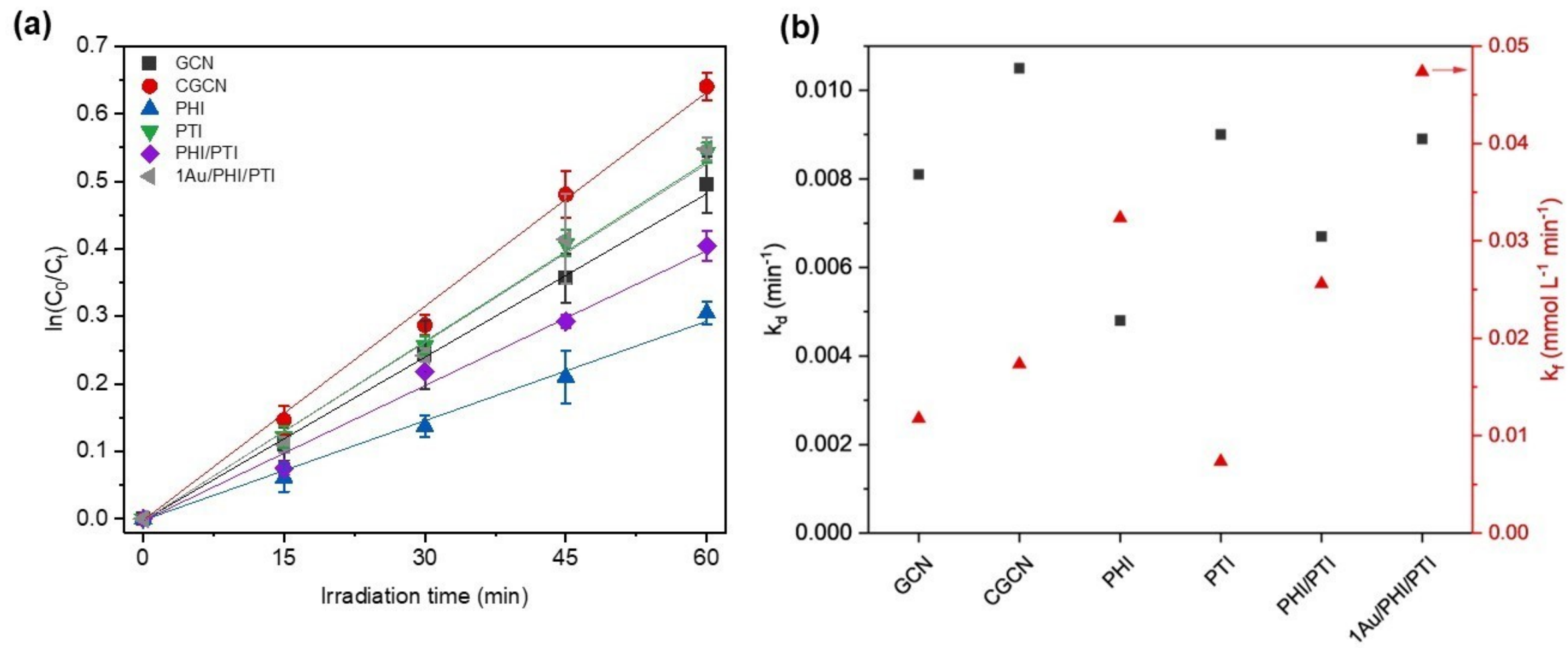

Figure S18. (a) Kinetic plots of photocatalytic H_2O_2 decomposition and (b) decomposition (k_d) and formation (k_f) rate constants by GCN, CGCN, PHI, PTI, PHI/PTI, and 1Au/PHI/PTI under UV-visible-NIR.

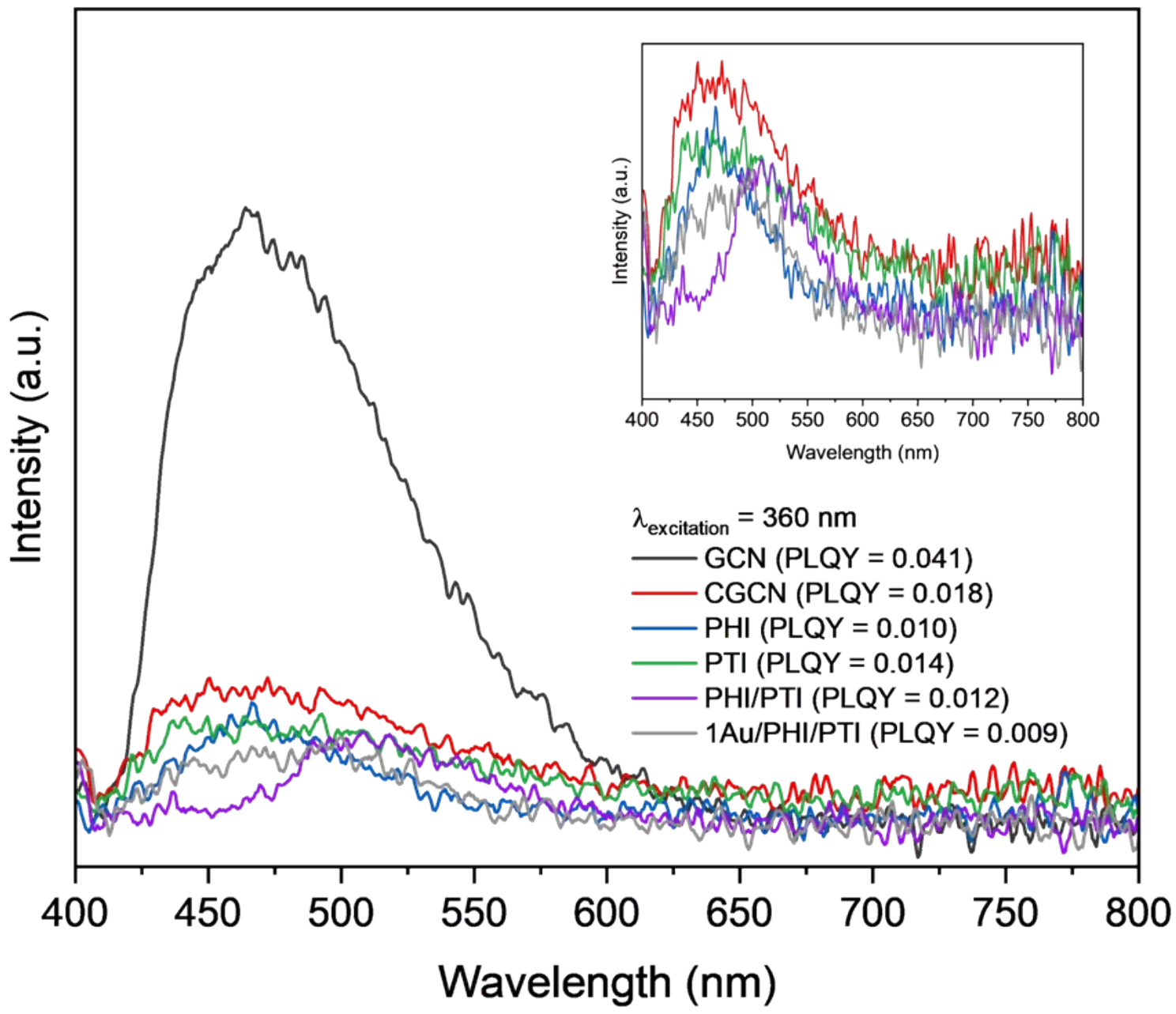

Figure S19. Steady-state photoluminescence spectra of GCN, CGCN, PHI, PTI, PHI/PTI, and 1Au/PHI/PTI photocatalysts after exciting with 360 nm light**.**

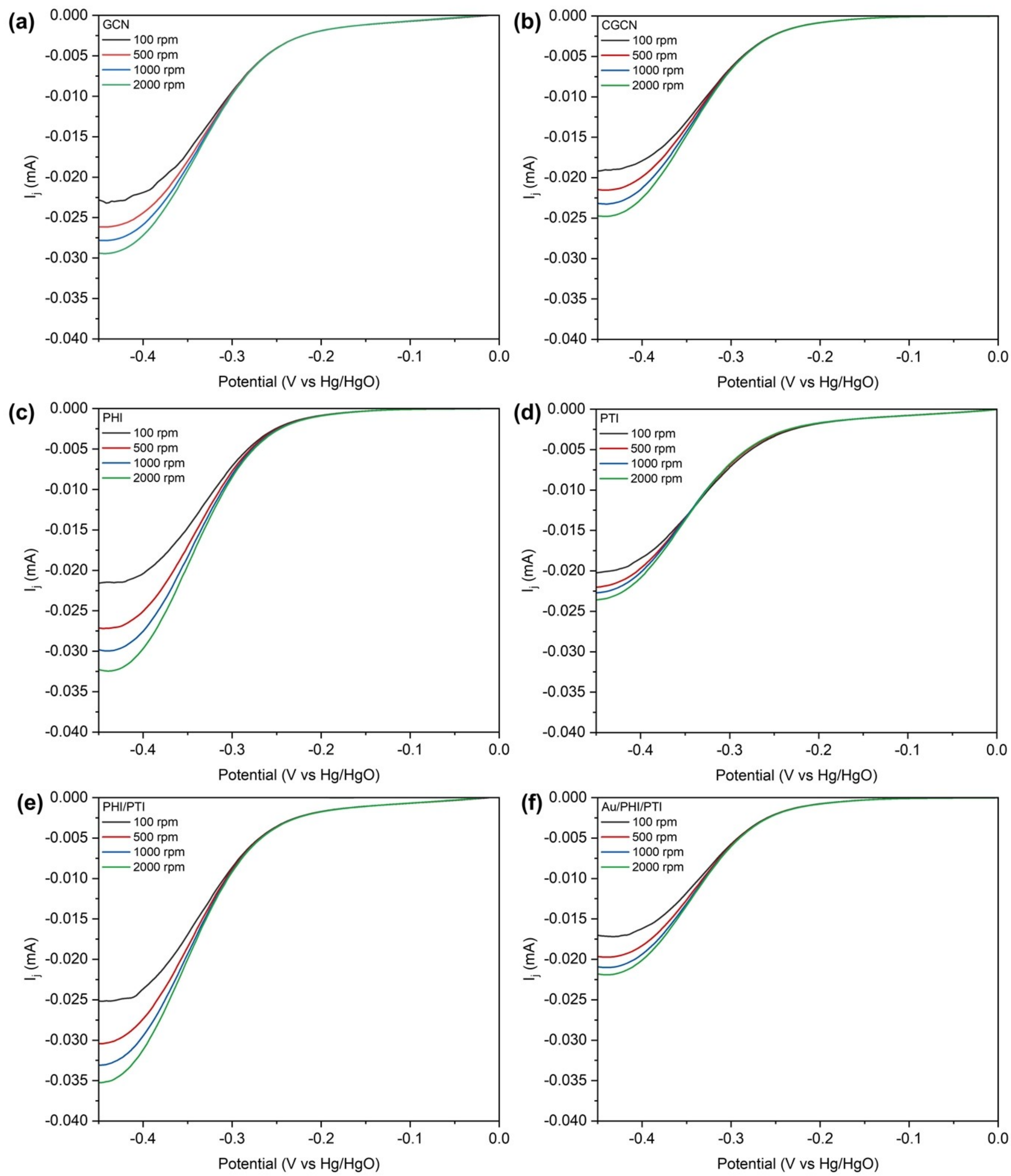

Figure S20. O_2 reduction linear sweep voltammetry (LSV) of (a) PHI/PTI and (b) Au/PHI/PTI samples.

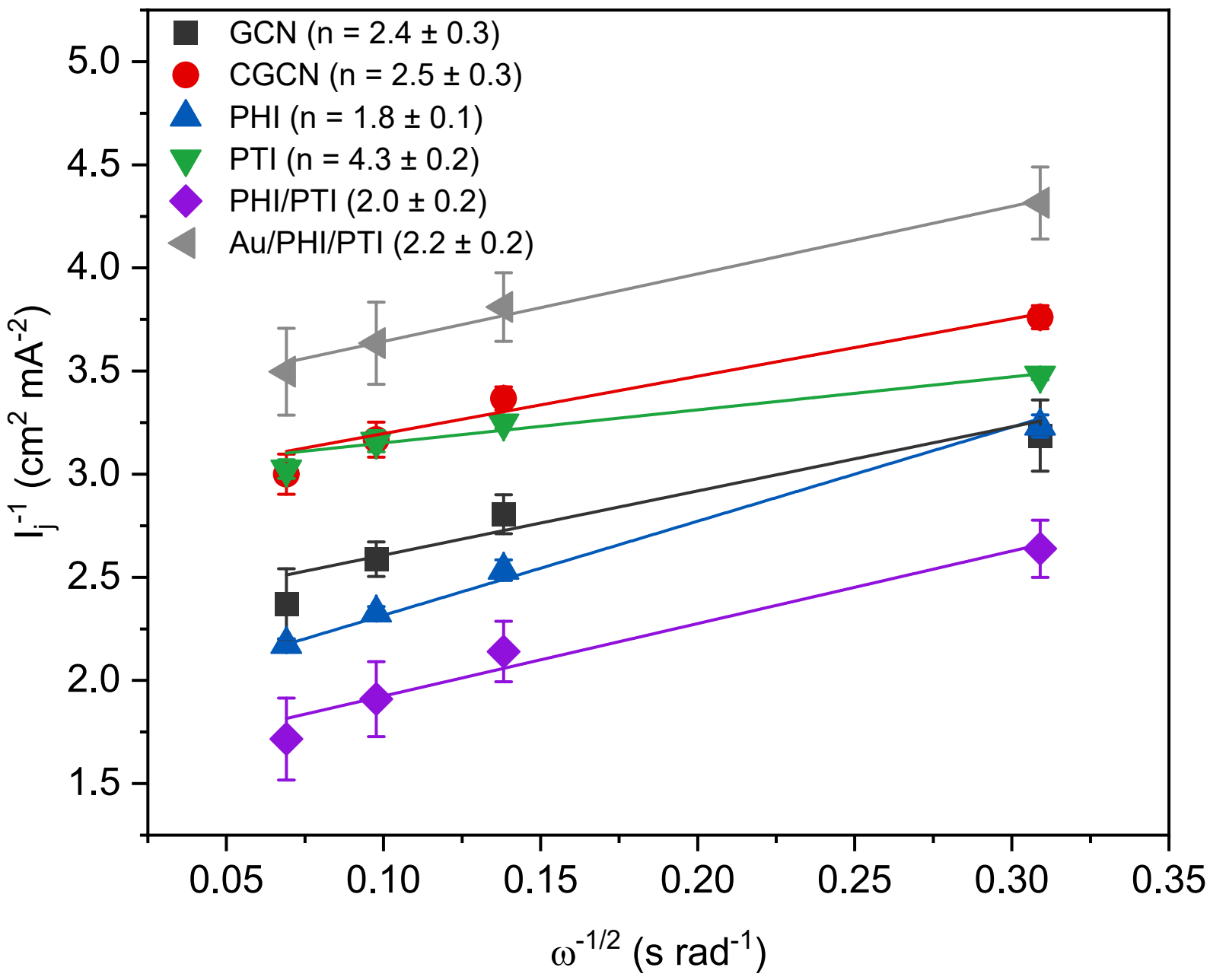

Figure S21. The Koutecky–Levich plots showing the inverse measured current density (I_j^{-1}) versus the inverse square root of the angular rotation rate of the electrode ($\omega^{-1/2}$) of the data obtained using a rotative ring disk electrode (RRDE) in a NaOH solution (0.1 M, pH 12) saturated with O_2 at -0.45 V *vs* Hg/HgO.

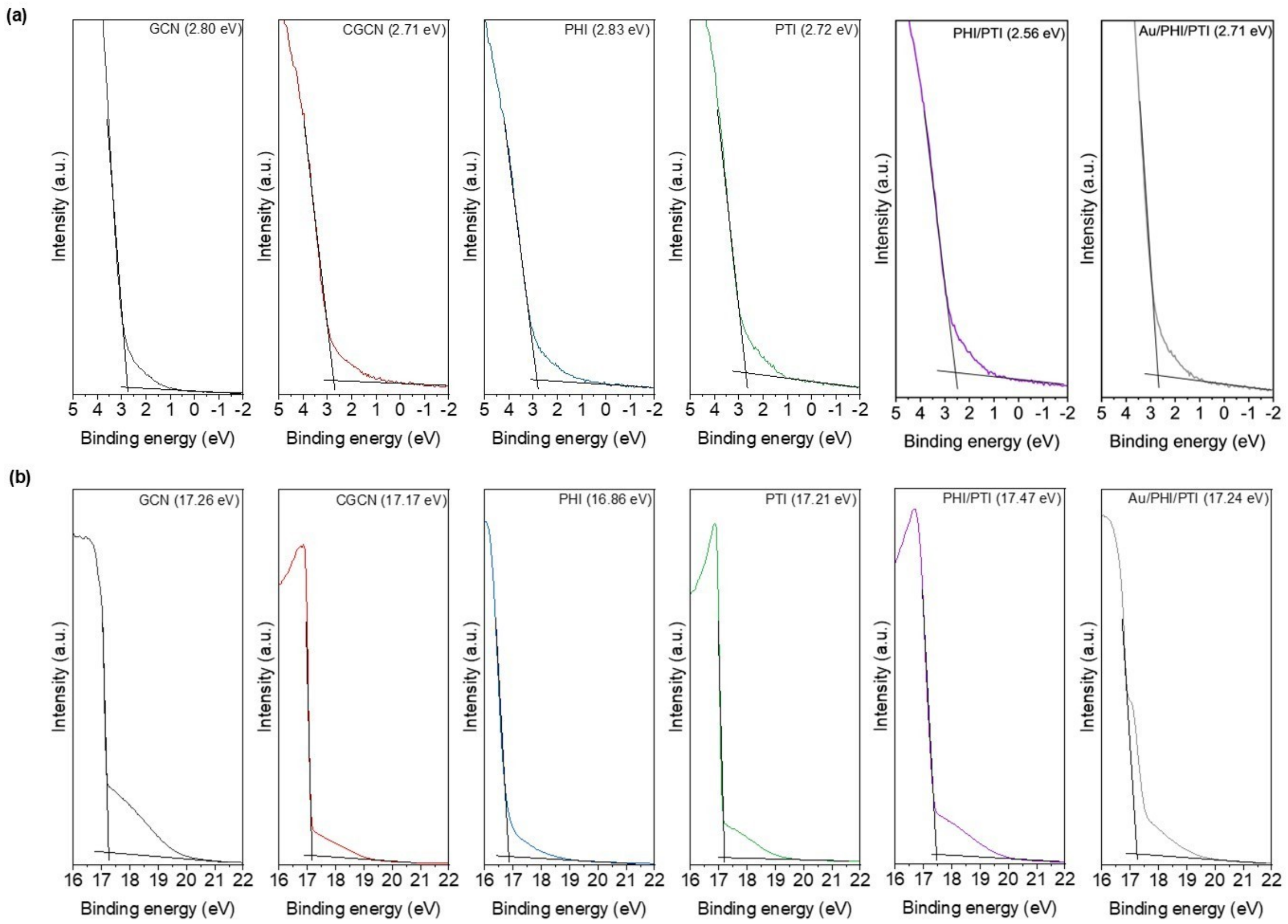

Figure S22. Ultraviolet photoelectron spectroscopy (UPS) spectra of GCN, CGCN, PHI, PTI, PHI/PTI, and Au/PHI/PTI photocatalysts in (a) valence band edge and (b) secondary electron regions.

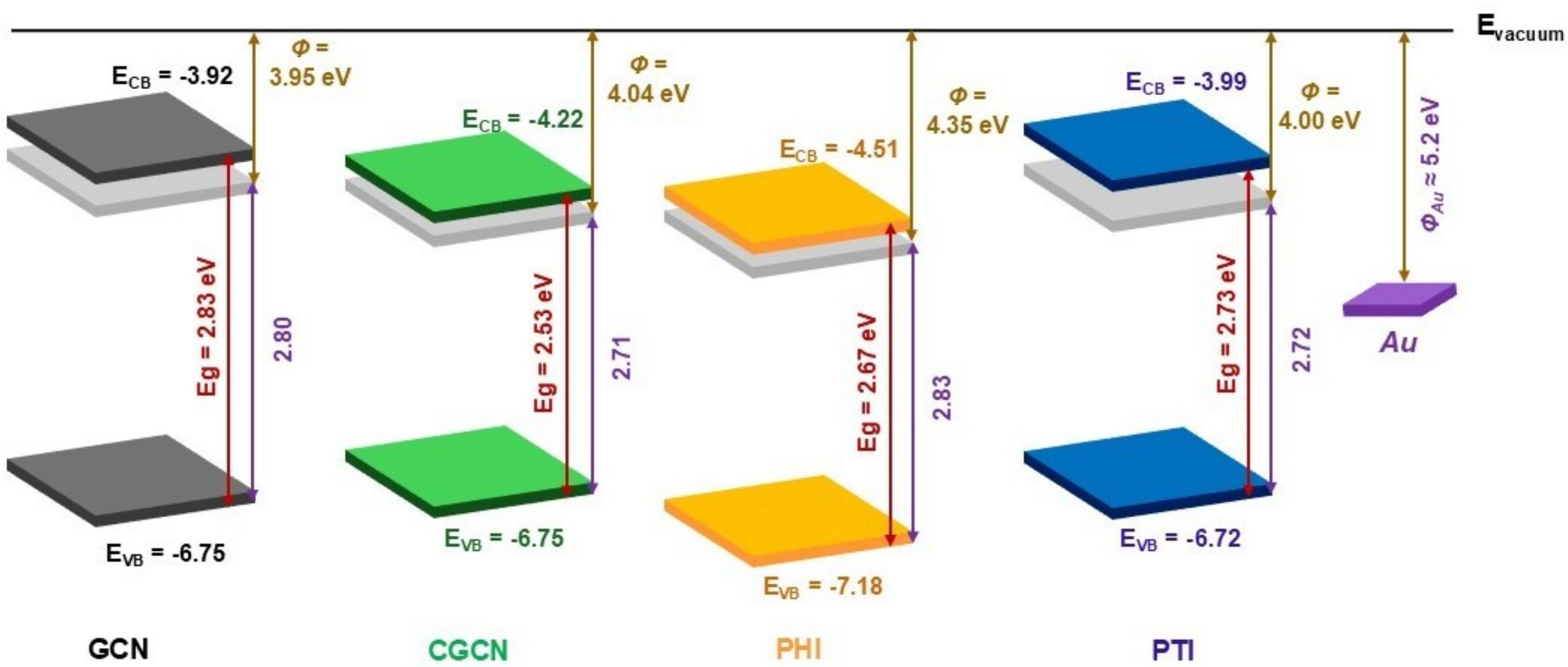

Figure S 23. Calculated band potentials of GCN, CGCN, PHI, and PTI.

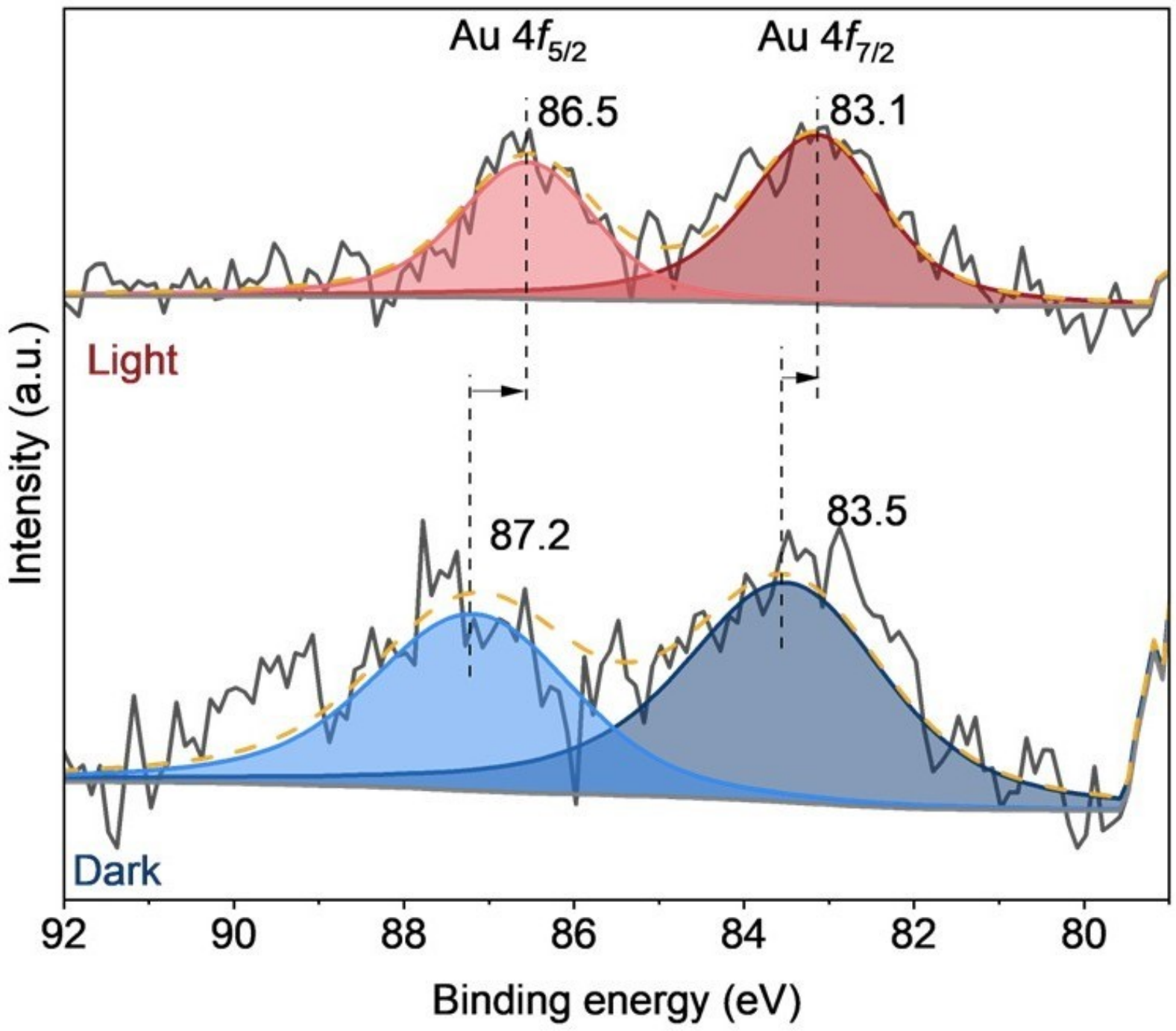

Figure S24 In situ XPS spectra of Au 4f under dark and light illumination**.**

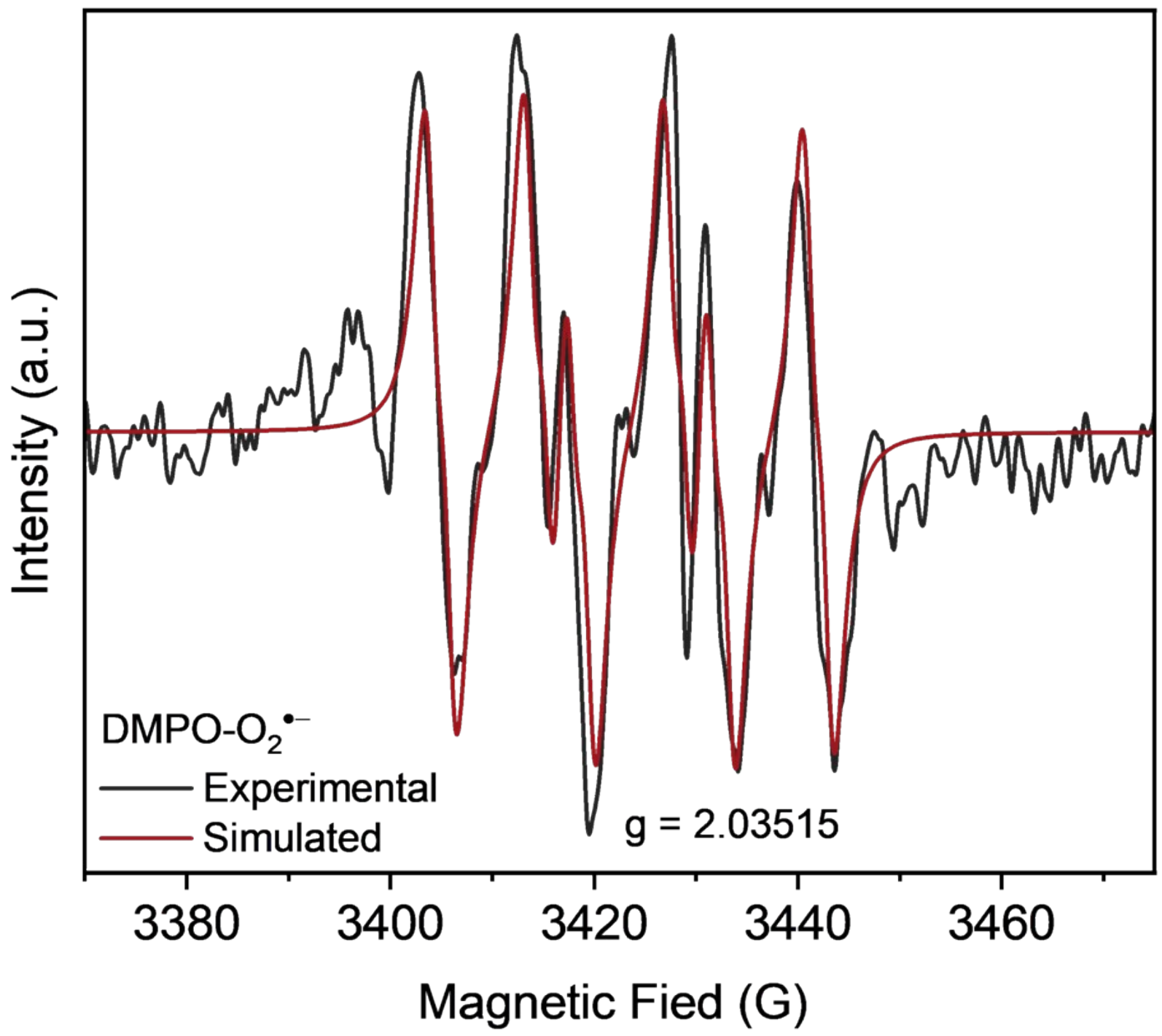

Figure S25. Experimental and simulated EPR spectra of DMPO-$O_2^{\bullet -}$ 1Au/PHI/PTI photocatalyst in O_2-saturated TEOA (5 v/v%) under light irradiation.

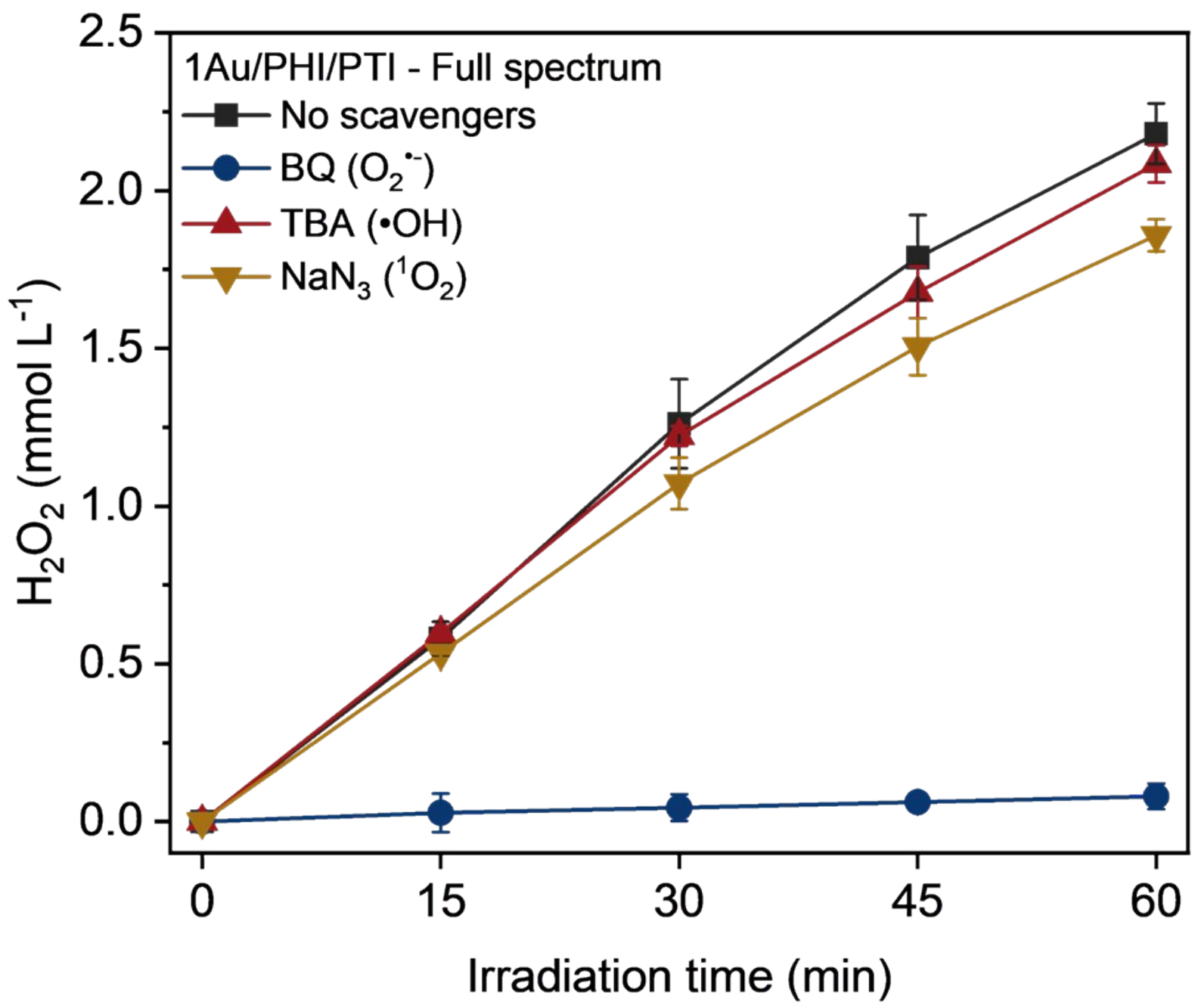

Figure S26 The photocatalytic H_2O_2 production over 1Au/PHI/PTI with different scavengers.

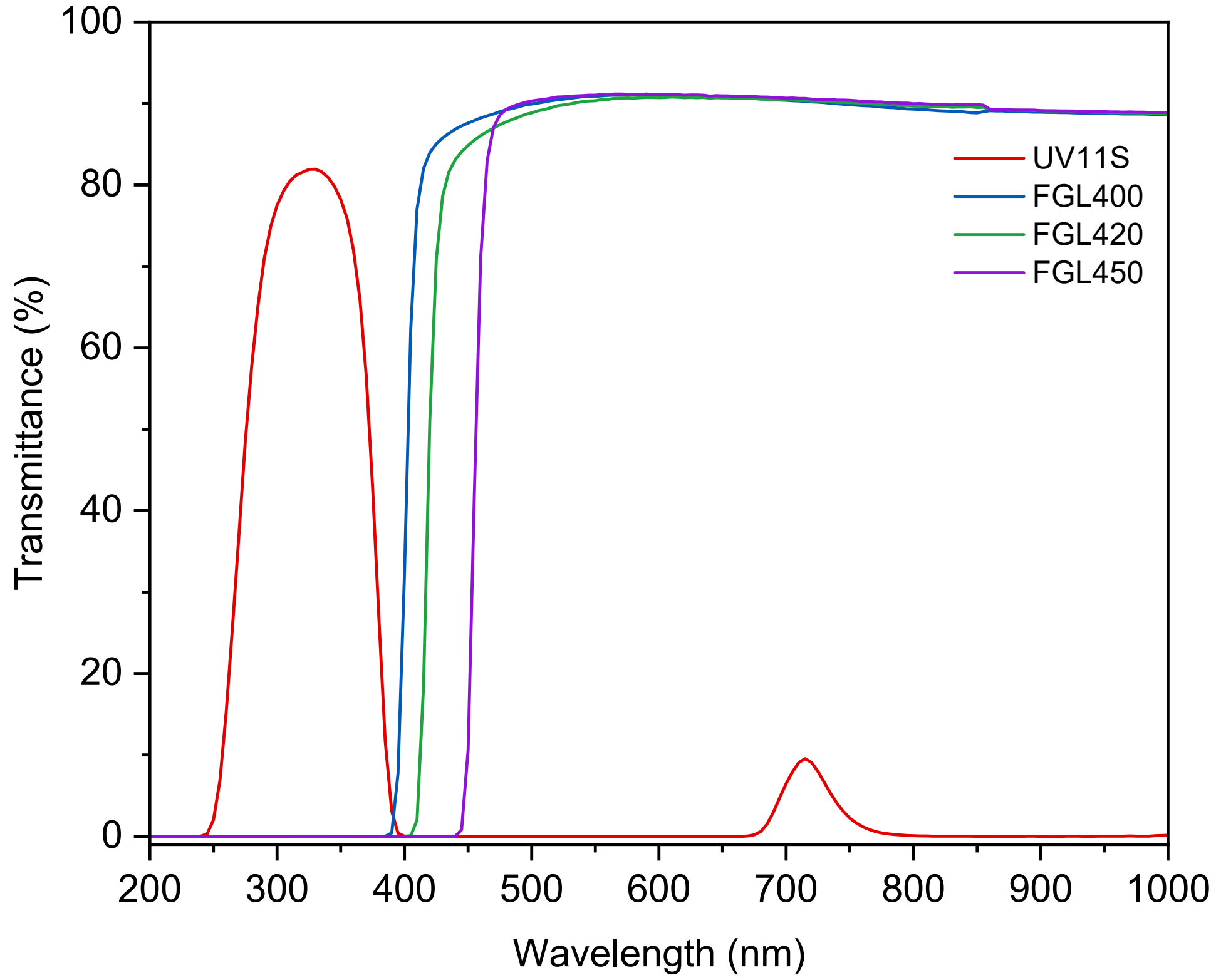

Figure S27. Light transmission data of different filters obtained from ThorLabs.

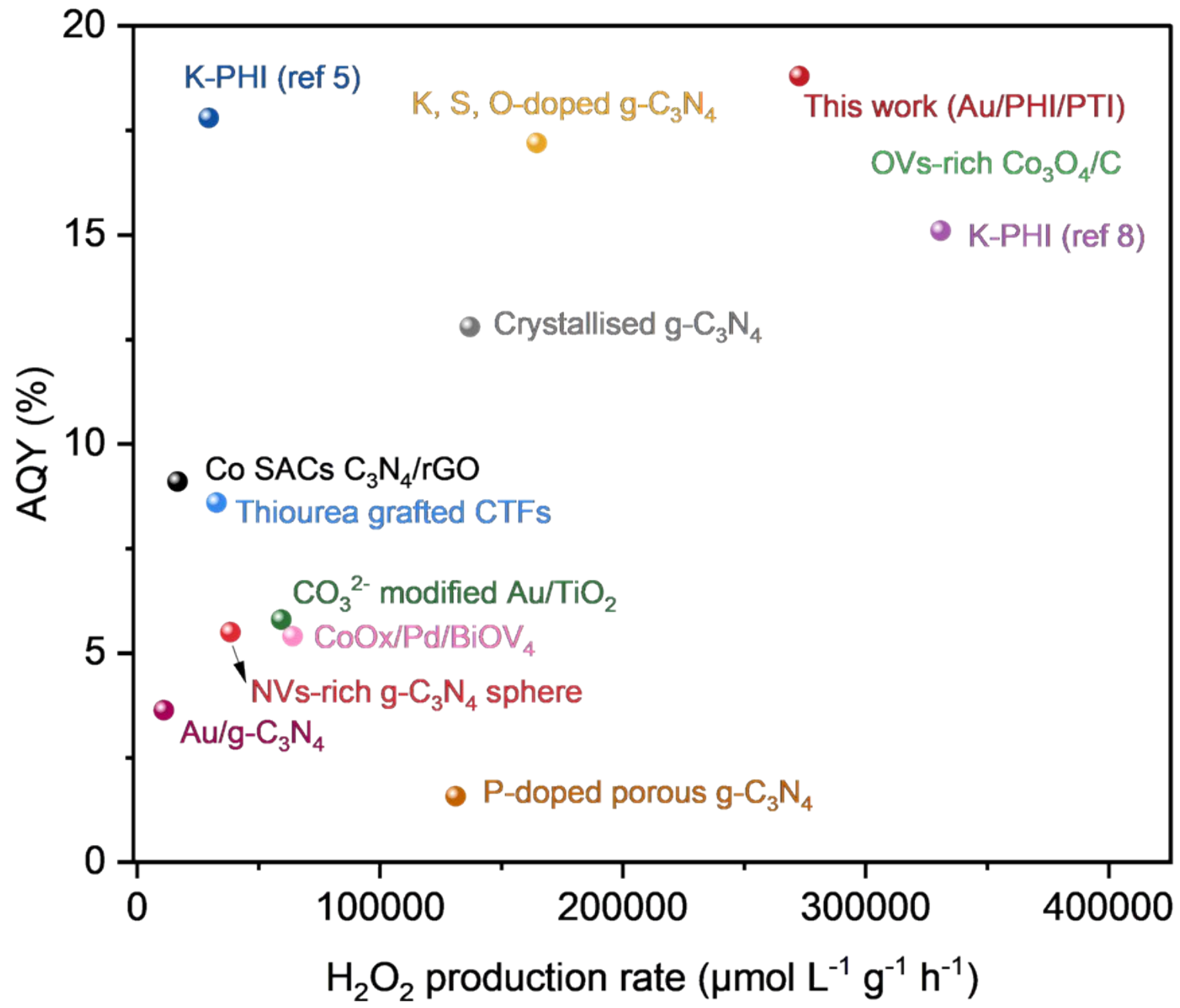

Figure S28. A comparison of the photocatalytic H_2O_2 production rate and AQY obtained from Au/PHI/PTI under the optimum system with the previously reported photocatalysts. References and reaction conditions are provided in Table S7.

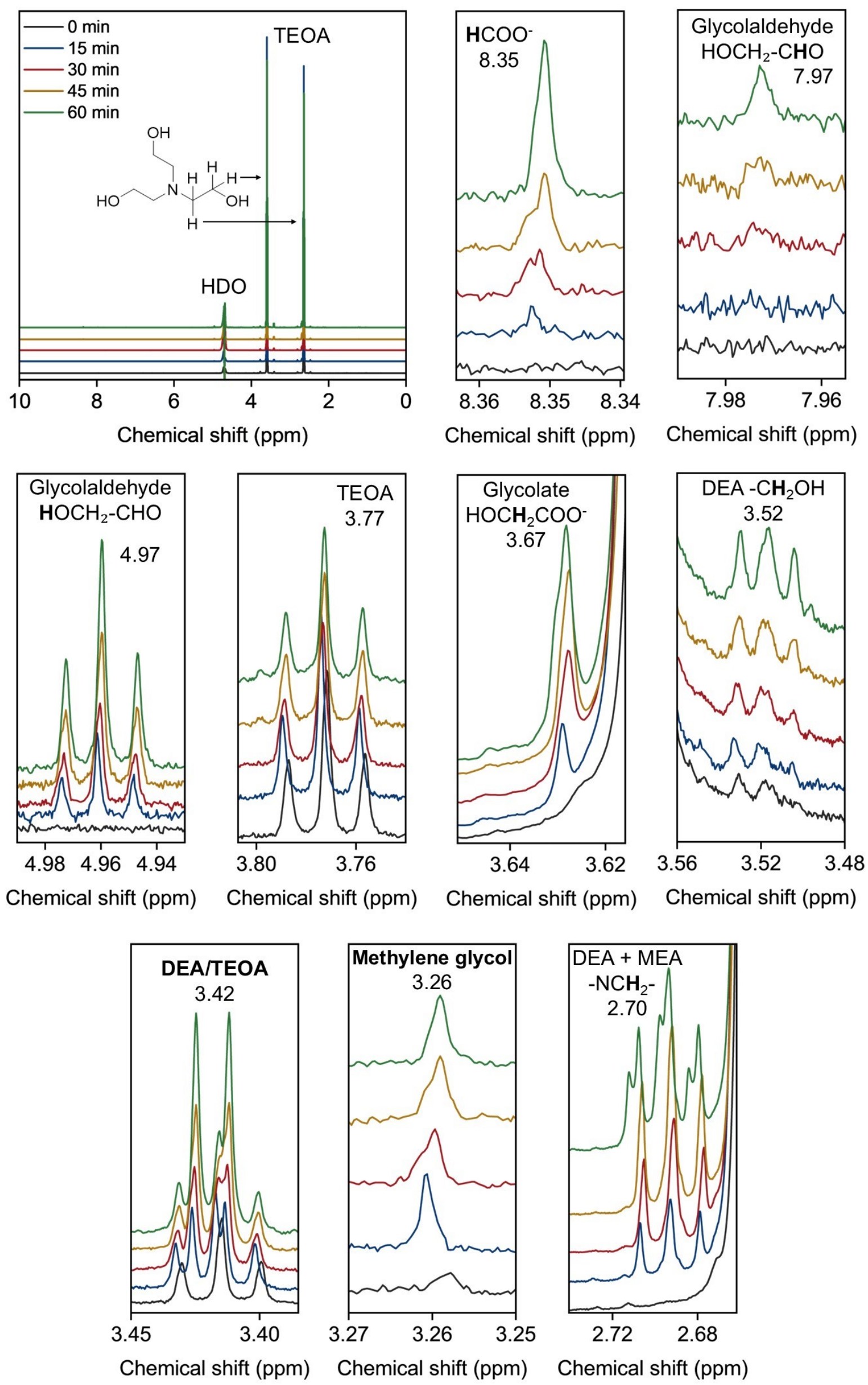

Figure S29. ^{1}H NMR spectra of the solution during photocatalytic H_2O_2 production in the presence of TEOA over the 1Au/PHI/PTI photocatalyst under full-spectrum irradiation.

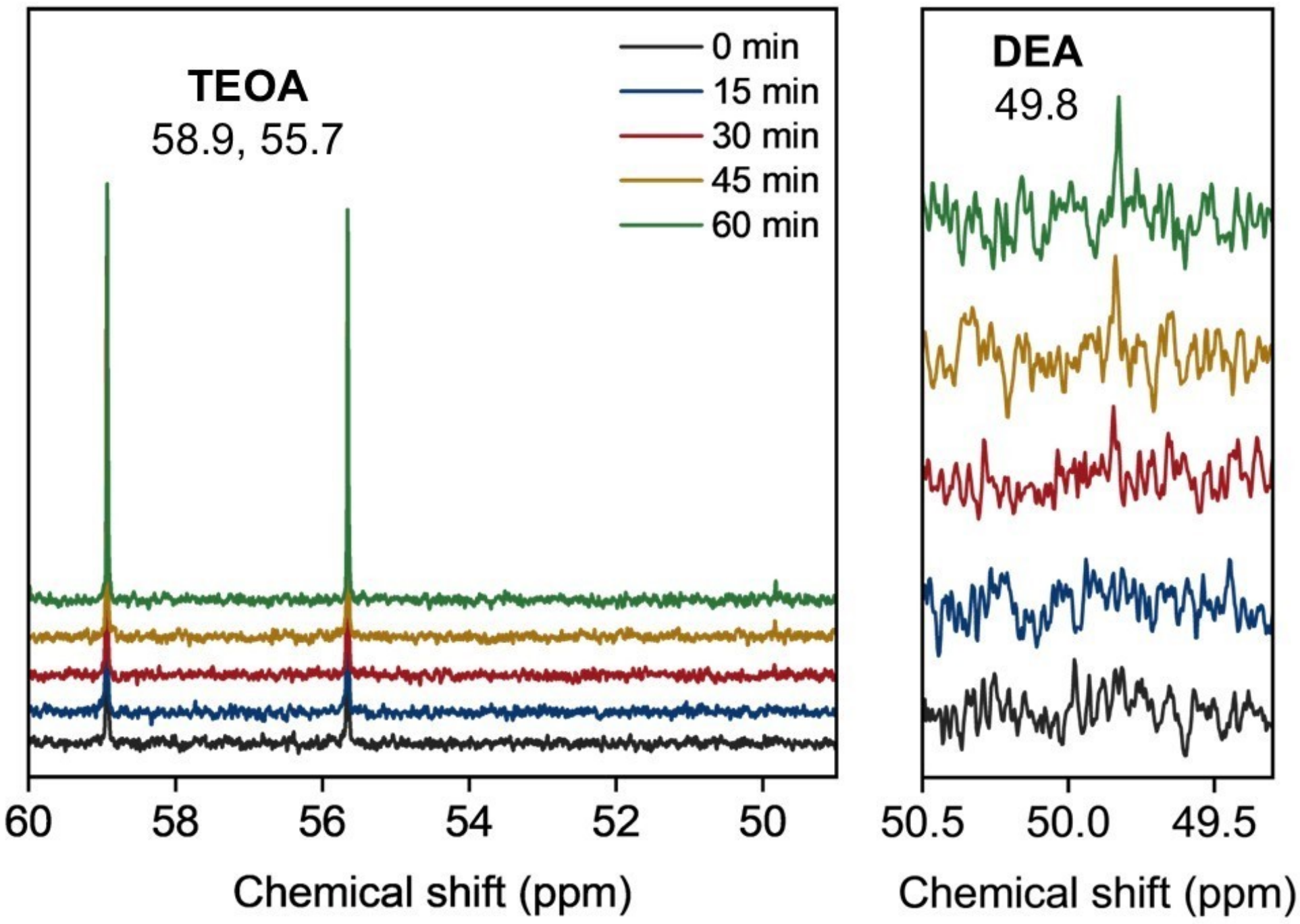

Figure S30. ^{13}C NMR spectra of the solution during photocatalytic H_2O_2 production in the presence of TEOA over the 1Au/PHI/PTI photocatalyst under full-spectrum irradiation.

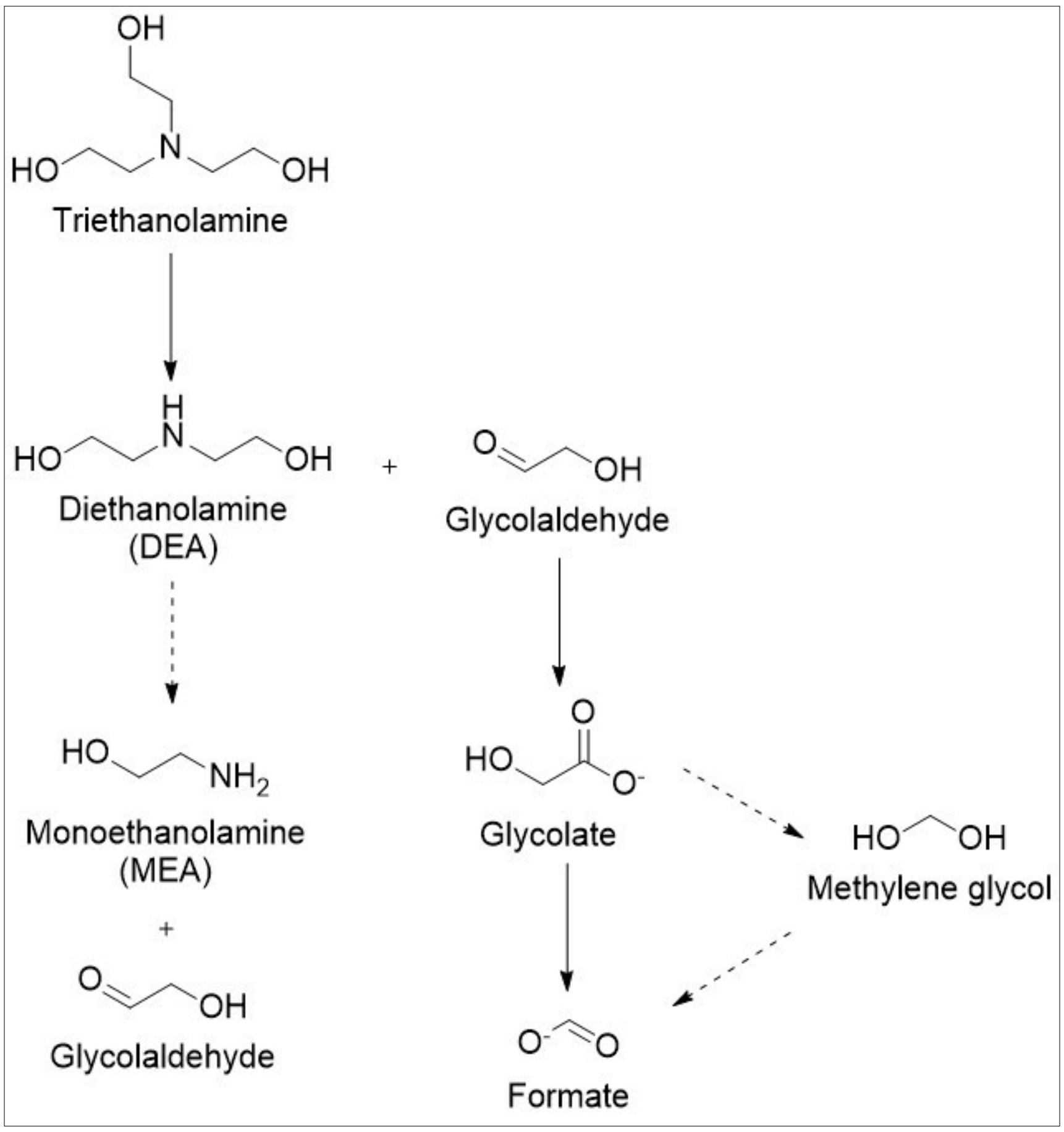

Figure S31. Proposed TEOA valorisation mechanism during photocatalytic H_2O_2 production over the 1Au/PHI/PTI photocatalyst under full-spectrum irradiation.

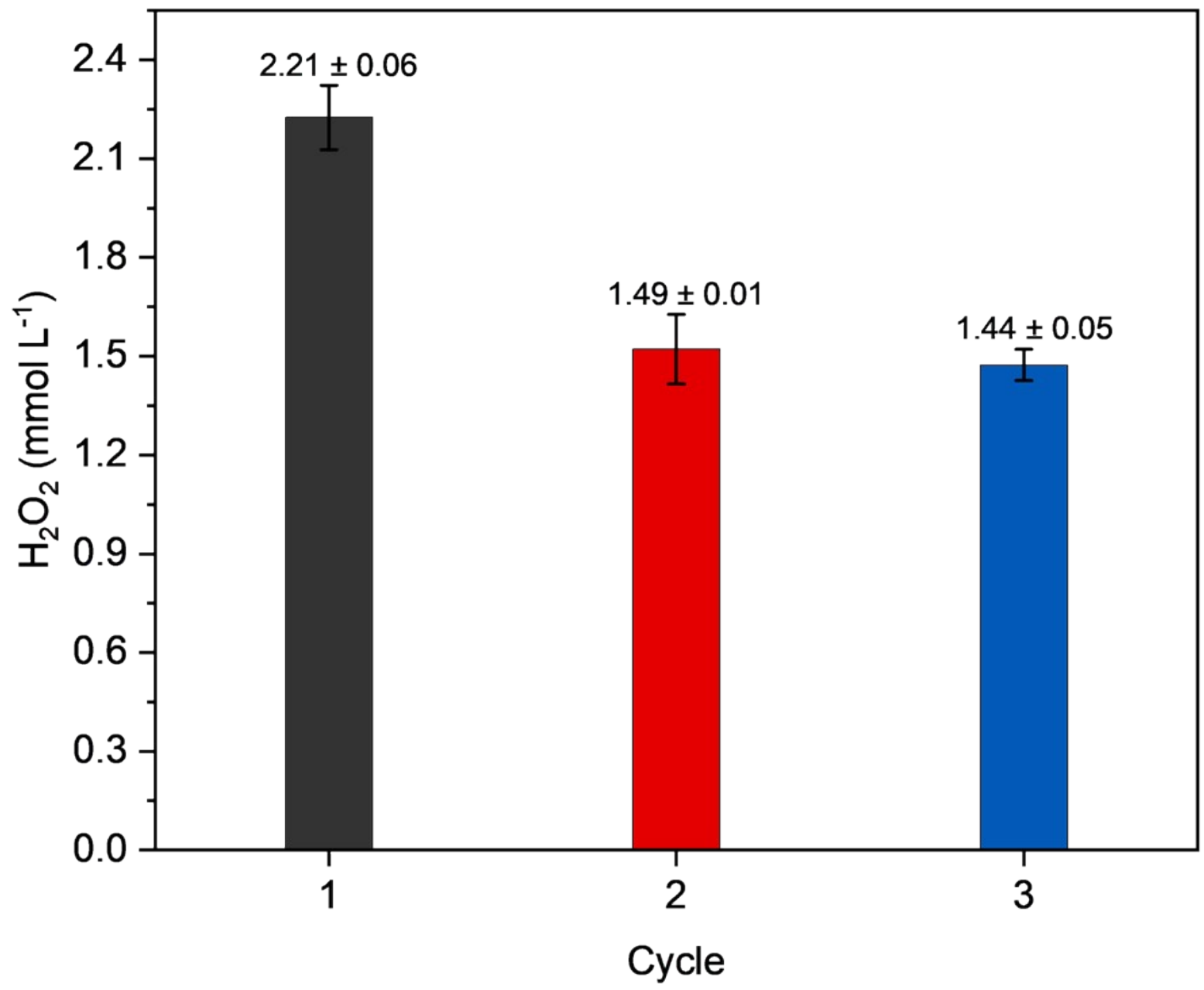

Figure S32. Recyclability of the 1Au/PHI/PTI photocatalyst over three cycles

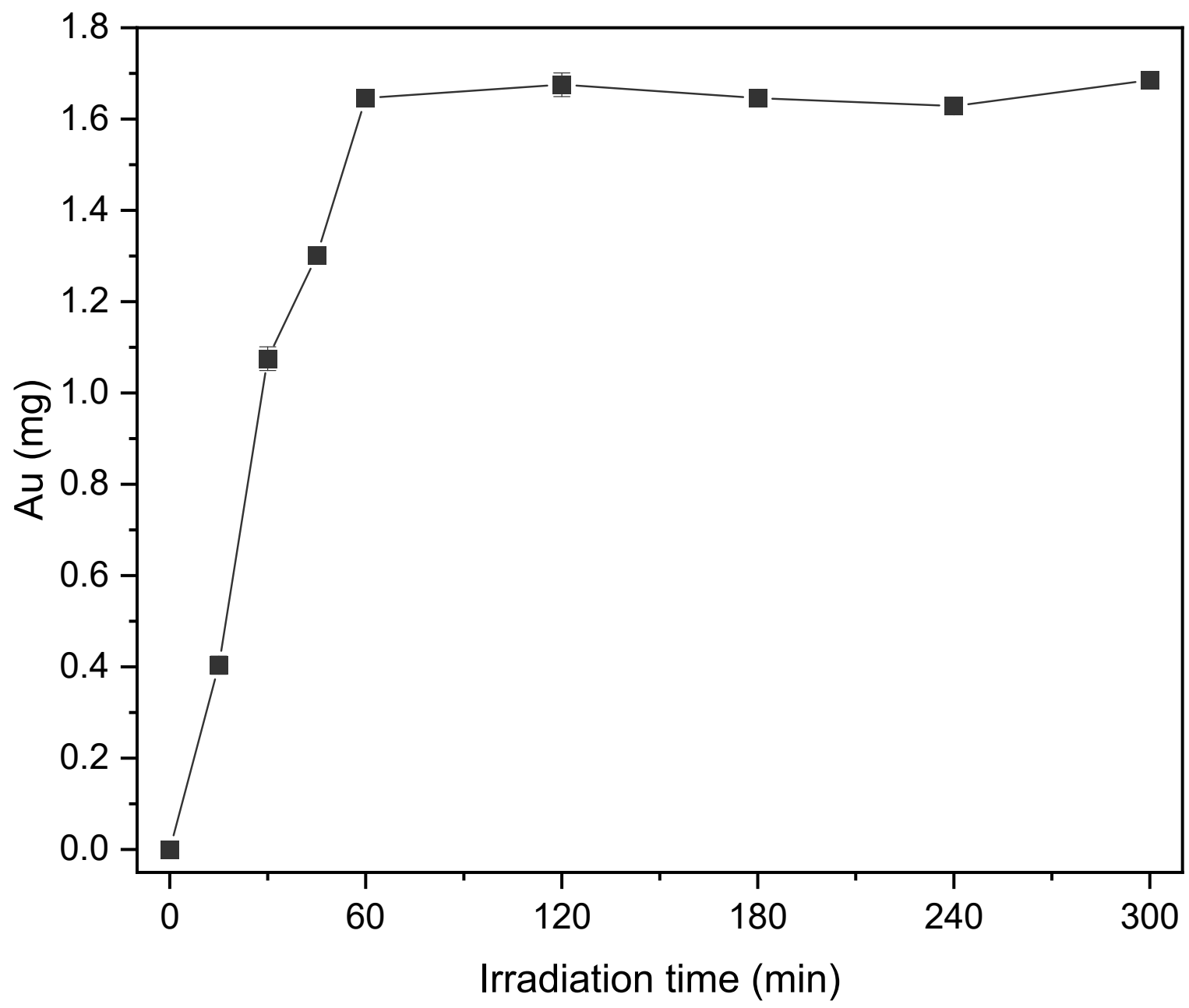

Figure S33. Amount of gold leached into the solution during the reaction, as determined by ICP-MS.

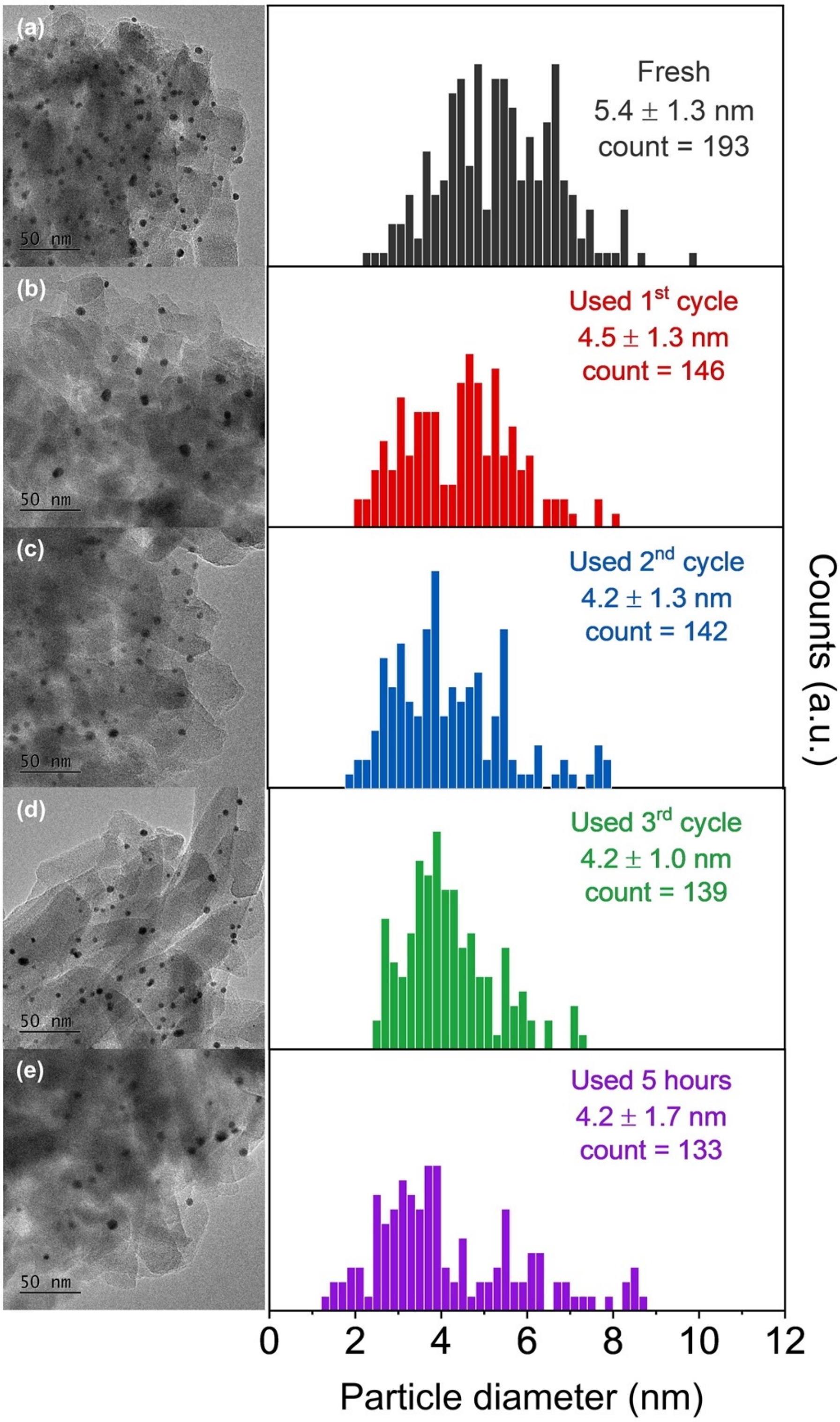

Figure S34. TEM images and particle size distributions of the (a) fresh and used 1Au/PHI/PTI after (b) 1st, (c) 2nd, (d) 3rd cycle, and (e) five-hour run.

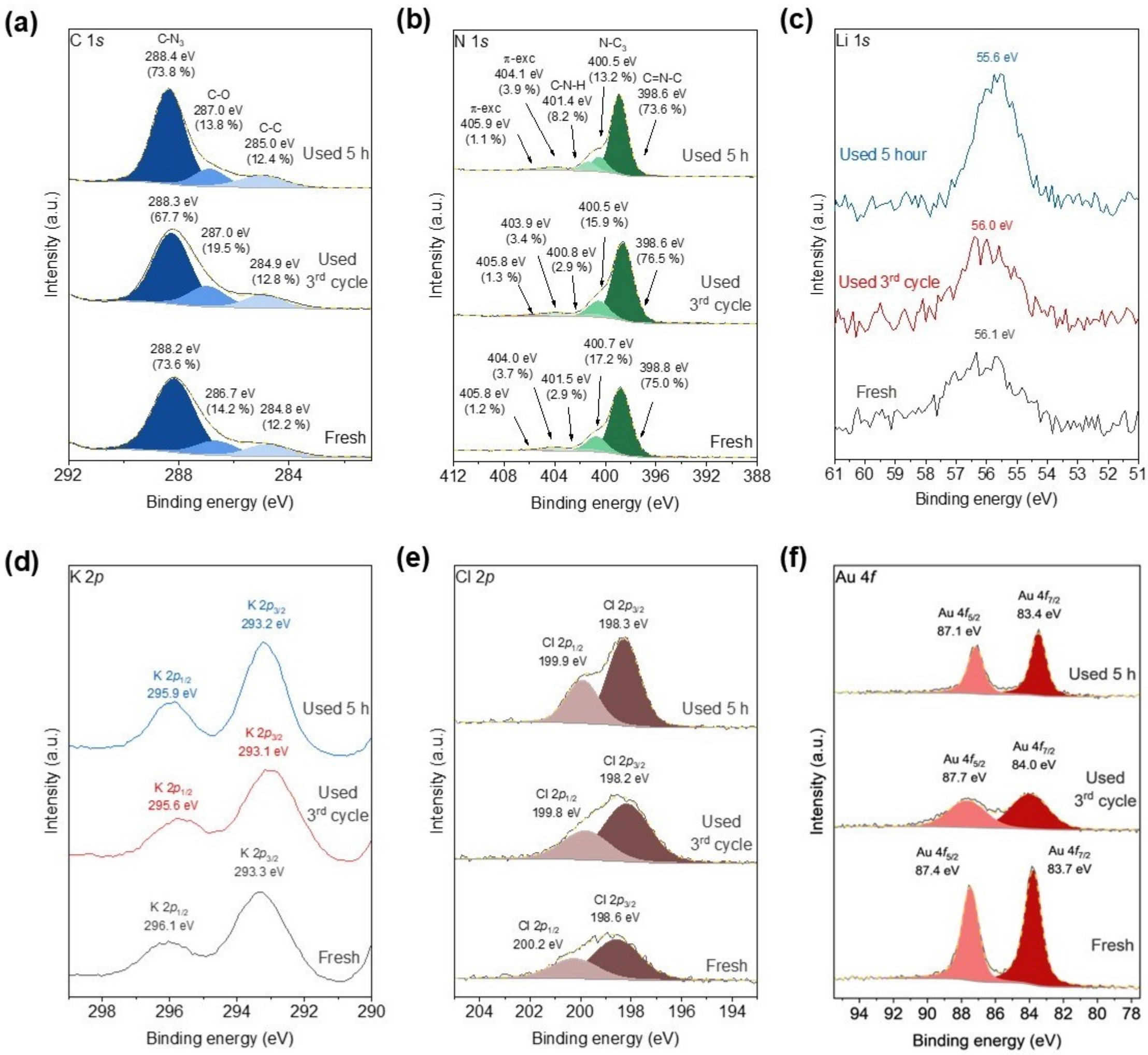

Figure S35. High-resolution (a) C 1*s*, (b) N 1*s*, (c) Li 1*s*, (d) K 2*p*, (e) Cl 2*p*, and (f) Au 4*f* XPS spectra of fresh and used 1Au/PHI/PTI after the 3rd cycle and 5 hours experiment.

Table S1 Quantities of additives used for synthesising the carbon-doped g-C_3N_4 and g-C_3N_4 derivatives.

Sample	KCl (g)	LiCl (g)	Malonic acid (g)
g-C_3N_4 (GCN)	0	0	0
C-doped g-C_3N_4 (CGCN)	0	0	0.6
Poly(heptazine) imide (PHI)	10	0	0.6
Poly(triazine) imide (PHI)	0	10	0.6
C-doped Poly(heptazine) imide/ Poly(triazine) imide (PHI/PTI)	5.5	4.5	0.6

Table S2. Binding energies and compositions of the deconvoluted XPS N 1*s* and C 1*s* signals, excluding π-excitation of GCN, CGCN, PHI, PTI, and PHI/PTI.

**Sample**	**N 1*s***				**C 1*s***		
	**C=N-C**	**N-(C)$_3$**	**C-NH**	**%C=N-C/%C-NH**	**C-C**	**C-O**	**C-N**
GCN	398.7, 72.4%	400.0, 12.4%	401.2, 10.4%	7.0	284.9, 2.5%	286.6, 2.0%	288.2, 95.5%
CGCN	398.7, 71.0%	400.0, 13.5%	401.0, 10.5%	6.8	284.8, 8.9%	286.8, 11.6%	288.2, 79.5%
PHI	398.7, 73.3%	400.4, 11.4%	401.3, 9.7%	7.6	284.7, 4.4%	286.7, 7.8%	288.3, 87.7%
PTI	398.9, 66.4%	-	400.5, 28.6%	2.3	284.8, 3.4%	286.7, 5.4%	288.3, 91.2%
PHI/PTI	398.7, 79.8%	400.5, 11.4%	400.9, 3.9%	20.5	284.8, 8.7%	287.0, 11.2%	288.3, 80.0%
1Au/PHI/PTI	398.8, 75.0%	400.7, 17.2%	401.5, 2.9%	25.9	284.8, 12.2%	286.7, 14.2%	288.2, 73.7%

Table S3. Elemental compositions of GCN, CGCN, PHI, PTI, and /PHI/PTI obtained from XPS and C,H, N elemental analyses.

**Sample**	**XPS**							**CHN**		
	**C (%at)**	**N (%at)**	**K (%at)**	**Li (%at)**	**Cl (%at)**	**Au (%at)**	**C/N**	**C (%at)**	**N (%at)**	**C/N**
GCN	41.9	58.1	-	-	-	-	0.72	33.1	50.4	0.66
CGCN	44.5	55.5	-	-	-	-	0.80	33.5	48.8	0.69
PHI	40.0	54.4	5.4	-	0.2	-	0.74	30.2	46.2	0.65
PTI	38.8	52.5	-	6.7	2.0	-	0.74	30.2	46.2	0.65
PHI/PTI	45.4	44.6	2.1	5.6	2.3	-	1.02	32.4	47.8	0.68
1Au/PHI/PTI	47.7	43.8	1.9	5.7	0.7	0.2	1.09	33.8	51.0	0.66

Table S4. H_2O_2 production over GCN, CGCN, PHI, PTI, PHI/PTI, and 1Au/PHI/PTI under full spectrum and λ > 420 nm irradiation and catalyst synthesis yields.

**Photocatalyst**	**Full spectrum (mmol L^{-1})**	**λ > 420 nm (mmol L^{-1})**	**Catalyst synthesis yield (%)[a]**
GCN	0.56 ± 0.03	0.03 ± 0.01	2.8
CGCN	0.77 ± 0.04	0.19 ± 0.01	4.8
PHI	1.69 ± 0.03	0.37 ± 0.02	2.8
PTI	0.34 ± 0.01	N/D[b]	19.0
PHI/PTI	1.27 ± 0.06	0.41 ± 0.03	23.0
1Au/PHI/PTI	2.21 ± 0.06	0.42 ± 0.02	-

[a] Catalyst synthesis yield (%) = 100 * (weight of product)/(weight of urea used for the synthesis *i.e.* 20 g). [b]N/D: Not detected

Table S5. H_2O_2 production and calculated k_f and k_d values of GCN, CGCN, PHI, PTI, PHI/PTI, and 1Au/PHI/PTI under UV-visible-NIR irradiation.

**Photocatalyst**	**H_2O_2 (mmol L^{-1})**	**k_f (mmol L^{-1} min^{-1})**	**k_d (min^{-1})**
GCN	0.56 ± 0.03	0.012	0.0081
CGCN	0.77 ± 0.04	0.017	0.010
PHI	1.69 ± 0.03	0.032	0.0048
PTI	0.34 ± 0.01	0.007	0.0090
PHI/PTI	1.27 ± 0.06	0.025	0.0067
1Au/PHI/PTI	2.21 ± 0.06	0.047	0.0089

Table S6. Band energies obtained from UPS and UV-vis DRS analyses. All values except Eg are relative to the vacuum level.

Sample	SEC	WF	VBM relative to E_F	E_{VB}	E_g	E_{CB}
GCN	17.26	3.95	2.80	-6.75	2.83	-3.92
CGCN	17.17	4.04	2.71	-6.75	2.53	-4.22
PHI	16.86	4.35	2.83	-7.18	2.67	-4.51
PTI	17.21	4.00	2.72	-6.72	2.73	-3.99
PHI/PTI	17.47	3.74	2.56	-	-	-
1Au/PHI/PTI	17.24	3.97	2.71	-	-	-

Table S7. Comparison of the photocatalytic H_2O_2 production rate and AQY of Au/PHI/PTI and previously reported photocatalysts.

**Photocatalyst**	**Reaction condition**	**H_2O_2 production rate (μmol L^{-1} h^{-1} g^{-1})**	**AQY (%)**
**1Au/PHI/PTI (This work)**	**0.5 v/v% TEOA/H_2O 80 mL, catalyst 0.1 g L^{-1}, 300 W Xe lamp**	**272625**	**18.8**
Au/g-C_3N_4[3]	5 v/v% isopropanol/H_2O 1 mL (pH 3), catalyst 1 g L^{-1}, 300 W Xe lamp	11000	3.63
NVs-rich g-C_3N_4 sphere[4]	10 v/v% isopropanol/H_2O 50 mL, catalyst 0.5 g L^{-1}, Solar simulator (300 W)	38450	5.5
K-PHI[5]	5 v/v% Ethanol/H_2O 50 mL, catalyst 0.2 g L^{-1}, Solar simulator	29500	17.8
Crystalised g-C_3N_4[6]	5 v/v% Ethanol/H_2O 50 mL, catalyst 0.2 g L^{-1}, Solar simulator (160 mW cm^{-2})	137000	12.8
K, S, O-doped g-C_3N_4[7]	10 v/v% Ethanol/H_2O 100 mL, catalyst 0.5 g L^{-1}, 300 W Xe lamp ($\lambda > 420$ nm)	164600	17.2

K-PHI[8]	5 v/v% Ethanol/H_2O 50 mL, catalyst 0.2 g L^{-1}, Solar simulator	331000	15.1
Facet-selective CoO_x and Pd doped $BiVO_4$[9]	1 M Phosphate buffer (pH 7.4) 12 mL, catalyst 2 g L^{-1}, 300 W Xe lamp (100 mW cm^{-2})	59375	5.8
Thiourea grafted CTFs[10]	0.2 M $NaIO_3/H_2O$ 100 mL, catalyst 0.2 g L^{-1}, 300 W Xe lamp	32681	8.6
P-doped porous C_3N_4[11]	H_2O 15 mL, catalyst 0.67 g L^{-1}, Visible light (420 nm $\leq \lambda \leq$ 700 nm)	131200	1.57
Co-SACs g-C_3N_4/rGO[12]	Sea water 50 mL, catalyst 1 g L^{-1}, Solar Simulator AM 1.5	16580	9.1
CO_3^{2-} modified Au NPs/TiO_2[13]	4 v/v% HCOOH/H_2O 10 mL, catalyst 1 g L^{-1}, 300 W Xe lamp (λ > 420 nm)	64000	4.26

Table S 8. Binding energies (eV) and compositions of the deconvoluted XPS C 1*s* and N 1*s* signals, excluding π-excitation, of fresh and used 1Au/PHI/PTI.

Sample	C 1*s*			N 1*s*		
	**C-C**	**C-O**	**C-N**	**C=N-C**	**N-(C)$_3$**	**C-NH**
Fresh	284.8 (12.2%)	286.7 (14.2%)	288.2 (73.7%)	398.8 (75.0%)	400.7 (17.2%)	401.5 (2.9%)
3rd cycle of 1-hour reaction	284.9 (12.8%)	287.0 (19.5%)	288.3 (67.7%)	398.6 (76.5%)	400.5 (15.9%)	400.8 (2.9%)
One five-hour reaction	285.0 (12.4%)	287.0 (13.8%)	288.4 (73.8%)	398.6 (73.6%)	400.5 (13.2%)	401.4 (8.2%)

References

(1) Fattahimoghaddam, H.; Mahvelati-Shamsabadi, T.; Lee, B. K. Enhancement in Photocatalytic H_2O_2 Production over g-C3N4 Nanostructures: A Collaborative Approach of Nitrogen Deficiency and Supramolecular Precursors. *ACS Sustain. Chem. Eng.* 2021, *9* (12), 4520–4530. https://doi.org/10.1021/acssuschemeng.0c08884.

(2) Li, Y.; Gong, F.; Zhou, Q.; Feng, X.; Fan, J.; Xiang, Q. Crystalline Isotype Heptazine-/Triazine-Based Carbon Nitride Heterojunctions for an Improved Hydrogen Evolution. *Appl. Catal. B* 2020, *268*, 118381. https://doi.org/10.1016/j.apcatb.2019.118381.

(3) Chang, X.; Yang, J.; Han, D.; Zhang, B.; Xiang, X.; He, J. Enhancing Light-Driven Production of Hydrogen Peroxide by Anchoring Au onto C_3N_4 Catalysts. *Catalysts* 2018, *8* (4), 1–13. https://doi.org/10.3390/catal8040147.

(4) Zheng, Y.; Luo, Y.; Ruan, Q.; Yu, J.; Guo, X.; Zhang, W.; Xie, H.; Zhang, Z.; Zhao, J.; Huang, Y. Plasma-Tuned Nitrogen Vacancy Graphitic Carbon Nitride Sphere for Efficient Photocatalytic H_2O_2 Production. *J. Colloid Interface Sci.* 2022, *609*, 75–85. https://doi.org/10.1016/j.jcis.2021.12.006.

(5) Yang, Z.; Li, L.; Cui, J.; Shao, S.; Zeng, S.; Wang, K.; Ma, D.; Hu, C.; Zhao, Y. Nanoarchitectonics in the Ionothermal Synthesis for Nucleation of Crystalline Potassium Poly (Heptazine Imide) Towards an Enhanced Solar-Driven H_2O_2 Production. *Chemistry - A European Journal* 2022, *28* (59). https://doi.org/10.1002/chem.202202122.

(6) Zeng, S.; Li, L.; Yang, Z.; Cui, J.; Shao, S.; Wang, K.; Cui, J.; Zhao, Y. Polymeric Carbon Nitride from Inorganic Precursor in Eutectic Salts: Enhanced Exciton Dissociation and Photocatalytic H_2O_2 Production. *Journal of Physical Chemistry C* 2022, *126* (47), 20028–20035. https://doi.org/10.1021/acs.jpcc.2c06361.

(7) Li, S.; Feng, B.; Zhang, X.; Tian, J.; Wang, D.; Pei, Y.; Qiao, M.; Li, Y.; Zong, B. Multiple Heteroatom-Doped Urea and Thiourea-Derived Polymeric Carbon Nitride for High-Performance Visible Light-Driven Photocatalytic O_2 Reduction to H_2O_2. *Appl. Catal. B* 2023, *335*, 122879. https://doi.org/10.1016/j.apcatb.2023.122879.

(8) Yang, Z.; Guo, B.; Hu, Z.; Wang, K.; Cui, J.; Li, L.; Hu, C.; Zhao, Y. Molecular Engineering towards Dual Surface Local Polarization Sites on Poly(Heptazine Imide) Framework for Boosting H_2O_2 Photo-Production. *Chinese Chemical Letters* 2024, *35* (8), 109251. https://doi.org/10.1016/j.cclet.2023.109251.

(9) Liu, T.; Pan, Z.; Vequizo, J. J. M.; Kato, K.; Wu, B.; Yamakata, A.; Katayama, K.; Chen, B.; Chu, C.; Domen, K. Overall Photosynthesis of H_2O_2 by an Inorganic Semiconductor. *Nat. Commun.* 2022, *13* (1), 1–8. https://doi.org/10.1038/s41467-022-28686-x.

(10) Wu, C.; Teng, Z.; Yang, C.; Chen, F.; Yang, H. Bin; Wang, L.; Xu, H.; Liu, B.; Zheng, G.; Han, Q. Polarization Engineering of Covalent Triazine Frameworks for Highly Efficient Photosynthesis of Hydrogen Peroxide from Molecular Oxygen and Water. *Advanced Materials* 2022, *34* (28), 1–9. https://doi.org/10.1002/adma.202110266.

(11) Cao, J.; Wang, H.; Zhao, Y.; Liu, Y.; Wu, Q.; Huang, H.; Shao, M.; Liu, Y.; Kang, Z. Phosphorus-Doped Porous Carbon Nitride for Efficient Sole Production of Hydrogen Peroxide: Via Photocatalytic Water Splitting with a Two-Channel Pathway. *J. Mater. Chem. A Mater.* 2020, *8* (7), 3701–3707. https://doi.org/10.1039/c9ta13929j.

(12) Wang, W.; Song, Q.; Luo, Q.; Li, L.; Huo, X.; Chen, S.; Li, J.; Li, Y.; Shi, S.; Yuan, Y.; Du, X.; Zhang, K.; Wang, N. Photothermal-Enabled Single-Atom Catalysts for High-Efficiency Hydrogen Peroxide Photosynthesis from Natural Seawater. *Nat. Commun.* 2023, *14* (1), 1–10. https://doi.org/10.1038/s41467-023-38211-3.

(13) Teranishi, M.; Hoshino, R.; Naya, S.; Tada, H. Gold-Nanoparticle-Loaded Carbonate-Modified Titanium(IV) Oxide Surface:Visible-Light-Driven Formation of Hydrogen Peroxide from Oxygen. *Angew. Chem. Int. Ed.* 2016, *55*, 12773–12777. https://doi.org/10.1002/anie.201606734.
